# Physical activity, entertainment screen time, and dietary fat profiles and concurrent weight status among school-attending Chinese children and adolescents: CHNS 2004–2011

**DOI:** 10.3389/fpubh.2026.1945546

**Published:** 2026-09-28

**Authors:** Boao Qin, Changyang Wang, Yuchen Zhang, Linwei Sheng, Xiaobo Jiang, Chen Cheng, Yukuo Wang

**Affiliations:** School of Physical Education, Yanshan University, Qinhuangdao, China

**Keywords:** behavioral profiles, children and adolescents, China Health and Nutrition Survey, dietary fat, entertainment screen time, latent profile analysis, physical activity, weight status

## Abstract

**Background:**

Physical activity, screen-based leisure, and diet co-occur, but profiles estimated from a small set of indicators may be sensitive to historical period and model assumptions. We identified profiles defined by total physical activity, reported entertainment screen time, and dietary fat contribution to energy intake in the 2004–2011 China Health and Nutrition Survey (CHNS), and examined their concurrent associations with weight status.

**Methods:**

This pooled cross-sectional analysis included 4,112 person-wave observations from school-attending participants aged 6–17 years. A prespecified Gaussian mixture analysis compared one through six profiles. Associations with measured overweight/obesity were estimated in 4,068 observations from 2,757 participants using posterior-probability pseudo-class draws and participant-clustered robust standard errors. Wave-, age-, model-specification-, missing-data-, and alternative-outcome analyses assessed robustness.

**Results:**

The prespecified model retained five profiles: very low activity (6.9%), low-to-moderate activity/lower fat (37.8%), no reported entertainment screen time (5.4%), higher fat (27.8%), and highly active (22.2%). The exact BIC-preferred number of profiles varied across survey waves, age groups, and model specifications, and 48.4% of observations had a maximum posterior probability below 0.70. The estimated prevalence of overweight/obesity, accounting for profile-membership uncertainty, was highest in the very-low-activity profile (24.4%) and was 16.4% in the reference profile. This descriptive difference attenuated after adjustment (odds ratio, 1.18; 95% confidence interval, 0.81–1.69), and the fully adjusted global profile test was non-significant (*p* = 0.806). Multinomial models separating overweight and obesity, continuous BMI and approximate WHO BMI-for-age z-score models, multiple imputation, and inverse-probability weighting produced the same overall conclusion.

**Conclusion:**

In the 2004–2011 CHNS analytic sample, the measured behaviors formed heterogeneous combinations, but the selected profiles did not independently distinguish concurrent weight status after adjustment. The findings are historical and sample-specific; they should not be interpreted as current national profile distributions or as validation of profile-based interventions.

## 1 Introduction

Overweight and obesity during childhood and adolescence are major public health concerns because they are associated with adverse cardiometabolic, musculoskeletal, psychosocial, and longer-term outcomes (1–3). China has experienced a substantial rise in childhood adiposity alongside rapid changes in food systems, transport, schooling, and urban living (4, 5). These changes make modifiable behaviors important to study, but no single behavior captures the context in which energy intake and expenditure occur.

Physical activity, sedentary screen use, and dietary intake share family, school, neighborhood, socioeconomic, and developmental determinants. They may reinforce one another, compete for time, or coexist in mixed combinations. A child may, for example, report high activity together with substantial screen time or a high proportion of energy from fat. Person-centered methods can summarize such co-occurrence without assuming that all favorable or unfavorable behaviors move together (6–8). However, the resulting categories represent only the indicators entered into the model; they should not automatically be treated as complete lifestyle types.

Evidence that behavioral profiles distinguish adiposity is inconsistent. Systematic reviews have identified substantial variation in the behaviors measured, the number and interpretation of classes, outcome definitions, and analytic methods, with many cross-sectional studies reporting no clear association with overweight or obesity (6, 9, 10). Longitudinal evidence on profile stability, transitions, and subsequent adiposity remains limited, although recent work has begun to examine changes in clustered behaviors over time (11). Classification uncertainty and repeated observations are also frequently handled incompletely.

The Chinese context is especially time-sensitive. During the 2000s, nutrition transition, motorization, urbanization, and changes in school and leisure environments altered both activity and dietary exposure (4, 5, 12, 13). Yet screen measures available in that period largely captured television, video media, and traditional video games rather than the smartphone, social-media, short-video, online-learning, and multi-screen environments that later became common. Analyses of 2004–2011 data can therefore illuminate an earlier phase of China’s behavioral transition, but they cannot describe contemporary screen-based life or current national distributions.

The China Health and Nutrition Survey (CHNS) provides harmonizable measures of physical activity, reported entertainment screen time, three-day dietary intake, measured height and weight, and demographic and contextual characteristics across diverse communities (14–16). Pooling compatible waves increases the analytic sample and permits a common descriptive model, but it also requires direct evaluation of whether the latent structure and profile proportions are stable across time and age. Because some participants contributed more than one observation and profile membership is probabilistic, both repeated participation and classification uncertainty must be incorporated into inference.

Accordingly, this pooled cross-sectional study had two objectives. First, we sought to identify and describe latent profiles defined specifically by total physical activity, reported entertainment screen time, and dietary fat contribution to total energy among school-attending participants aged 6–17 years in the 2004, 2006, 2009, and 2011 CHNS. Second, we examined the concurrent associations between these selected behavioral profiles and measured overweight/obesity. Wave- and age-stratified profile analyses, interaction tests, missing-data analyses, and alternative weight outcomes were treated as robustness and interpretive extensions of these two objectives rather than as separate research aims.

## 2 Materials and methods

### 2.1 Study design and data source

This study used publicly available data from the China Health and Nutrition Survey (CHNS) and was designed as a pooled cross-sectional analysis of person-wave observations. The CHNS is an ongoing, population-based, open-cohort study conducted collaboratively by the National Institute for Nutrition and Health at the Chinese Center for Disease Control and Prevention and the Carolina Population Center at the University of North Carolina at Chapel Hill. The survey was established to examine how social, economic, and policy change in China is related to nutrition, health behaviors, and health outcomes (14, 15).

CHNS sampling used a multistage, random-cluster design. Counties were stratified by income within participating provinces, after which counties, cities, villages, townships, and urban or suburban neighborhoods were selected through staged sampling procedures. The geographic composition of the survey evolved over time, and three large municipalities were added in 2011 (14–16). The present analysis used the 2004, 2006, 2009, and 2011 survey waves because these were the waves in which the child-specific physical-activity, recreational screen-time, dietary, and anthropometric measures required for the three-indicator profile model could be harmonized using consistent analytic definitions. Later survey waves were not included because changes in the content, availability, and comparability of the child activity and recreational screen-time items prevented construction of the same primary indicators across all waves.

Household- and individual-level CHNS master files were used. The principal source files were the survey roster, master identification, education, physical-activity, physical-examination, individual dietary-intake, household-income, and urbanicity files. CHNS longitudinal master files standardize variable names and formats across waves where the underlying survey items are comparable (16). Individual records were linked by participant identifier and survey wave (IDIND and WAVE), household records by household identifier and wave (HHID and WAVE), and community-level constructed variables by community identifier and wave (COMMID and WAVE).

The CHNS does not provide a single set of individual-level sampling weights that can be applied consistently across the analytic waves used here. Accordingly, analyses were unweighted and should not be interpreted as nationally representative prevalence estimates. Survey wave, province, urban residence, household income, and a multidimensional urbanicity index were included to account for major temporal and contextual differences within the observed sample. Reporting was developed with reference to the STROBE recommendations for cross-sectional studies (17).

### 2.2 Study population and analytic samples

The eligible population comprised children and adolescents aged 6–17 years who participated in the 2004, 2006, 2009, or 2011 CHNS and were recorded as currently attending school. The upper age limit was selected to maintain a consistent child/adolescent questionnaire framework below age 18, while the lower age limit aligned with the availability of the relevant school, activity, and sedentary-behavior measures. Restriction to current students ensured that school-based physical activity, homework, and out-of-school reading had a comparable substantive meaning across observations.

Records were excluded from the primary latent profile analysis if any of the three profile indicators—total physical activity, common entertainment screen time, or fat energy share—was missing or invalid, or if a valid body mass index (BMI) and weight-status classification could not be derived. The primary profile-analysis sample therefore contained 4,112 person-wave observations. Fully adjusted association analyses used 4,068 observations with complete values for age, sex, survey wave, urban residence, province, household-income tertile, urbanicity index, and total energy intake. Missingness was quantified separately for each variable and survey wave, and the sequential participant flow was reported because exclusion counts depend on the order in which criteria are applied. Profile estimation was also repeated in 4,441 observations with complete behavioral indicators irrespective of BMI availability.

The analytic unit was the person-wave observation. Thus, a participant could contribute observations from more than one survey wave. Dependence among repeated observations from the same participant was addressed in all association models using participant-clustered robust standard errors. A sensitivity analysis that retained only the most recent eligible observation per participant was used to assess the influence of repeated participation.

### 2.3 Data management, harmonization, and quality control

Raw SAS files were retained without modification. Data were imported, renamed to a common analysis schema, and linked using the prespecified keys described above. Negative values, special missing codes, and values outside a variable’s permissible response range were recoded as missing before any derived variable was created. Structural zero values were assigned only when a corresponding participation item explicitly indicated nonparticipation; time variables that were missing without an explicit nonparticipation response remained missing. This distinction prevented item nonresponse from being reclassified as no activity.

Data-cleaning decisions were specified before the profile and outcome analyses. To prevent a small number of extreme time-use values from disproportionately influencing the mixture model, domain-specific physical-activity totals were capped at prespecified time-budget maxima of 40 h/week for school activity, 60 h/week for leisure activity, and 20 h/week for active travel. The corresponding domain-specific metabolic-equivalent (MET) totals were scaled proportionally when a time cap was applied. These limits were pragmatic time-budget safeguards rather than validated physiological thresholds, so their influence was examined in sensitivity analyses using uncapped and P99-winsorized physical-activity totals. A combined total of common entertainment screen time plus study-related sedentary time greater than 112 h/week was considered incompatible with a plausible weekly time budget and was treated as missing. All repairs, caps, range checks, and logical inconsistencies were retained as audit flags in the cleaned database.

### 2.4 Measures

#### 2.4.1 Physical activity

Physical activity was harmonized across three domains: activity performed at school, organized or informal leisure-time activity outside school, and active travel to and from school. The questionnaire covered martial arts; gymnastics, dance, or acrobatics; track and field or swimming; football, basketball, or tennis; badminton or volleyball; and other sports. School activity was recorded as minutes per school week. Leisure activity was reconstructed from a typical weekday and weekend day and converted to weekly hours as [(weekday minutes x 5) + (weekend-day minutes x 2)]/60. For active travel, daily round-trip minutes of walking or cycling were multiplied by five school days and divided by 60.

Activity time was converted to MET-hours per week using fixed, wave-consistent intensity values. Martial arts was assigned 4.5 METs; gymnastics, dance, or acrobatics 5.0 METs; track and field or swimming 7.5 METs; and football, basketball, or tennis 6.0 METs. Badminton, volleyball, and other sports were assigned 5.0 METs, cycling 4.0 METs, and walking 3.0 METs. The primary physical-activity indicator (PA_TOTAL_MET_H_WK) was the sum of school, leisure, and active-travel MET-hours per week. Household chores and domestic activity were not included because their measurement and conceptual comparability differed from the three movement domains retained for profile analysis. The construction strategy was informed by previous domain-specific analyses of physical activity in CHNS school-aged participants (12, 13).

#### 2.4.2 Sedentary behavior

The primary sedentary indicator was common entertainment screen time, defined as the sum of time spent watching television, watching videotapes/VCDs/DVDs, and playing video games. These behaviors were selected because closely comparable items were available in all four analytic waves. For each behavior, weekly time was calculated as [(weekday minutes x 5) + (weekend-day minutes x 2)]/60. The resulting indicator was expressed in hours per week. Computer and internet items were not included in the primary measure because their content and availability changed across waves as digital media use expanded.

Study-related sedentary time was derived separately as the sum of homework and out-of-school reading or writing. This variable and the sum of common entertainment screen time and study-related sedentary time were retained for descriptive and sensitivity analyses but were not used in the primary three-indicator profile model.

#### 2.4.3 Dietary intake

The CHNS dietary assessment was based on individual 24-h recalls collected on three consecutive days, with complementary household food-inventory information used by the survey to support dietary measurement (14–16). The present study used the CHNS constructed file containing three-day mean daily energy and macronutrient intakes. Total energy intake was considered implausible if it was below 500 or above 5,000 kcal/day. Carbohydrate, fat, and protein intakes were required to be nonnegative and simultaneously available.

Macronutrient energy shares were calculated using standard energy-conversion factors. Carbohydrate energy share was (carbohydrate grams x 4/total kilocalories) x 100, fat energy share was (fat grams x 9/total kilocalories) x 100, and protein energy share was (protein grams x 4/total kilocalories) x 100. The sum of the three shares was required to be between 70 and 130%; observations outside this broad accounting range were treated as invalid for macronutrient-composition analyses. Fat energy share was selected as the primary dietary indicator because it could be constructed consistently across all four waves and captured a major dimension of dietary change during China’s nutrition transition. Total energy intake was retained as a covariate in the fully adjusted association model.

#### 2.4.4 Anthropometry and overweight/obesity

Height and weight were measured during the CHNS physical examination using standardized survey procedures (14–16). Height values outside 80–200 cm and weight values outside 10–150 kg were treated as implausible. BMI was recalculated as weight in kilograms divided by height in meters squared; values below 10 or above 40 kg/m2 were treated as invalid.

Weight status was classified using the Chinese health-industry standard WS/T 586–2018, which provides sex- and age-specific BMI thresholds for overweight and obesity among school-aged children and adolescents aged 6–18 years (18). Because the public-use source file provided age in completed integer years, the x.0-year threshold corresponding to each reported age was applied. The primary binary outcome contrasted overweight/obesity with a combined normal/underweight reference category. For outcome sensitivity analyses, weight status was modeled as a three-category outcome—normal/underweight, overweight, and obesity—and BMI was analyzed continuously. An approximate WHO 2007 BMI-for-age z score was also calculated from sex-specific monthly LMS parameters (19). Exact age in months was unavailable; therefore, the midpoint of each completed-age year was used for the main z-score sensitivity analysis, with the lower and upper boundaries of the completed-age year examined separately. This approximate z score was treated only as a sensitivity outcome, not as a replacement for the primary Chinese-standard classification.

#### 2.4.5 Covariates

Covariates were selected *a priori* to capture demographic, temporal, socioeconomic, and contextual differences that could be related to both behavioral patterns and weight status. These included continuous age; sex (male or female); survey wave (2004, 2006, 2009, or 2011); urban versus rural residence; province; household per-capita income; the CHNS urbanicity index; and total energy intake. Household per-capita income was taken from the consumer-price-index-adjusted constructed income file and categorized into wave-specific tertiles to reduce the influence of secular changes in monetary scale. The urbanicity index is a multidimensional community-development measure that reflects features such as population density, economic activity, markets, transportation, communications, housing, education, and health infrastructure (20).

For modeling, age was mean-centered, the urbanicity index was standardized to a z score, and total energy intake was expressed per 1,000 kcal/day and centered. Survey wave, income tertile, and province were represented by indicator variables. Province code 21 was used as the reference category because it was present in all four waves. Sex, residence, and age group (6–11 vs. 12–17 years) were additionally used as prespecified stratification and interaction variables.

### 2.5 Identification of selected behavioral profiles

Profiles were identified using finite Gaussian mixture models with three continuous indicators: total physical activity (MET-h/week), common entertainment screen time (h/week), and fat energy share (%). Physical activity and screen time were transformed using log(1 + x) to reduce right skew; fat energy share was not transformed. All indicators were standardized using the pooled primary profile-analysis sample. The term profile denotes a statistical summary of indicator heterogeneity and not a naturally occurring or sharply separated population type.

The prespecified primary analysis estimated one- through six-profile models with class-specific diagonal covariance matrices, 300 random initializations for two- through six-profile solutions, a maximum of 2,000 iterations, covariance regularization of 1 × 10–5, and random seed 20,260,712. Candidate solutions were evaluated using AIC, BIC, sample-size-adjusted BIC, entropy, average posterior probabilities, smallest-profile size, convergence, replication across starts, and substantive coherence. The five-profile solution was retained as the primary descriptive working solution because it minimized BIC under the prespecified covariance model, avoided a very small profile, and remained interpretable. BMI and weight status were not used to select, order, or name profiles (21, 22).

Classification quality was evaluated using profile-specific posterior probabilities and the proportion of observations with a maximum posterior probability below 0.70. Stability was examined using participant-level nonparametric bootstrap samples; re-estimated profiles were aligned to the reference centers by minimum-distance matching and compared using the adjusted Rand index (ARI). Because classification was moderate rather than definitive, posterior probabilities were retained for downstream pseudo-class analyses.

To evaluate whether pooling survey waves imposed an unsupported common structure, one- through six-profile models were estimated separately for 2004, 2006, 2009, and 2011 and for children aged 6–11 years and adolescents aged 12–17 years. Group-specific indicators were standardized using the pooled-sample transformation parameters. For comparability, fixed five-profile solutions were aligned to the pooled reference centers, and group-specific BIC-preferred profile counts, center similarity, composition, posterior classification quality, and bootstrap ARI were reported. These analyses were treated as structural sensitivity analyses rather than formal longitudinal measurement-invariance tests.

Model-specification sensitivity analyses compared class-specific diagonal, class-specific full, shared full, and spherical covariance structures; raw, Yeo-Johnson, and rank-normal indicator transformations; and a two-part mixture that represented the concentrated mass at zero entertainment screen time separately from positive screen-time values. BIC was compared only within the same model and transformation family. Auxiliary one- through six-profile models were also estimated in Mplus version 9.1 Demo with indicator variances constrained equal across profiles, using staged random starts, TECH11, and TECH14 with 500 successful bootstrap draws. Because this covariance parameterization differed from the primary model, the Mplus results were treated as sensitivity evidence rather than direct confirmation of the primary solution.

### 2.6 Descriptive analyses

Continuous variables were summarized using means and standard deviations or medians and interquartile ranges, as appropriate; categorical variables were summarized using counts and percentages. Participant characteristics were described by weight status in the common regression sample. Variable-specific and wave-specific missingness, sequential exclusions, and characteristics of records included in and excluded from the primary profile analysis were reported. Standardized mean differences were used descriptively, and a cluster-robust logistic model of inclusion was fitted using fully observed demographic and contextual variables.

Profile-specific descriptive means, proportions, and overweight/obesity prevalence were estimated by weighting each observation by its posterior probability of membership in the corresponding profile. For posterior-weighted prevalence estimates, 95% confidence intervals were obtained from 500 participant-level bootstrap samples so that all observations from the same participant were resampled together.

### 2.7 Concurrent association between selected behavioral profiles and overweight/obesity

The primary association between profile membership and binary overweight/obesity was estimated using logistic regression, with P2 (low-to-moderate activity/lower fat) as the outcome-independent reference profile. Classification uncertainty was propagated using 100 posterior-probability pseudo-class draws. In each draw, a profile was sampled for every observation from its five posterior membership probabilities; regression coefficients and covariance matrices were then pooled using Rubin-type combination rules (23, 24). Robust covariance estimates were clustered by participant identifier to account for repeated person-wave observations (31).

Four sequential binary models were estimated in the same common complete-case sample. Model 1 included profile only; Model 2 added age, sex, and survey wave; Model 3 added urban residence, wave-specific household-income tertile, and standardized urbanicity; and Model 4 additionally included province fixed effects and centered total energy intake and was designated the fully adjusted model. Overall profile associations were assessed using pooled four-degree-of-freedom Wald tests. Secondary outcome models used the fully adjusted covariate set: multinomial logistic regression separately contrasted overweight and obesity with normal/underweight, and linear regression analyzed continuous BMI and approximate WHO BMI-for-age z score. Multinomial results are reported as relative risk ratios (RRRs) and continuous results as adjusted mean differences.

### 2.8 Stratified, interaction, and sensitivity analyses

Prespecified stratified analyses were conducted by sex, urban–rural residence, and age group (6–11 vs. 12–17 years). The fully adjusted covariate set was used within each stratum, omitting the stratifying variable where appropriate. Each modifier was evaluated in a separate interaction model that added the full set of profile-by-modifier terms; the interaction *p*-value was obtained from a four-degree-of-freedom joint Wald test. All stratified and interaction models used 100 pseudo-class draws and participant-clustered robust covariance estimates.

Sensitivity analyses addressed classification, repeated observations, profile specification, missing data, selection, and outcome definition. Association models used modal assignment, restricted the sample to maximum posterior probability ≥0.70, retained only the latest eligible wave per participant, restricted age to 7–17 years, used obesity alone, omitted province fixed effects, and used participant-, community-, or two-way-clustered covariance estimates. Profile enumeration was repeated in the behavior-complete sample and under alternative physical-activity capping, covariance, transformation, and screen-zero specifications. Missing household-income tertile values within the primary profile sample were handled in a 20-imputation sensitivity analysis. Stabilized inverse-probability-of-inclusion weights were estimated from fully observed demographic and contextual variables and combined with the imputed analyses; weight distributions, effective sample size, covariate balance, and changes in profile proportions were examined. The multinomial weight-status and continuous BMI/z-score models described above provided outcome-form sensitivity analyses.

### 2.9 Statistical software, reproducibility, and significance threshold

Data cleaning, variable construction, primary model estimation, sensitivity analyses, and figure generation were performed in Python 3.13.5; the auxiliary equal-variance class-enumeration analysis was performed in Mplus version 9.1 Demo. Core Python packages included NumPy 2.3.5, pandas 2.2.3, SciPy 1.17.0, scikit-learn 1.8.0, and statsmodels 0.14.6 (32, 33). The complete workflow was versioned by step, and derived databases, audit tables, and code files were assigned SHA-256 checksums. Hypothesis tests were two-sided with *p* < 0.05. Profile-specific comparisons were interpreted as secondary to global tests and were not multiplicity-adjusted.

### 2.10 Ethical considerations

The original CHNS protocols were reviewed and approved by the institutional review boards at the University of North Carolina at Chapel Hill and the National Institute of Nutrition and Food Safety, Chinese Center for Disease Control and Prevention (now the National Institute for Nutrition and Health). Written informed consent was obtained from participants and/or their parents or legal guardians, as appropriate (14, 15). The present study used publicly available, de-identified secondary data and involved no direct participant contact.

## 3 Results

### 3.1 Sample selection and participant characteristics

The detailed sample-selection process is shown in Figure 1. The CHNS master file contained 143,564 person-wave records; 52,071 were from the four analytic waves, 6,062 were aged 6–17 years, and 5,492 were currently attending school. Sequentially, 5,046 had valid BMI and weight-status classification, 4,327 had complete total physical activity, 4,234 also had complete entertainment screen time, and 4,112 additionally had complete fat-energy share and formed the primary profile-analysis sample. Forty-four observations lacked household-income tertile, leaving 4,068 observations from 2,757 unique participants for the fully adjusted complete-case analysis.

**Figure 1:**
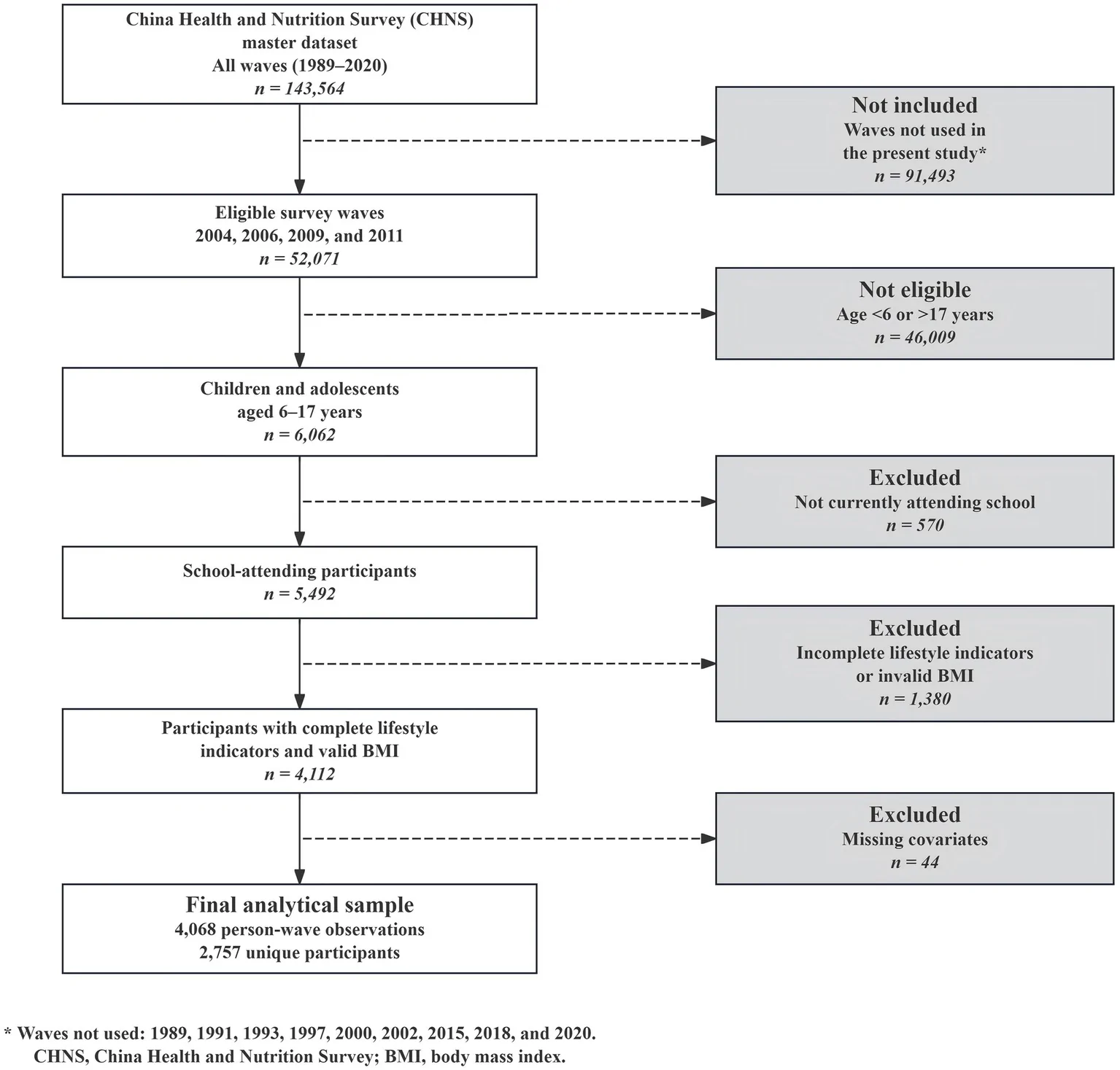
Selection of the analytic samples. CHNS, China Health and Nutrition Survey; BMI, body mass index. The figure summarizes sequential selection from the CHNS master dataset to the primary profile-analysis sample (n = 4,112) and the fully adjusted complete-case sample (n = 4,068; 2,757 unique participants). Detailed variable-specific missingness by survey wave is reported in Supplementary Table S17.

In the final regression sample, mean age was 11.1 years (SD 3.1), 51.6% were male, and 31.2% lived in urban areas. Weight status comprised 3,352 normal/underweight observations, 416 overweight observations, and 300 obesity observations; 716 observations (17.6%) were overweight or obese in the primary binary analysis. The 2004, 2006, 2009, and 2011 waves contributed 25.3, 21.2, 22.3, and 31.1% of observations, respectively (Table 1).

**Table 1:** Characteristics of the complete regression sample by weight status.

Characteristic	Overall (*n* = 4,068)	Normal/underweight (*n* = 3,352)	Overweight/obese (*n* = 716)	*p*-value
Age, years, mean (SD)	11.1 (3.1)	11.3 (3.1)	10.2 (3.0)	<0.001
Sex, *n* (%)				<0.001
Male	2098 (51.6)	1,661 (49.6)	437 (61.0)	
Female	1970 (48.4)	1,691 (50.4)	279 (39.0)	
Age group, *n* (%)				<0.001
6–11 years	2,235 (54.9)	1750 (52.2)	485 (67.7)	
12–17 years	1833 (45.1)	1,602 (47.8)	231 (32.3)	
Residence, *n* (%)				<0.001
Rural	2,799 (68.8)	2,376 (70.9)	423 (59.1)	
Urban	1,269 (31.2)	976 (29.1)	293 (40.9)	
Household income tertile, *n* (%)				<0.001
Low	1,359 (33.4)	1,164 (34.7)	195 (27.2)	
Middle	1,349 (33.2)	1,132 (33.8)	217 (30.3)	
High	1,360 (33.4)	1,056 (31.5)	304 (42.5)	
Survey year, *n* (%)				<0.001
2004	1,030 (25.3)	883 (26.3)	147 (20.5)	
2006	864 (21.2)	739 (22.0)	125 (17.5)	
2009	908 (22.3)	752 (22.4)	156 (21.8)	
2011	1,266 (31.1)	978 (29.2)	288 (40.2)	
Urbanicity index, mean (SD)	64.9 (20.0)	63.9 (19.9)	69.7 (19.7)	<0.001
Energy intake, kcal/day, median (IQR)	1619.1 (1278.6–2049.1)	1616.9 (1284.4–2040.9)	1648.4 (1248.8–2098.7)	0.583
Total physical activity, MET-h/week, median (IQR)	20.4 (10.4–42.9)	20.6 (10.8–43.0)	20.0 (9.2–42.5)	0.401
Entertainment screen time, h/week, median (IQR)	9.0 (5.3–15.0)	9.0 (5.0–15.0)	9.0 (5.9–14.0)	0.717
Fat energy share, %, mean (SD)	30.4 (11.3)	30.1 (11.2)	32.0 (11.4)	<0.001

Values are mean (SD), median (IQR), or *n* (%). *p*-values were obtained from univariable logistic models with participant-clustered robust standard errors; categorical variables were evaluated using joint Wald tests. IQR, interquartile range; MET-h, metabolic equivalent-hours.

Among the 5,492 eligible school-attending records, missingness was highest for physical activity (14.42%), followed by BMI (8.12%), entertainment screen time (5.32%), fat-energy share (3.82%), and household-income tertile (1.58%). Missingness varied by wave; physical-activity missingness was 20.45% in 2004 and 10.32% in 2011. Included records were more strongly represented in 2011 than excluded records (31.23% vs. 19.28%; SMD, 0.278). The inclusion model had an area under the receiver-operating-characteristic curve of 0.676, indicating that selection was partly predictable from observed characteristics (Supplementary Tables S17, S18).

### 3.2 Distribution of selected behavioral indicators

Median total physical activity was 20.4 MET-h/week (IQR, 10.4–42.9), median entertainment screen time was 9.0 h/week (IQR, 5.3–15.0), and mean fat-energy share was 30.4% (SD 11.3). Before transformation, physical activity and screen time were right-skewed (skewness 3.07 and 1.78); log(1 + x) reduced skewness to −0.42 and −0.97, respectively. Entertainment screen time equaled zero for 221 observations (5.37%), creating a concentrated mass at the lower boundary that remained after transformation (Supplementary Table S15).

### 3.3 Identification and characterization of selected behavioral profiles

Under the prespecified class-specific diagonal covariance model, BIC was lowest for five profiles (31,879.70) and increased for six profiles (31,887.26) (Table 2). The five-profile solution had a minimum modal profile size of 5.4%, entropy of 0.563, and average maximum posterior probability of 0.708. Across archived and independently rerun bootstrap analyses, mean ARI ranged from 0.725 to 0.805, indicating moderate-to-good resampling stability rather than perfect separation. Nearly half of observations had a maximum posterior probability below 0.70, and profile-specific mean assigned posterior probabilities ranged from 0.641 to approximately 1.000 (Supplementary Table S16).

**Table 2:** Fit statistics for the one- through six-profile latent profile models.

Profiles	Log-likelihood	Free parameters	AIC	BIC	Adjusted BIC	Entropy	Smallest profile, %	Average maximum posterior probability	Selected
1	−17,504.03	6	35,020.05	35,057.98	35,038.92	1.000	100.0	1.000	
2	−17,141.75	13	34,309.49	34,391.67	34,350.37	0.351	18.4	0.804	
3	−15,871.11	20	31,782.23	31,908.66	31,845.11	0.505	5.4	0.709	
4	−15,841.64	27	31,737.28	31,907.97	31,822.17	0.465	5.4	0.678	
5	−15,798.38	34	31,664.77	31,879.70	31,771.67	0.563	5.4	0.708	Yes
6	−15,773.04	41	31,628.07	31,887.26	31,756.98	0.550	5.4	0.674	

AIC, Akaike information criterion; BIC, Bayesian information criterion. The five-profile solution was retained as the prespecified descriptive working solution under the class-specific diagonal covariance model. Alternative covariance, transformation, wave-specific, age-specific, and screen-zero analyses showed that the exact latent partition was specification-sensitive (Supplementary Tables S12–S16).

Re-estimation in 4,441 behavior-complete observations again selected five profiles and was highly concordant with the primary modal assignments (ARI, 0.952). Uncapped and P99-winsorized physical-activity specifications also selected five profiles and produced ARIs of 0.963–0.966. However, model enumeration was sensitive to broader distributional and covariance assumptions: BIC-preferred solutions ranged from three to six profiles across alternative covariance structures and transformations. A two-part screen-zero mixture selected five profiles within that model family but generated a 0.75% profile and had low agreement with the primary solution (ARI, 0.087), so it was not considered a superior replacement (Supplementary Table S15).

In the auxiliary equal-variance Mplus analysis, BIC continued to decrease through six profiles, and the five-versus-six adjusted Lo–Mendell–Rubin and bootstrap likelihood-ratio tests were significant. Both the five- and six-profile solutions nevertheless contained a profile with approximately 0.7% estimated prevalence and 24 modal observations. These results were interpreted as evidence that class enumeration depended on covariance restrictions and the concentrated lower tail of screen time, not as confirmation of six substantively distinct groups (Supplementary Table S12).

The selected five-profile working solution is shown in Figure 2 and Table 3. P1 represented very low activity; P2 low-to-moderate activity/lower fat; P3 no reported entertainment screen time; P4 higher fat; and P5 high activity. All 221 observations assigned to P3 reported zero entertainment screen time, whereas no zero screen-time observations were assigned to the other profiles. P3 therefore describes non-reporting of the entertainment screen behaviors measured in these waves, not minimal total sedentary time. Posterior-weighted profile proportions were 9.4, 33.2, 5.4, 28.6, and 23.4%, respectively.

**Figure 2:**
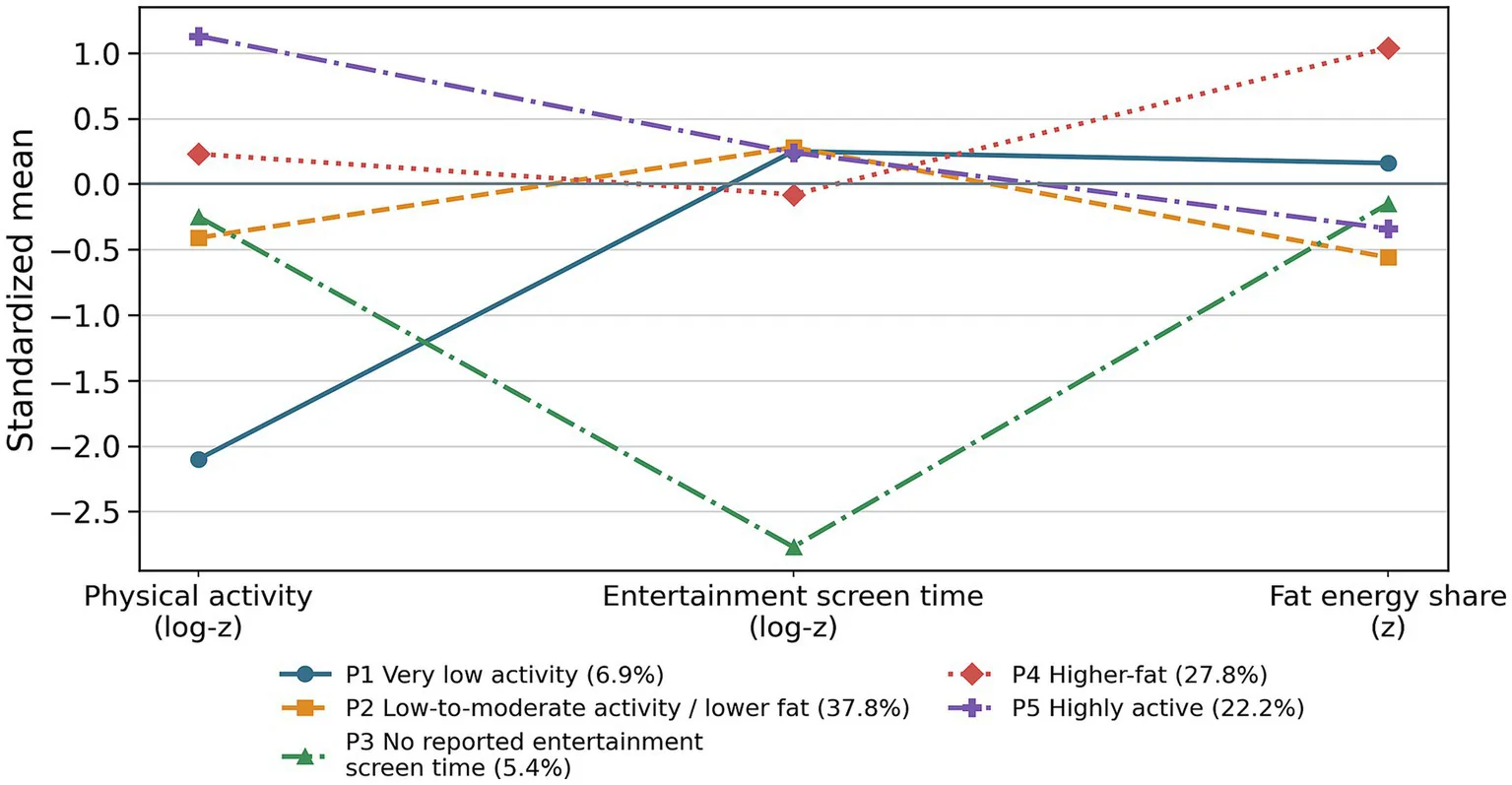
Standardized indicator means for the prespecified five-profile behavioral working solution. Physical activity and entertainment screen time were transformed using log(1 + x) before z-standardization; fat-energy share was standardized without transformation. Percentages are based on modal assignment. P3 comprises observations reporting zero time for the entertainment screen behaviors measured in these waves and should not be interpreted as minimal total sedentary time.

**Table 3:** Characteristics of the selected five behavioral profiles.

Profile	Profile label	Modal *n* (%)	Posterior-weighted proportion, %	Average maximum posterior probability	Physical activity, MET-h/week, weighted mean	Entertainment screen time, h/week, weighted mean	Fat energy share, %, weighted mean	Overweight/obesity, weighted % (95% CI)
P1	Very low activity	282 (6.9)	9.4	0.815	5.2	12.1	31.1	24.4 (20.9–28.1)
P2	Low-to-moderate activity/lower fat	1,556 (37.8)	33.2	0.703	16.0	12.1	25.0	16.4 (14.7–18.2)
P3	No reported entertainment screen time	221 (5.4)	5.4	1.000	27.5	0.0	28.8	15.8 (11.3–20.7)
P4	Higher-fat	1,142 (27.8)	28.6	0.641	37.1	10.1	38.5	18.7 (16.9–20.6)
P5	Highly active	911 (22.2)	23.4	0.697	71.1	11.9	28.4	15.8 (13.9–17.7)

Modal *n* (%) is based on the profile with the highest posterior probability. Weighted means, proportions, and overweight/obesity prevalence incorporate the full posterior probability distribution. CI, confidence interval; MET-h, metabolic equivalent-hours.

Wave-specific model enumeration did not yield an identical number of profiles: BIC favored two profiles in 2004, five in 2006 and 2009, and six in 2011. Fixed five-profile centers were broadly similar to the pooled centers in 2006–2011, whereas replication of the very-low-activity center was weaker in 2004. Under the pooled common-profile representation, composition changed modestly across waves (Cramér’s *V*, 0.095): the higher-fat profile increased from 24.85% in 2004 to 33.98% in 2011, while the low-to-moderate activity/lower-fat profile decreased from 36.51 to 28.70% (Supplementary Tables S13, S14 and Supplementary Figure S5).

### 3.4 Concurrent associations between selected behavioral profiles and weight status

Posterior-probability-weighted overweight/obesity prevalence differed descriptively across profiles (Table 3 and Supplementary Figure S2). The very-low-activity profile had the highest estimated prevalence (24.4%; 95% CI, 20.9–28.1%), followed by the higher-fat profile (18.7%; 95% CI, 16.9–20.6%). Prevalence was 16.4% (95% CI, 14.7–18.2%) in the low-to-moderate activity/lower-fat profile, 15.8% (95% CI, 11.3–20.7%) in the no reported entertainment screen time profile, and 15.8% (95% CI, 13.9–17.7%) in the highly active profile.

In the unadjusted pseudo-class model, the global test for differences across selected behavioral profiles was statistically significant (Wald chi-square = 10.03, 4 df, *p* = 0.040). Relative to the low-to-moderate activity/lower-fat profile, the very-low-activity profile had higher odds of overweight/obesity (OR = 1.64; 95% CI, 1.16–2.34; *p* = 0.006), whereas the other profiles did not differ significantly from the reference profile (Table 4). After adjustment for age, sex, and survey year, the estimate for the very-low-activity profile was attenuated but remained individually significant (OR = 1.46; 95% CI, 1.02–2.09; *p* = 0.039), although the overall profile test was no longer significant (*p* = 0.235). Further adjustment for residence, household-income tertile, and urbanicity reduced the estimate to 1.36 (95% CI, 0.94–1.96; *p* = 0.098). In the fully adjusted model, which additionally included province fixed effects and total energy intake, the association was further attenuated (OR = 1.18; 95% CI, 0.81–1.69; *p* = 0.387). The fully adjusted global profile test was not significant (*p* = 0.806), and none of the other profiles was independently associated with overweight/obesity (Figure 3). The progressive attenuation of the very-low-activity estimate is shown in Supplementary Figure S3.

**Table 4:** Concurrent associations between selected behavioral profiles and overweight/obesity across sequential adjustment models.

Profile	Model 1		Model 2		Model 3		Model 4	
	OR (95% CI)	*P*	OR (95% CI)	*P*	OR (95% CI)	*P*	OR (95% CI)	*P*
P1 Very low activity	1.64 (1.16–2.34)	0.006	1.46 (1.02–2.09)	0.039	1.36 (0.94–1.96)	0.098	1.18 (0.81–1.69)	0.387
P3 No reported entertainment screen time	0.98 (0.65–1.46)	0.909	1.24 (0.82–1.87)	0.310	1.11 (0.73–1.68)	0.621	0.99 (0.64–1.53)	0.969
P4 Higher-fat	1.19 (0.90–1.57)	0.220	1.18 (0.89–1.56)	0.251	0.99 (0.74–1.32)	0.936	0.96 (0.71–1.30)	0.804
P5 Highly active	0.98 (0.74–1.30)	0.879	1.01 (0.76–1.35)	0.937	0.92 (0.69–1.23)	0.573	0.93 (0.69–1.25)	0.621
Global profile test, *p* value	0.040		0.235		0.307		0.806	

OR, odds ratio; CI, confidence interval. The reference profile was P2. Model 1 included profile only; Model 2 added age, sex, and survey wave; Model 3 added residence, household-income tertile, and urbanicity; Model 4 added province fixed effects and total energy intake. Each model reports OR (95% CI) and a separate P value. Estimates were pooled across 100 pseudo-class draws with participant-clustered robust covariance.

**Figure 3:**
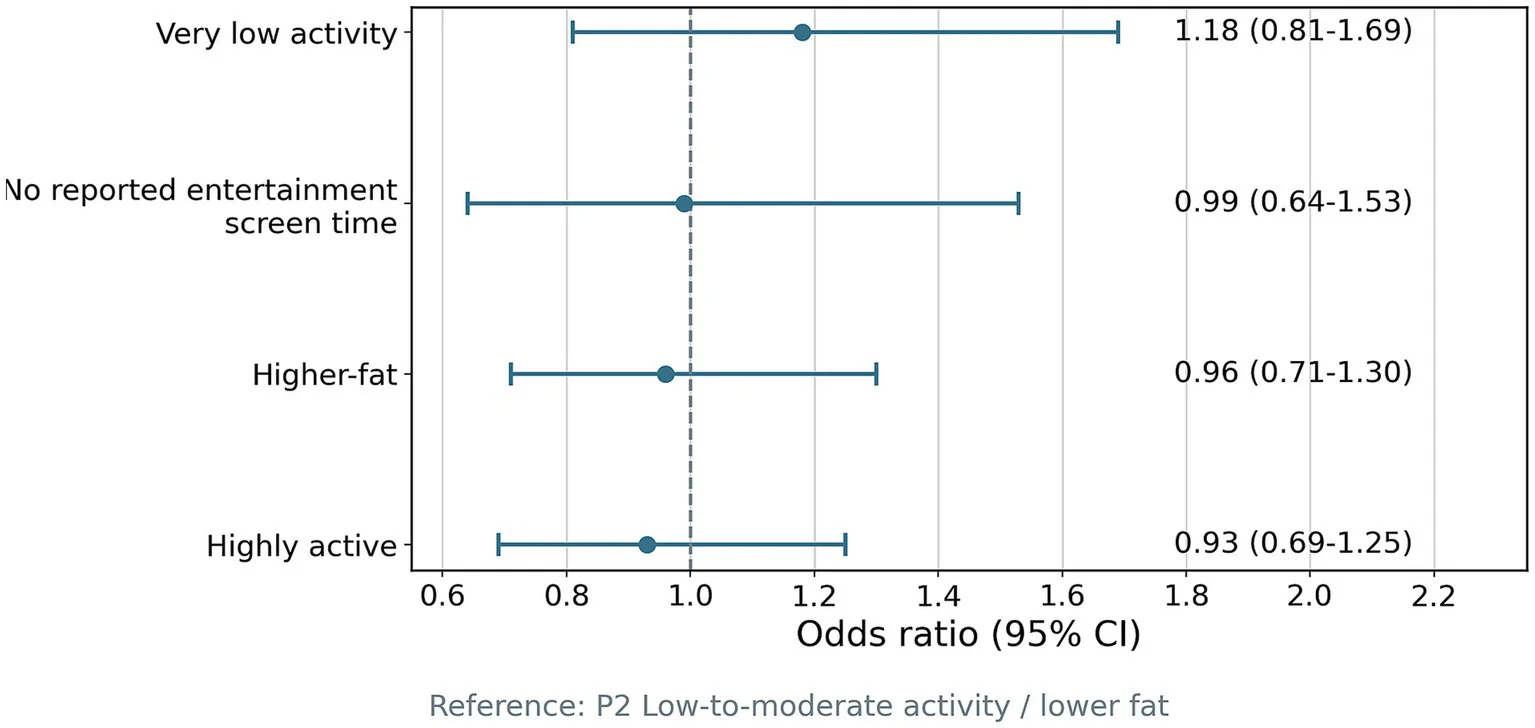
Fully adjusted concurrent associations between the selected behavioral profiles and binary overweight/obesity. Odds ratios and 95% confidence intervals were estimated using 100 pseudo-class draws to account for profile-classification uncertainty and participant-clustered robust standard errors. The reference group was P2, the low-to-moderate activity/lower-fat profile. The fully adjusted model included age, sex, survey year, urban residence, household-income tertile, urbanicity index, province fixed effects, and total energy intake.

Separating overweight and obesity did not reveal outcome-specific associations. In fully adjusted multinomial models, the global profile tests were *p* = 0.935 for overweight and *p* = 0.846 for obesity, and the joint eight-degree-of-freedom test was *p* = 0.980. For P1 versus P2, the RRR was 1.11 (95% CI, 0.69–1.79) for overweight and 1.25 (95% CI, 0.75–2.07) for obesity. Continuous outcomes were also null: the global *p* value was 0.600 for BMI and 0.797 for midpoint-assigned approximate WHO BMI-for-age *z* score; lower- and upper-bound completed-age assumptions produced global *p* values of 0.854 and 0.733 (Supplementary Tables S20, S21).

### 3.5 Stratified and interaction analyses

Age-stratified LPA also indicated structural sensitivity. BIC favored four profiles among children aged 6–11 years and six profiles among adolescents aged 12–17 years. When fixed five-profile solutions were aligned to the pooled reference, center patterns remained broadly recognizable, but profile composition differed (Cramér’s *V*, 0.211); the posterior-weighted highly active proportion was 19.42% among children and 28.27% among adolescents. These results support reporting a common pooled descriptive framework while avoiding a claim of age-invariant profile structure (Supplementary Tables S13, S14 and Supplementary Figure S6).

Fully adjusted associations were examined separately by sex, residence, and age group (Table 5 and Supplementary Table S4). Estimates for the very-low-activity profile were positive but imprecise among males (OR = 1.20; 95% CI, 0.73–1.99), females (OR = 1.14; 95% CI, 0.67–1.95), and rural participants (OR = 1.29; 95% CI, 0.82–2.05). The corresponding estimates were 1.14 (95% CI, 0.74–1.76) among children aged 6–11 years and 1.37 (95% CI, 0.65–2.88) among adolescents aged 12–17 years. The estimate among urban participants was close to the null (OR = 0.96; 95% CI, 0.51–1.79). No profile-specific or global test within a stratum was statistically significant. Joint interaction tests did not provide evidence that associations differed by sex (*p* = 0.585), urban–rural residence (*p* = 0.855), or age group (*p* = 0.545) (Supplementary Table S5). The wide confidence intervals nevertheless leave modest subgroup differences unresolved.

**Table 5:** Fully adjusted concurrent association of the very-low-activity profile with overweight/obesity across population strata.

Stratum	*N*	Events	OR (95% CI)	*P*-value	Global profile *P*-value	Interaction *P*-value
Male	2098	437	1.20 (0.73–1.99)	0.474	0.838	0.585
Female	1970	279	1.14 (0.67–1.95)	0.617	0.809	0.585
Rural	2,799	423	1.29 (0.82–2.05)	0.275	0.758	0.855
Urban	1,269	293	0.96 (0.51–1.79)	0.889	0.867	
6–11 years	2,235	485	1.14 (0.74–1.76)	0.545	0.853	0.545
12–17 years	1833	231	1.37 (0.65–2.88)	0.403	0.716	

The reference profile was P2. Each modifier—sex, residence, or age group—was evaluated in a separate interaction model containing all profile-by-modifier terms. Stratum-specific models used the fully adjusted covariate set, excluding the stratifying variable where appropriate. Interaction *p*-values are four-degree-of-freedom joint Wald tests from the corresponding separate interaction model.

### 3.6 Sensitivity analyses

The association conclusion remained unchanged across the prespecified classification, repeated-observation, age-range, obesity-only, and covariate sensitivities (Supplementary Table S6). Profile-specification analyses provided a more qualified result: the selected number and membership boundaries varied under alternative covariance, transformation, and screen-zero assumptions, although the prespecified five-profile working solution remained reproducible in the behavior-complete and alternative physical-activity analyses (Supplementary Tables S9, S11, S15, S16).

Selection and missing-data analyses did not materially change the descriptive profile distribution or the fully adjusted null association. Stabilized inclusion weights ranged from 0.800 to 1.762 and yielded an effective sample size of approximately 4,003; the largest absolute standardized mean difference decreased from 0.070 before weighting to 0.005 after weighting. The maximum change in any posterior-weighted profile proportion was 0.28 percentage points. Multiple imputation of the 44 missing income-tertile values produced a P1-versus-P2 OR of 1.18 (95% CI, 0.82–1.71; global *p* = 0.752), and the combined IPW-plus-imputation analysis produced an OR of 1.17 (95% CI, 0.80–1.71; global *p* = 0.822) (Supplementary Tables S18, S19).

Community- and two-way-clustered covariance estimates likewise left the fully adjusted binary association non-significant. Together, the sensitivity analyses support the primary null association conclusion while showing that the detailed latent partition itself is model-dependent and should not be interpreted as a fixed population taxonomy.

## 4 Discussion

### 4.1 Principal findings

In the pooled 2004–2011 CHNS analytic sample, a prespecified five-profile model summarized heterogeneous combinations of total physical activity, reported entertainment screen time, and fat-energy share. The profiles were mixed rather than forming a simple favorable-unfavorable continuum: high activity coexisted with average fat intake, higher fat intake appeared across moderate activity levels, and one small profile was defined by reporting zero time for the entertainment-screen items available in these waves.

The five-profile solution was useful as a common descriptive working representation, but it was not structurally invariant. The BIC-preferred number of profiles varied across survey waves, age groups, covariance structures, transformations, and the two-part screen-time model. Classification separation was moderate, with almost half of observations below a maximum posterior probability of 0.70. These results require a conditional interpretation: the profiles summarize variation in the pooled data, but they are not sharply separated or naturally occurring population types.

The very-low-activity profile had the highest estimated prevalence of overweight/obesity, but the difference was attenuated after demographic, temporal, socioeconomic, geographic, and energy-intake adjustment. The fully adjusted global test was non-significant, and the same conclusion was obtained when overweight and obesity were modeled separately, when BMI and approximate WHO BMI-for-age z score were treated as continuous outcomes, and after missing-data and selection weighting. Thus, the data support behavioral heterogeneity, but not independent discrimination of concurrent weight status by profile.

### 4.2 Interpreting historical behavioral heterogeneity

Mixed behavioral configurations are consistent with earlier reviews showing that children and adolescents frequently combine favorable and unfavorable behaviors rather than occupying uniformly healthy or unhealthy categories (6–8). This is a central advantage of a person-centered description: it reveals combinations that single-exposure models do not show. It is not, however, evidence that a particular number of latent groups exists independently of the chosen indicators and model.

The observed combinations should be interpreted within China’s 2004–2011 social and nutrition transition. Economic growth, urbanization, motorization, changing food availability, and shifts in school and leisure environments did not necessarily alter activity, sedentary behavior, and diet in parallel (5, 25). The increasing proportion of the higher-fat profile and the declining proportion of the lower-fat reference profile across waves are compatible with historical change, but the varying wave-specific solutions show that pooling also compresses temporal heterogeneity.

The no-reported-entertainment-screen-time profile illustrates the limits of measurement-based naming. It contained the 221 observations reporting zero television, video-media, and video-game time and was therefore separated almost mechanically by the Gaussian model. The label does not imply an absence of homework sitting, reading, computer or internet use, other sedentary behavior, or unmeasured digital media. It should be read literally as a response pattern for the entertainment-screen items available in those survey waves.

More broadly, the three indicators do not represent the full spectrum of lifestyle. Total physical activity and reported entertainment screen time were questionnaire-based; the screen measure was not total sedentary time; fat-energy share was not overall diet quality; and sleep was unavailable. For this reason, the revised terminology refers to selected behavioral profiles or to the three measured domains directly, rather than treating the profiles as comprehensive lifestyle classifications.

### 4.3 Concurrent associations with weight status

The adjusted null finding is consistent with an evidence base in which profile-adiposity associations have varied across settings and methods (6, 9, 10, 34). Studies differ in age range, behavior selection, class enumeration, weight-status definition, and control for socioeconomic and environmental context. A profile that appears unfavorable in one indicator may contain compensating or poorly measured behaviors in another, reducing its ability to distinguish BMI-defined weight status.

Some Chinese adolescent studies have reported class differences in overweight, obesity, or cardiometabolic indicators (26, 27). Those analyses included broader sets of diet, sleep, health-consciousness, or school-related variables and focused primarily on older adolescents. The present analysis used only three harmonizable domains across a wider age range and propagated classification uncertainty. Differences in indicator breadth and population composition therefore limit direct comparison of class labels and effect estimates (35, 36).

Prospective evidence also suggests that duration and temporal ordering matter. Behavioral clusters have predicted later adiposity in some cohorts, while other studies have found weak or inconsistent tracking and associations (28–30). In the present pooled cross-sectional design, behaviors and weight status were measured concurrently. Participant-clustered standard errors address repeated observations but do not establish that the behavioral configuration preceded adiposity or exclude weight-related changes in activity, screen use, or diet (37, 38).

The sequential attenuation should therefore not be interpreted as identifying causal mechanisms. Survey wave, age, sex, residence, income, urbanicity, province, and energy intake may represent confounders, mediators, contextual pathways, or proxies for unmeasured conditions. The models estimate concurrent adjusted associations; they do not show that changing profile membership would change body weight.

The lack of interaction evidence by sex, residence, or age group likewise does not prove uniformity. Confidence intervals were wide in several strata, and the age-stratified LPA showed different BIC-preferred structures and profile proportions. The subgroup results are best interpreted as an absence of clearly detected interaction in this sample, not as confirmation that behavioral patterns operate identically across developmental and social contexts.

BMI also captures accumulated growth and energy balance, whereas the behavioral indicators reflect relatively short reporting windows. Three-day dietary recalls and a reported week of activity and screen behavior may not represent long-term exposure sufficiently to differentiate current BMI. This temporal mismatch, together with self-report error and mixed within-profile behaviors, provides a plausible explanation for descriptive prevalence differences that did not persist after adjustment.

### 4.4 Implications for surveillance and future research

The findings do not validate the selected profiles as obesity-risk strata and do not establish that profile-based interventions would outperform strategies directed at established behavioral and environmental targets. The defensible contribution is descriptive: the analysis shows that three behaviors coexisted in mixed configurations during an earlier phase of China’s transition and that those configurations did not independently distinguish concurrent weight status after adjustment.

For surveillance, person-centered analyses can complement behavior-specific indicators by showing how activity, sedentary media use, and dietary exposures combine. Such analyses should retain the measured-domain labels, report classification uncertainty, and avoid presenting unweighted profile proportions as national prevalence. They should also be repeated in contemporary datasets rather than assuming that profiles derived before widespread smartphone and multi-screen use remain applicable.

Contemporary studies should incorporate accelerometry or other device-based movement measures, smartphone and multi-screen exposure, sleep, food groups and diet-quality indices, school and neighborhood environments, parental characteristics, and repeated anthropometry. These additions would test whether broader and better-measured behavioral configurations provide information beyond their individual components.

Longitudinal and transition-oriented designs are especially important. Latent transition or growth-mixture models could examine whether children move between configurations, whether cumulative duration in a configuration matters, and whether behavioral changes precede changes in adiposity. External replication across regions and nationally representative samples is needed before profile distributions or profile-based applications are generalized.

### 4.5 Strengths and limitations

Methodological strengths include measured height and weight, a person-centered analytical framework, explicit comparison of multiple profile-enumeration criteria, propagation of profile-classification uncertainty, participant-clustered inference for repeated observations, and extensive sensitivity analyses addressing wave and age structure, covariance and distribution assumptions, high activity values, missing data, sample selection, clustering level, and alternative weight outcomes.

Several limitations are central to interpretation. First, the analysis was pooled cross-sectional. Concurrent measurement prevents temporal ordering, permits reverse causation, and means that repeated participation cannot be interpreted as a longitudinal profile-transition design.

Second, the data represent 2004–2011. They are valuable for describing an earlier phase of China’s nutrition and activity transition but do not capture later smartphone, social-media, online-learning, food-delivery, and multi-screen environments. The findings cannot be used as direct evidence about current Chinese children and adolescents or current school and community interventions.

Third, the profiles were based on only three selected indicators. Questionnaire-based physical activity and screen time are susceptible to recall and reporting error; entertainment screen time is not total sedentary behavior; fat-energy share does not measure overall dietary quality; and sleep was unavailable. P3 was defined by a point mass at zero, and the exact profile number and membership boundaries varied across assumptions.

Fourth, classification separation was moderate. Although pseudo-class sampling propagated membership uncertainty into association estimates, it cannot turn overlapping latent distributions into sharply separated groups. Labels are descriptive summaries of indicator means and should not be interpreted as fixed identities.

Fifth, analyses were unweighted because a common individual sampling weight was unavailable across the pooled waves. CHNS is not nationally representative, its geographic composition changed, and the results apply only to the analytic sample. Complete-case inclusion differed by wave. Inverse-probability weighting and multiple imputation did not materially alter the findings, but residual selection related to unobserved variables cannot be excluded.

Finally, residual confounding remains possible because pubertal stage, parental BMI, sleep, detailed food environments, school characteristics, and psychosocial factors were unavailable. BMI-for-age z scores were approximate because exact age in months was not present in the analytic file, and the short behavioral assessment windows may not represent habitual exposure.

### 4.6 Conclusion

Among school-attending observations in the 2004–2011 CHNS analytic sample, a prespecified five-profile model provided a parsimonious but model-dependent summary of total physical activity, reported entertainment screen time, and fat-energy share. The exact latent structure varied by wave, age group, and modeling assumptions, and the profiles did not independently distinguish concurrent overweight, obesity, BMI, or approximate BMI-for-age z score after adjustment. These findings should be interpreted as historical, sample-specific evidence of behavioral heterogeneity, not as current national prevalence estimates or validation of profile-based interventions. Contemporary longitudinal studies with broader and more objective behavioral measurement are needed.

## Data Availability

The original contributions presented in the study are included in the article/Supplementary material, further inquiries can be directed to the corresponding author.
