# Supplementary material for: Physical activity, entertainment screen time, and dietary fat profiles and concurrent weight status among school-attending Chinese children and adolescents: CHNS 2004–2011
Source: Front Public Health. 2026 Sep 28;14:1945546. doi: 10.3389/fpubh.2026.1945546 (PMC13654989; doi:10.3389/fpubh.2026.1945546)

Supplementary Material

Physical Activity, Entertainment Screen Time, and Dietary Fat Profiles and Concurrent Weight Status among School-Attending Chinese Children and Adolescents: CHNS 2004-2011

Supplementary tables

Supplementary Table S1. Construction of study variables.

Construct	Source variable(s)	Operationalization	Unit/coding
Study population	AGE; A13	Age 6-17 years and currently attending school	Eligibility criterion
Physical activity	School, leisure, and active travel activity items	Activity-specific time multiplied by fixed MET values and summed	MET-h/week
Entertainment screen time	Television, video/VCD/DVD, and video-game time	Weekday and weekend time converted to weekly hours and summed	h/week
Fat energy share	D3FAT; D3KCAL	9 x fat grams / total energy x 100	% of total energy
Total energy intake	D3KCAL	Three-day average intake; values <500 or >5,000 kcal/day flagged as implausible	kcal/day
BMI	Measured height and weight	Weight (kg) / height (m)^2	kg/m^2
Overweight/obesity	BMI; sex; age	Sex- and age-specific WS/T 586-2018 BMI thresholds	Binary
Urban residence	URBAN	Official CHNS urban/rural classification	Binary
Household income	HH_INCOME_PC_CPI	CPI-adjusted per-capita household income, categorized into wave-specific tertiles	Low/middle/high
Urbanicity	URBANICITY_INDEX	CHNS community urbanicity index	Continuous
selected behavioral profiles	PA, screen time, fat energy share	Log1p transformation for PA and screen time, z-standardization, continuous LPA	Five profiles

Supplementary Table S2. Missingness of key variables by survey wave.

Survey year	Variable	Eligible N	Non-missing N	Missing N	Missing, %
2004	INCOME_TERTILE	1555	1535	20	1.29
2004	BMI	1555	1400	155	9.97
2004	FAT_PCT_ENERGY	1555	1474	81	5.21
2004	PA_TOTAL_MET_H_WK	1555	1237	318	20.45
2004	SCREEN_COMMON_H_WK	1555	1409	146	9.39
2006	INCOME_TERTILE	1251	1230	21	1.68
2006	BMI	1251	1113	138	11.03
2006	FAT_PCT_ENERGY	1251	1206	45	3.6
2006	PA_TOTAL_MET_H_WK	1251	1035	216	17.27
2006	SCREEN_COMMON_H_WK	1251	1185	66	5.28
2009	INCOME_TERTILE	1136	1118	18	1.58

Survey year	Variable	Eligible N	Non-missing N	Missing N	Missing, %
2009	BMI	1136	1044	92	8.1
2009	FAT_PCT_ENERGY	1136	1111	25	2.2
2009	PA_TOTAL_MET_H_WK	1136	1037	99	8.71
2009	SCREEN_COMMON_H_WK	1136	1099	37	3.26
2011	INCOME_TERTILE	1550	1522	28	1.81
2011	BMI	1550	1489	61	3.94
2011	FAT_PCT_ENERGY	1550	1491	59	3.81
2011	PA_TOTAL_MET_H_WK	1550	1391	159	10.26
2011	SCREEN_COMMON_H_WK	1550	1507	43	2.77

Supplementary Table S3. Average posterior classification probabilities for the selected five-profile solution.

Profile ID	Profile label	Modal n	Modal %	Average maximum posterior probability	Mean P(P1)	Mean P(P2)	Mean P(P3)	Mean P(P4)	Mean P(P5)
1	Very low activity	282	6.9	0.815	0.815	0.104	0.0	0.081	0.0
2	Low-to-moderate activity / lower fat	1556	37.8	0.703	0.07	0.703	0.0	0.145	0.081
3	No reported entertainment screen time	221	5.4	1.0	0.0	0.0	1.0	0.0	0.0
4	Higher-fat	1142	27.8	0.641	0.038	0.145	0.0	0.641	0.176
5	Highly active	911	22.2	0.697	0.004	0.083	0.0	0.216	0.697

Supplementary Table S4. Fully adjusted concurrent associations between all selected behavioral profiles and overweight/obesity in stratified analyses.

Stratified analysis	Profile ID	Profile label	N	Unique participants	Events	OR (95% CI)	P value	Global profile P value
Sex: male	1	Very low activity	2098	1414	437	1.20 (0.73-1.99)	0.474	0.838
Sex: male	3	No reported entertainment screen time	2098	1414	437	1.24 (0.67-2.29)	0.501	0.838
Sex: male	4	Higher-fat	2098	1414	437	1.01 (0.68-1.49)	0.972	0.838
Sex: male	5	Highly active	2098	1414	437	0.95 (0.64-1.40)	0.792	0.838
Sex: female	1	Very low activity	1970	1343	279	1.14 (0.67-1.95)	0.617	0.809
Sex: female	3	No reported entertainment screen time	1970	1343	279	0.76 (0.41-1.44)	0.404	0.809
Sex: female	4	Higher-fat	1970	1343	279	0.89 (0.55-1.44)	0.633	0.809
Sex: female	5	Highly active	1970	1343	279	0.88 (0.53-1.47)	0.629	0.809

Stratified analysis	Profile ID	Profile label	N	Unique participants	Events	OR (95% CI)	P value	Global profile P value
Residence: rural	1	Very low activity	2799	1826	423	1.29 (0.82-2.05)	0.275	0.758
Residence: rural	3	No reported entertainment screen time	2799	1826	423	0.86 (0.47-1.55)	0.606	0.758
Residence: rural	4	Higher-fat	2799	1826	423	0.99 (0.68-1.45)	0.967	0.758
Residence: rural	5	Highly active	2799	1826	423	0.97 (0.65-1.44)	0.873	0.758
Residence: urban	1	Very low activity	1269	931	293	0.96 (0.51-1.79)	0.889	0.867
Residence: urban	3	No reported entertainment screen time	1269	931	293	1.20 (0.60-2.38)	0.609	0.867
Residence: urban	4	Higher-fat	1269	931	293	0.90 (0.55-1.46)	0.663	0.867
Residence: urban	5	Highly active	1269	931	293	0.83 (0.50-1.39)	0.486	0.867
Age: 6-11 years	1	Very low activity	2235	1708	485	1.14 (0.74-1.76)	0.545	0.853
Age: 6-11 years	3	No reported entertainment screen time	2235	1708	485	1.29 (0.71-2.36)	0.405	0.853
Age: 6-11 years	4	Higher-fat	2235	1708	485	1.01 (0.69-1.48)	0.973	0.853
Age: 6-11 years	5	Highly active	2235	1708	485	0.95 (0.63-1.43)	0.805	0.853
Age: 12-17 years	1	Very low activity	1833	1507	231	1.37 (0.65-2.88)	0.403	0.716
Age: 12-17 years	3	No reported entertainment screen time	1833	1507	231	0.78 (0.40-1.54)	0.475	0.716
Age: 12-17 years	4	Higher-fat	1833	1507	231	0.89 (0.54-1.46)	0.634	0.716
Age: 12-17 years	5	Highly active	1833	1507	231	0.87 (0.52-1.44)	0.580	0.716

Supplementary Table S5. Joint tests of interaction by sex, residence, and age group.

Effect modifier	N	Unique participants	Pseudo-class draws	Wald chi-square	df	Interaction P value
Sex	4068	2757	100	2.838	4	0.585
Residence	4068	2757	100	1.338	4	0.855
Age group	4068	2757	100	3.075	4	0.545

Supplementary Table S6. Sensitivity analyses of concurrent associations between selected behavioral profiles and overweight/obesity.

Sensitivity analysis	Profile ID	Profile label	N	Unique participants	Events	OR (95% CI)	P value	Global profile P value
Modal assignment sensitivity	1	Very low activity	4068	2757	716	1.27 (0.91-1.77)	0.154	0.579
Modal assignment sensitivity	3	No reported entertainment screen time	4068	2757	716	1.01 (0.66-1.53)	0.979	0.579
Modal assignment sensitivity	4	Higher-fat	4068	2757	716	1.00 (0.79-1.25)	0.978	0.579
Modal assignment sensitivity	5	Highly active	4068	2757	716	0.93 (0.72-1.20)	0.569	0.579
High-confidence posterior >=0.70	1	Very low activity	2103	1697	362	1.17 (0.75-1.84)	0.493	0.797
High-confidence posterior >=0.70	3	No reported entertainment screen time	2103	1697	362	0.93 (0.57-1.52)	0.782	0.797
High-confidence posterior >=0.70	4	Higher-fat	2103	1697	362	0.89 (0.57-1.39)	0.602	0.797
High-confidence posterior >=0.70	5	Highly active	2103	1697	362	0.88 (0.57-1.37)	0.574	0.797
Latest available wave per participant	1	Very low activity	2757	2757	479	1.37 (0.88-2.15)	0.166	0.404
Latest available wave per participant	3	No reported entertainment screen time	2757	2757	479	1.10 (0.66-1.85)	0.706	0.404
Latest available wave per participant	4	Higher-fat	2757	2757	479	0.93 (0.64-1.33)	0.683	0.404
Latest available wave per participant	5	Highly active	2757	2757	479	0.90 (0.62-1.31)	0.570	0.404
Age 7-17 years	1	Very low activity	3789	2597	637	1.18 (0.79-1.75)	0.424	0.812
Age 7-17 years	3	No reported entertainment screen time	3789	2597	637	0.87 (0.54-1.41)	0.581	0.812
Age 7-17 years	4	Higher-fat	3789	2597	637	0.96 (0.70-1.33)	0.822	0.812
Age 7-17 years	5	Highly active	3789	2597	637	0.94 (0.69-1.29)	0.704	0.812
Obesity-only outcome	1	Very low activity	4068	2757	300	1.19 (0.72-1.99)	0.499	0.919
Obesity-only outcome	3	No reported entertainment screen time	4068	2757	300	1.04 (0.56-1.95)	0.898	0.919
Obesity-only outcome	4	Higher-fat	4068	2757	300	0.94 (0.61-1.46)	0.780	0.919
Obesity-only outcome	5	Highly active	4068	2757	300	0.98 (0.63-1.54)	0.945	0.919
Fully adjusted except province fixed effects	1	Very low activity	4068	2757	716	1.35 (0.93-1.94)	0.111	0.272
Fully adjusted except province fixed effects	3	No reported entertainment screen time	4068	2757	716	1.10 (0.73-1.66)	0.658	0.272
Fully adjusted except province fixed effects	4	Higher-fat	4068	2757	716	0.96 (0.72-1.28)	0.782	0.272

Sensitivity analysis	Profile ID	Profile label	N	Unique participants	Events	OR (95% CI)	P value	Global profile P value
Fully adjusted except province fixed effects	5	Highly active	4068	2757	716	0.90 (0.67-1.21)	0.483	0.272

Supplementary Table S7. Pairwise comparisons between selected behavioral profiles in the fully adjusted model.

Profile A	Profile B	OR for A vs B (95% CI)	P value
Very low activity	Low-to-moderate activity / lower fat	1.18 (0.82-1.69)	0.387
Very low activity	No reported entertainment screen time	1.19 (0.72-1.95)	0.504
Very low activity	Higher-fat	1.22 (0.84-1.77)	0.288
Very low activity	Highly active	1.27 (0.86-1.86)	0.227
Low-to-moderate activity / lower fat	No reported entertainment screen time	1.01 (0.65-1.56)	0.969
Low-to-moderate activity / lower fat	Higher-fat	1.04 (0.77-1.41)	0.804
Low-to-moderate activity / lower fat	Highly active	1.08 (0.80-1.46)	0.621
No reported entertainment screen time	Higher-fat	1.03 (0.67-1.59)	0.893
No reported entertainment screen time	Highly active	1.07 (0.69-1.67)	0.767
Higher-fat	Highly active	1.04 (0.75-1.43)	0.819

Supplementary Table S8. Regression model specifications.

Analysis component	Specification
Model 1	behavioral profile only
Model 2	Model 1 + age, sex, and survey year
Model 3	Model 2 + residence, wave-specific household income tertile, and urbanicity index
Model 4	Model 3 + province fixed effects and total energy intake
Classification uncertainty	100 pseudo-class draws sampled from individual posterior probabilities; coefficients and covariance matrices pooled using Rubin-type rules
Repeated observations	Cluster-robust standard errors at the participant (IDIND) level

Supplementary Table S9. Additional robustness checks for profile estimation and covariance specification.

Robustness check	Sample/method	Key result	Interpretation
Behavior-complete profile estimation	n=4,441; complete physical activity, screen time, and fat-energy-share indicators; BMI not required	Five-profile BIC=34,450.19; six-profile BIC=34,460.22; entropy=0.557; minimum modal profile=5.3%; ARI versus primary solution=0.952 among n=4,112	The selected profile structure was not materially driven by BMI availability.
Community-clustered covariance	Model 4; n=4,068; 291 communities; 100 pseudo-class draws	P1 vs P2 OR=1.18 (95% CI, 0.80-1.73); global P=0.835	The null association conclusion was unchanged.
Two-way participant-and-community clustered covariance	Model 4; n=4,068; 2,757 participants; 291 communities; 100 pseudo-class draws	P1 vs P2 OR=1.18 (95% CI, 0.80-1.73); global P=0.834	The null association conclusion was unchanged.

Note. *ARI, adjusted Rand index; BIC, Bayesian information criterion; CI, confidence interval; OR, odds ratio. The profile labels from the behavior-complete analysis were aligned semantically to the primary five-profile solution before calculating agreement.*

Supplementary Table S10. Comparison of school-attending records included in and excluded from the primary LPA.

Characteristic	Included in primary LPA (n=4,112)	Excluded school-attending records (n=1,380)	SMD
Age, years	11.10	11.43	-0.104
Male, %	51.65	55.51	-0.077
Urban residence, %	31.35	28.70	0.058
2011 survey wave, %	31.23	19.28	0.278
CPI-adjusted household income per capita	9,405.05	8,176.16	0.113
Urbanicity index	64.98	63.61	0.068

Note. *SMD, standardized mean difference. Positive SMDs indicate higher values or proportions in the included group. SMDs are descriptive and were not used as formal hypothesis tests.*

Supplementary Table S11. Sensitivity of profile enumeration to alternative handling of high physical-activity values.

Physical-activity specification	Best profile solution	BIC (5 profiles)	BIC (6 profiles)	Entropy (5 profiles)	Minimum profile (%)	Agreement with primary (%)	ARI vs. primary
Primary domain-time caps	5 profiles	31,879.83	31,887.93	0.562	5.4	100.0	1.000
Uncapped physical activity	5 profiles	31,902.85	31,916.22	0.549	5.4	98.6	0.963
Uncapped, P99 winsorized	5 profiles	31,863.44	31,867.96	0.570	5.4	98.7	0.966

Note. *ARI, adjusted Rand index; BIC, Bayesian information criterion. In the primary specification, school, leisure, and active-travel time were capped at 40, 60, and 20 h/week, respectively. The caps affected 21 of 4,112 observations (0.51%). The 99th percentile of the uncapped total physical-activity distribution was 209.89 MET-h/week. Agreement and ARI compare modal assignments with the primary capped five-profile solution.*

Supplementary Table S12. Equal-variance Mplus auxiliary profile-enumeration results.

Profiles	LL	AIC	BIC	SABIC	Entropy	Min estimated %	Min modal n	Adjusted LMR P	BLRT P
1	-17,504.026	35,020.051	35,057.981	35,038.916	—	100.00	4,112	—	—
2	-17,068.329	34,156.658	34,219.875	34,188.099	0.945	7.455	275	<0.0001	<0.0001
3	-16,998.818	34,025.635	34,114.138	34,069.652	0.911	4.067	127	<0.0001	<0.0001
4	-16,958.882	33,953.764	34,067.554	34,010.358	0.681	3.904	130	<0.0001	<0.0001
5	-16,927.338	33,898.677	34,037.753	33,967.847	0.742	0.684	24	<0.0001	<0.0001
6	-16,896.548	33,845.097	34,009.460	33,926.843	0.670	0.679	24	0.0001	<0.0001

Note. *Indicator variances were constrained equal across profiles. Likelihood-ratio tests favored additional profiles, but the five- and six-profile models each contained a <1% profile; results were therefore treated as auxiliary sensitivity evidence.*

Supplementary Table S13. Wave- and age-stratified profile enumeration and fixed five-profile alignment.

Grouping	Stratum	n	BIC-preferred k	5-profile entropy	5-profile avg max posterior	Mean center cosine similarity	Bootstrap mean ARI
Survey wave	2004	1,037	2	0.528	0.675	0.843	0.448
Survey wave	2006	873	5	0.632	0.742	0.986	0.406
Survey wave	2009	918	5	0.481	0.648	0.987	0.429
Survey wave	2011	1,284	6	0.571	0.712	0.971	0.531
Age group	6-11	2,257	4	0.569	0.719	0.982	0.646
Age group	12-17	1,855	6	0.594	0.728	0.965	0.700

Note. *Group-specific indicators used pooled-sample transformation parameters. Center similarity and ARI refer to fixed five-profile models aligned to the pooled reference solution.*

Supplementary Table S14. Posterior-weighted common-profile composition by survey wave and age group.

Grouping	Stratum	P1 very low activity, %	P2 low-to-moderate/lower fat, %	P3 no reported entertainment screen time, %	P4 higher-fat, %	P5 highly active, %
Survey wave	2004	6.52	36.51	7.81	24.85	24.32
Survey wave	2006	9.91	35.13	5.61	26.00	23.35
Survey wave	2009	11.39	33.89	3.16	27.95	23.61
Survey wave	2011	9.92	28.70	4.83	33.98	22.58
Age group	6-11	12.28	36.87	3.23	28.20	19.42
Age group	12-17	5.87	28.72	7.98	29.17	28.27

Supplementary Table S15. Sensitivity of profile enumeration and membership to covariance, transformation, and screen-zero specifications.

Specification	Transformation	Covariance/model	BIC-preferred k	BIC for 5 profiles	5-profile entropy	Minimum class, %	ARI vs primary
Primary class-specific diagonal	log1p_primary	diag	5	31,880.14	0.559	5.37	0.991
Class-specific full covariance	log1p_primary	full	3	31,955.98	0.575	5.37	0.771
Shared full covariance	log1p_primary	tied	6	34,184.39	0.490	3.79	0.711
Class-specific spherical variance	log1p_primary	spherical	6	34,238.69	0.526	5.06	0.686
Raw indicators, diagonal covariance	raw_z	diag	6	29,541.43	0.605	4.33	0.256
Yeo-Johnson transformed, diagonal covariance	yeo_johnson	diag	5	32,927.92	0.400	5.37	0.450
Rank-normal transformed, diagonal covariance	rank_normal	diag	6	32,615.82	0.617	2.48	0.253
Zero-inflated hurdle screen model	log1p / point mass at zero	class-specific diagonal	5	33,715.51	0.759	0.75	0.087

Note. *BIC comparisons are valid within, not across, different distribution and transformation families. The hurdle model explicitly separated zero from positive entertainment screen time.*

Supplementary Table S16. Profile-specific classification quality and entertainment-screen-time zero values.

Profile	Label	Modal n (%)	Mean assigned posterior	Median assigned posterior	Assigned posterior <0.70, %	Screen time = 0, %
P1	Very low activity	282 (6.9)	0.815	0.870	31.6	0.0
P2	Low-to-moderate activity / lower fat	1,556 (37.8)	0.703	0.728	44.9	0.0
P3	No reported entertainment screen time	221 (5.4)	1.000	1.000	0.0	100.0
P4	Higher-fat	1,142 (27.8)	0.641	0.620	64.7	0.0
P5	Highly active	911 (22.2)	0.697	0.698	50.3	0.0

Supplementary Table S17. Missingness of primary indicators, BMI, and household-income tertile by survey wave.

Wave	Variable	Eligible n	Nonmissing n	Missing n	Missing, %
Overall	Physical activity	5,492	4,700	792	14.42
Overall	Entertainment screen time	5,492	5,200	292	5.32
Overall	Fat-energy share	5,492	5,282	210	3.82
Overall	BMI	5,492	5,046	446	8.12
Overall	Household income tertile	5,492	5,405	87	1.58
2004	Physical activity	1,555	1,237	318	20.45
2004	Entertainment screen time	1,555	1,409	146	9.39
2004	Fat-energy share	1,555	1,474	81	5.21
2004	BMI	1,555	1,400	155	9.97
2004	Household income tertile	1,555	1,535	20	1.29
2006	Physical activity	1,251	1,035	216	17.27
2006	Entertainment screen time	1,251	1,185	66	5.28
2006	Fat-energy share	1,251	1,206	45	3.60
2006	BMI	1,251	1,113	138	11.03
2006	Household income tertile	1,251	1,230	21	1.68
2009	Physical activity	1,136	1,037	99	8.71
2009	Entertainment screen time	1,136	1,099	37	3.26
2009	Fat-energy share	1,136	1,111	25	2.20
2009	BMI	1,136	1,044	92	8.10
2009	Household income tertile	1,136	1,118	18	1.58
2011	Physical activity	1,550	1,391	159	10.26
2011	Entertainment screen time	1,550	1,507	43	2.77
2011	Fat-energy share	1,550	1,491	59	3.81
2011	BMI	1,550	1,489	61	3.94
2011	Household income tertile	1,550	1,522	28	1.81

Supplementary Table S18. Selection-model and stabilized inverse-probability-weight diagnostics.

Diagnostic	Value	Interpretation
Inclusion model AUC	0.676	Discrimination of primary-LPA inclusion from fully observed predictors
Stabilized weight minimum	0.800	No extreme low weight
Stabilized weight maximum	1.762	No extreme high weight
Effective sample size	4,003	Compared with n=4,112
Maximum |SMD| before IPW	0.070	Across modeled characteristics
Maximum |SMD| after IPW	0.005	Substantial observed-balance improvement
Largest profile-composition change	0.28 percentage points	Posterior-weighted composition after IPW

Supplementary Table S19. Sensitivity of the fully adjusted binary association to missing income and selection weighting.

Analysis	n	P1 vs P2 OR (95% CI)	Global profile P	Comment
Primary fully adjusted analysis	4,068	1.18 (0.81-1.69)	0.806	Original prespecified complete-case result
20-imputation income-tertile sensitivity	4,112	1.18 (0.82-1.71)	0.752	Missing income tertile imputed
IPW + 20-imputation sensitivity	4,112	1.17 (0.80-1.71)	0.822	Selection weights combined with imputation

Supplementary Table S20. Fully adjusted multinomial associations with overweight and obesity.

Outcome vs normal/underweight	Profile	Profile label	RRR (95% CI)	P	Outcome-specific global P
Overweight	P1	Very low activity	1.11 (0.69-1.79)	0.664	0.935
Overweight	P3	No reported entertainment screen time	0.94 (0.55-1.61)	0.824	0.935
Overweight	P4	Higher-fat	0.99 (0.68-1.44)	0.960	0.935
Overweight	P5	Highly active	0.90 (0.62-1.30)	0.565	0.935
Obesity	P1	Very low activity	1.25 (0.75-2.07)	0.394	0.846
Obesity	P3	No reported entertainment screen time	1.04 (0.55-1.97)	0.910	0.846
Obesity	P4	Higher-fat	0.93 (0.59-1.47)	0.765	0.846
Obesity	P5	Highly active	0.99 (0.63-1.55)	0.952	0.846

Note. *The reference profile was P2. The joint eight-degree-of-freedom multinomial profile test was P = 0.980.*

Supplementary Table S21. Fully adjusted continuous BMI and approximate WHO BMI-for-age z-score associations.

Outcome specification	Profile	Profile label	Adjusted mean difference (95% CI)	P	Global P
WHO BAZ, completed-age lower bound	P1	Very low activity	0.077 (-0.120 to 0.275)	0.441	0.854
WHO BAZ, completed-age lower bound	P3	No reported entertainment screen time	0.044 (-0.131 to 0.219)	0.623	0.854
WHO BAZ, completed-age lower bound	P4	Higher-fat	0.021 (-0.112 to 0.153)	0.761	0.854
WHO BAZ, completed-age lower bound	P5	Highly active	-0.023 (-0.156 to 0.110)	0.734	0.854
WHO BAZ, completed-age midpoint	P1	Very low activity	0.081 (-0.112 to 0.275)	0.410	0.797
WHO BAZ, completed-age midpoint	P3	No reported entertainment screen time	0.055 (-0.120 to 0.230)	0.537	0.797
WHO BAZ, completed-age midpoint	P4	Higher-fat	0.019 (-0.112 to 0.151)	0.772	0.797
WHO BAZ, completed-age midpoint	P5	Highly active	-0.026 (-0.158 to 0.106)	0.702	0.797
WHO BAZ, completed-age upper bound	P1	Very low activity	0.085 (-0.106 to 0.277)	0.382	0.733
WHO BAZ, completed-age upper bound	P3	No reported entertainment screen time	0.065 (-0.110 to 0.240)	0.467	0.733
WHO BAZ, completed-age upper bound	P4	Higher-fat	0.018 (-0.113 to 0.150)	0.784	0.733
WHO BAZ, completed-age upper bound	P5	Highly active	-0.028 (-0.160 to 0.103)	0.671	0.733
WHO BAZ midpoint, plausible range -5 to +5	P1	Very low activity	0.063 (-0.117 to 0.242)	0.494	0.864
WHO BAZ midpoint, plausible range -5 to +5	P3	No reported entertainment screen time	0.060 (-0.114 to 0.234)	0.501	0.864
WHO BAZ midpoint, plausible range -5 to +5	P4	Higher-fat	0.014 (-0.113 to 0.142)	0.824	0.864
WHO BAZ midpoint, plausible range -5 to +5	P5	Highly active	-0.018 (-0.146 to 0.111)	0.789	0.864
Raw BMI, kg/m2	P1	Very low activity	0.209 (-0.238 to 0.657)	0.358	0.600
Raw BMI, kg/m2	P3	No reported entertainment screen time	0.196 (-0.235 to 0.626)	0.374	0.600
Raw BMI, kg/m2	P4	Higher-fat	-0.012 (-0.309 to 0.285)	0.937	0.600
Raw BMI, kg/m2	P5	Highly active	-0.083 (-0.380 to 0.214)	0.583	0.600

Note. *WHO BMI-for-age z scores were approximate because exact age in months was unavailable. The midpoint of the completed-age year was the principal sensitivity specification; lower- and upper-bound month assignments evaluated age-month uncertainty.*

Supplementary figure legends

Supplementary Figure S1. BIC across candidate profile solutions.

Lower BIC values indicate better relative fit after penalizing model complexity. The five-profile solution had the lowest BIC.

Supplementary Figure S2. Estimated prevalence of overweight/obesity by selected behavioral profile, accounting for profile-membership uncertainty.

Error bars are participant-cluster bootstrap 95% confidence intervals.

Supplementary Figure S3. Sequential attenuation of the association between the very-low-activity profile and overweight/obesity.

The reference group was the low-to-moderate activity/lower-fat profile. Covariates were added sequentially from Model 1 to Model 4.

Supplementary Figure S4. Bootstrap classification stability.

Bars represent the mean adjusted Rand index across 100 bootstrap replications; error bars represent one standard deviation.

Supplementary Figure S5. Posterior-weighted common-profile composition by survey wave.

See the corresponding supplementary table and main text for model definitions, reference categories, and interpretation.

Supplementary Figure S6. Posterior-weighted common-profile composition by age group.

See the corresponding supplementary table and main text for model definitions, reference categories, and interpretation.

Supplementary Figure S7. BIC-preferred profile count under alternative covariance, transformation, and screen-zero specifications.

See the corresponding supplementary table and main text for model definitions, reference categories, and interpretation.

Supplementary Figure S8. Profile-specific classification quality in the primary five-profile working solution.

See the corresponding supplementary table and main text for model definitions, reference categories, and interpretation.

Supplementary Figure S9. Missingness of key variables by survey wave.

See the corresponding supplementary table and main text for model definitions, reference categories, and interpretation.

Supplementary Figure S10. Covariate balance before and after stabilized inverse-probability weighting.

See the corresponding supplementary table and main text for model definitions, reference categories, and interpretation.

Supplementary Figure S11. Posterior-weighted normal/underweight, overweight, and obesity composition by profile.

See the corresponding supplementary table and main text for model definitions, reference categories, and interpretation.

Supplementary Figure S12. Fully adjusted multinomial relative risk ratios for overweight and obesity.

See the corresponding supplementary table and main text for model definitions, reference categories, and interpretation.

Supplementary Figure S13. Fully adjusted mean differences for continuous BMI and approximate WHO BMI-for-age z-score outcomes.

See the corresponding supplementary table and main text for model definitions, reference categories, and interpretation.

Supplementary figures

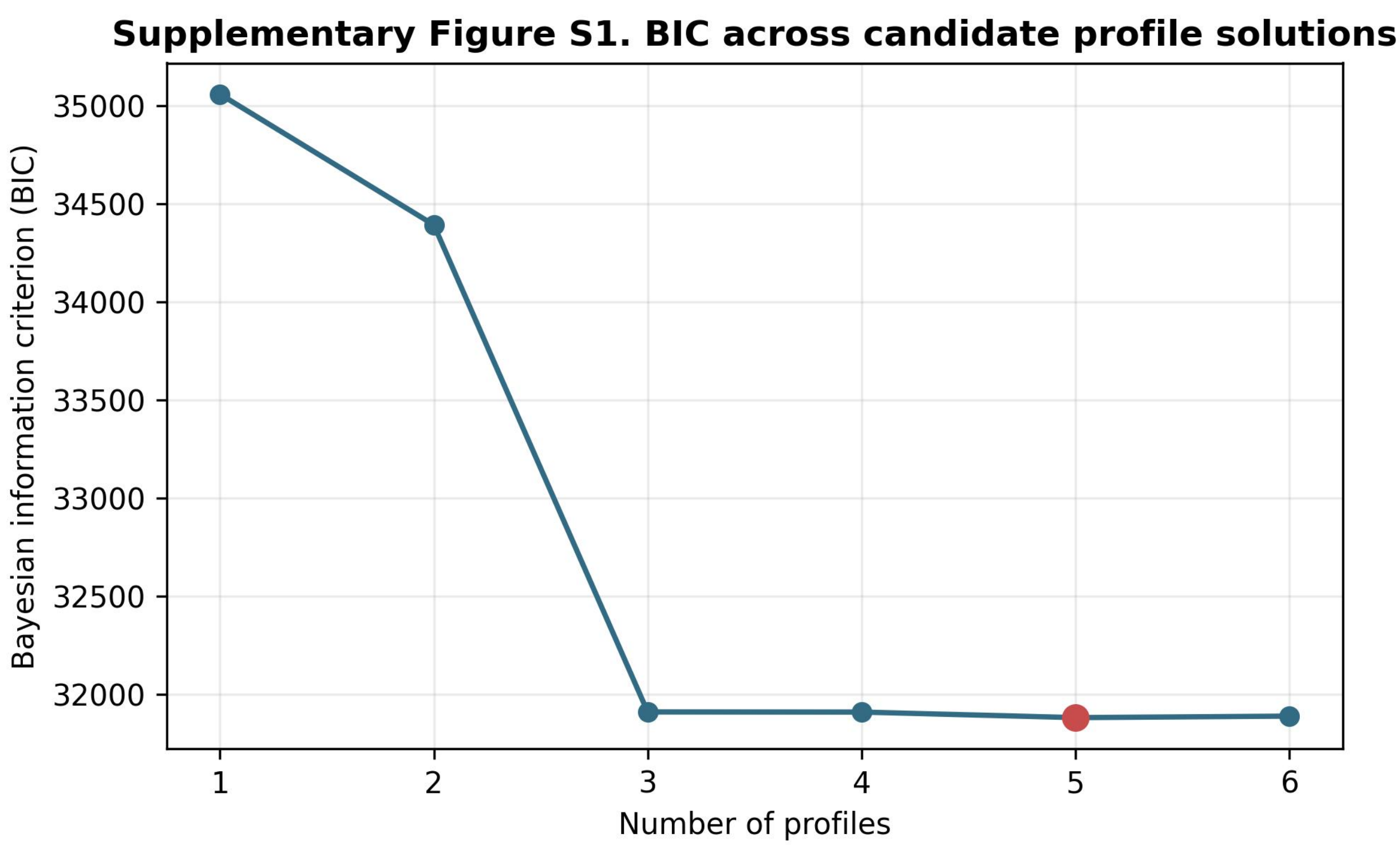

Supplementary Figure S2. Estimated overweight/obesity prevalence by selected behavioral profile

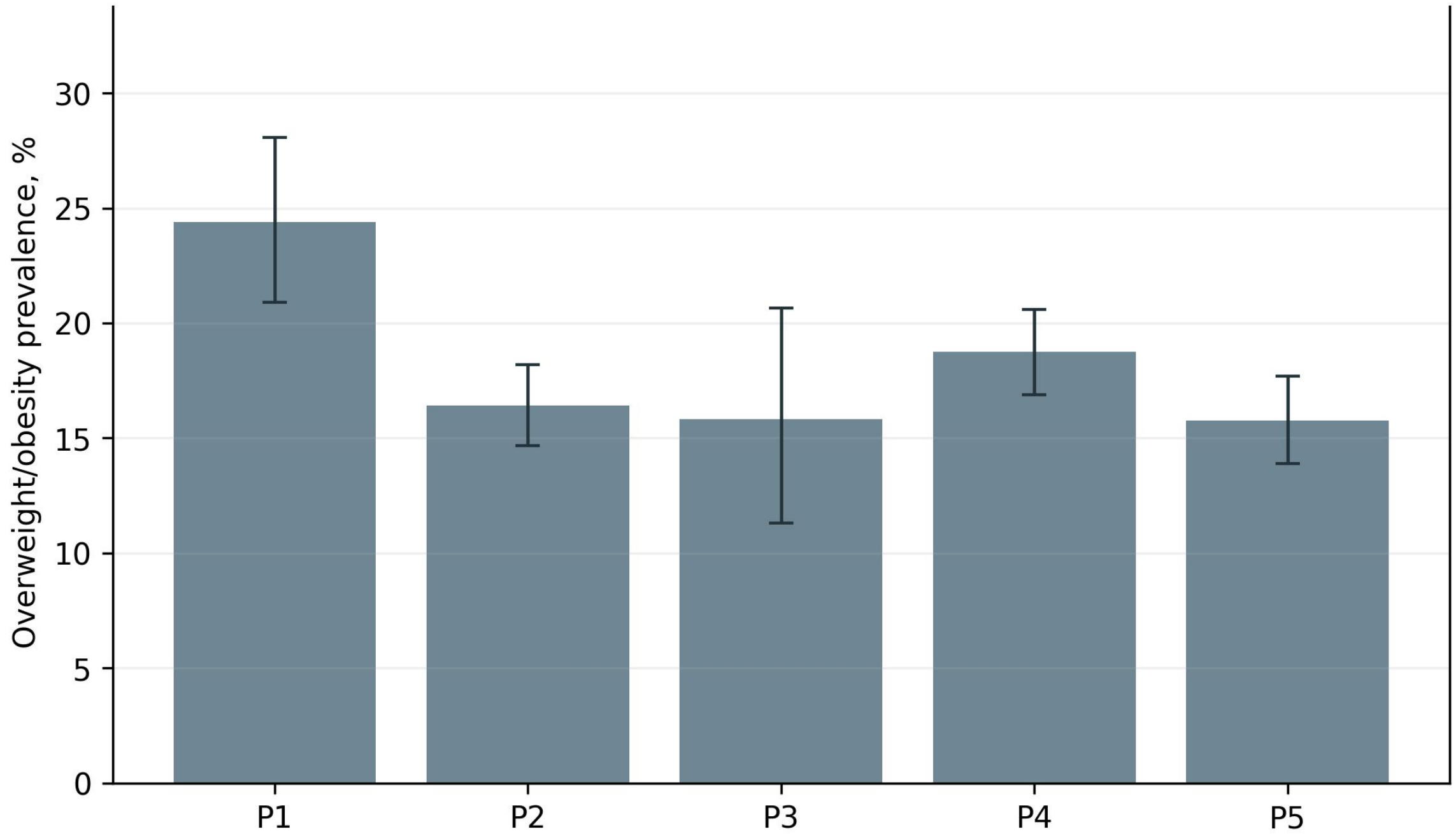

Supplementary Figure S3. Sequential attenuation for the very-low-activity profile

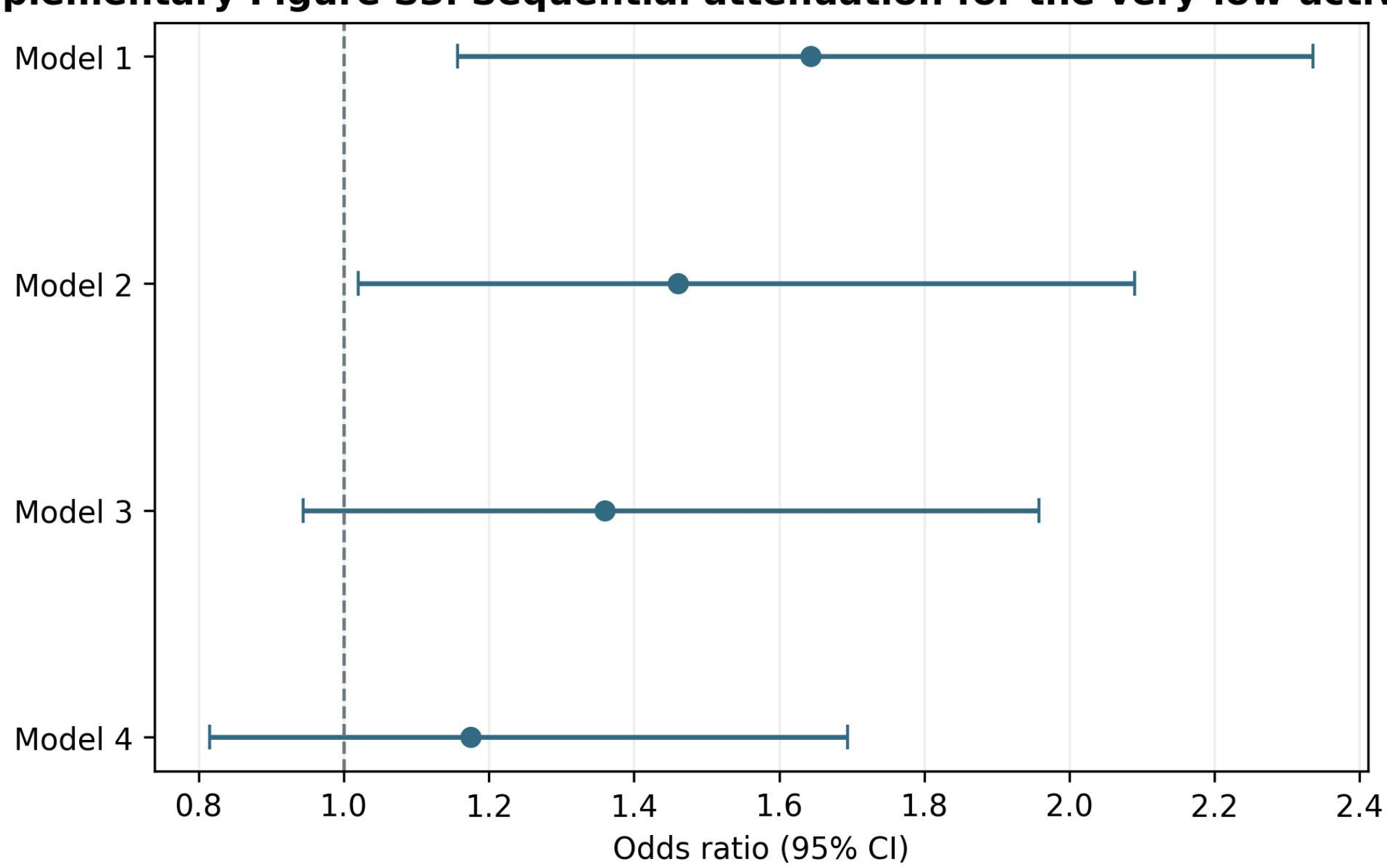

Supplementary Figure S4. Bootstrap classification stability

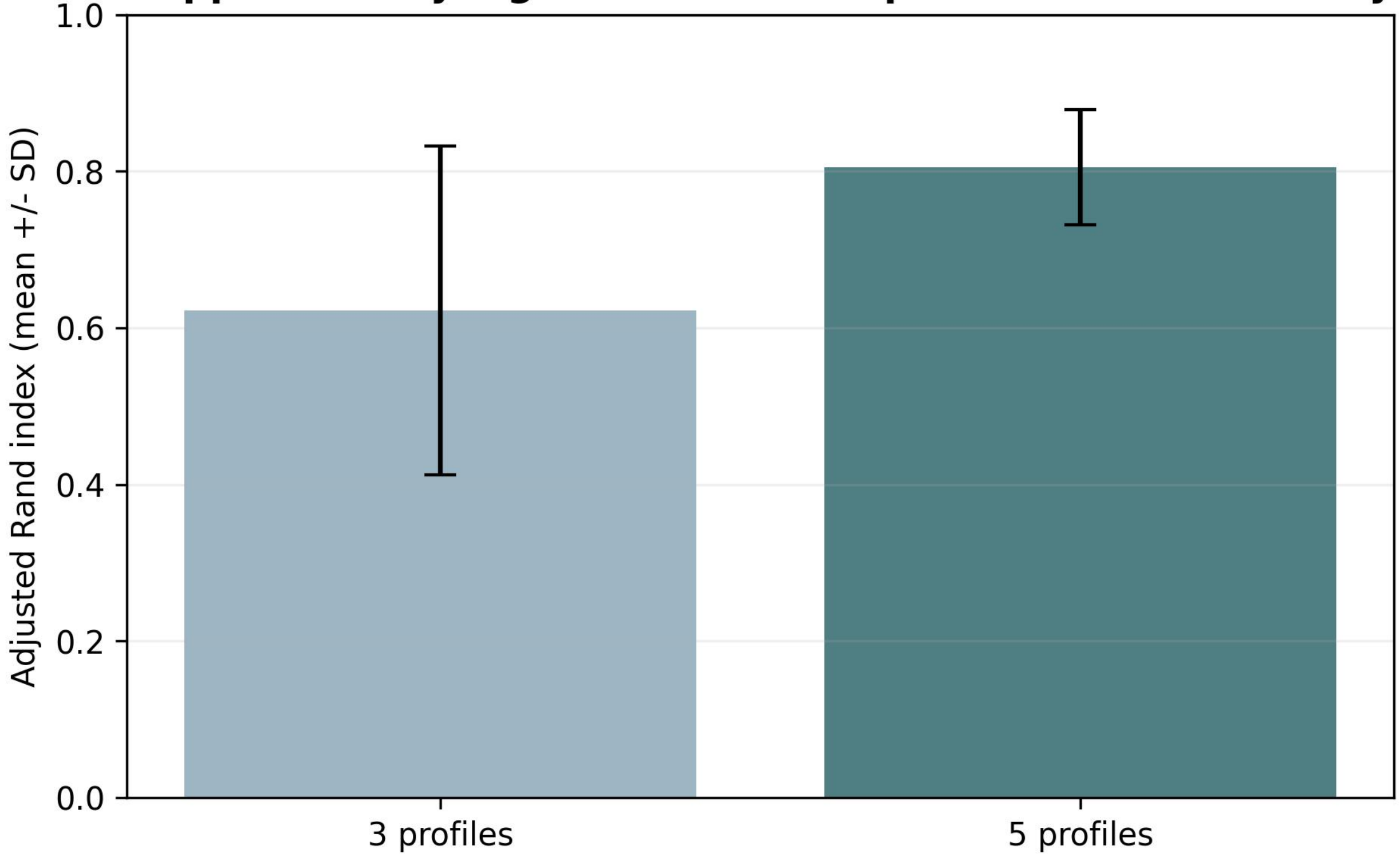

Supplementary Figure S5. Posterior-weighted common-profile composition by survey wave

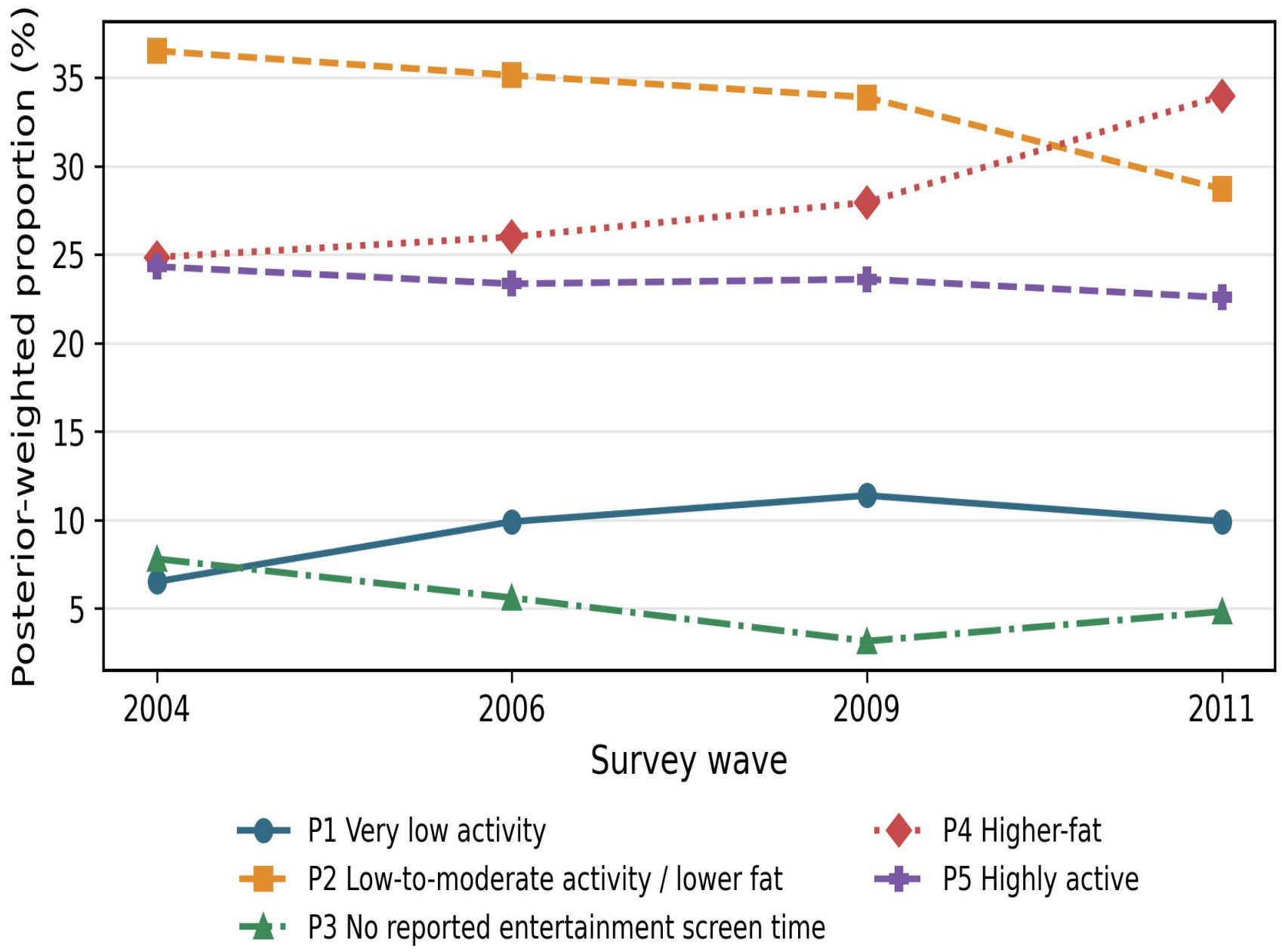

Supplementary Figure S6. Posterior-weighted common-profile composition by age group

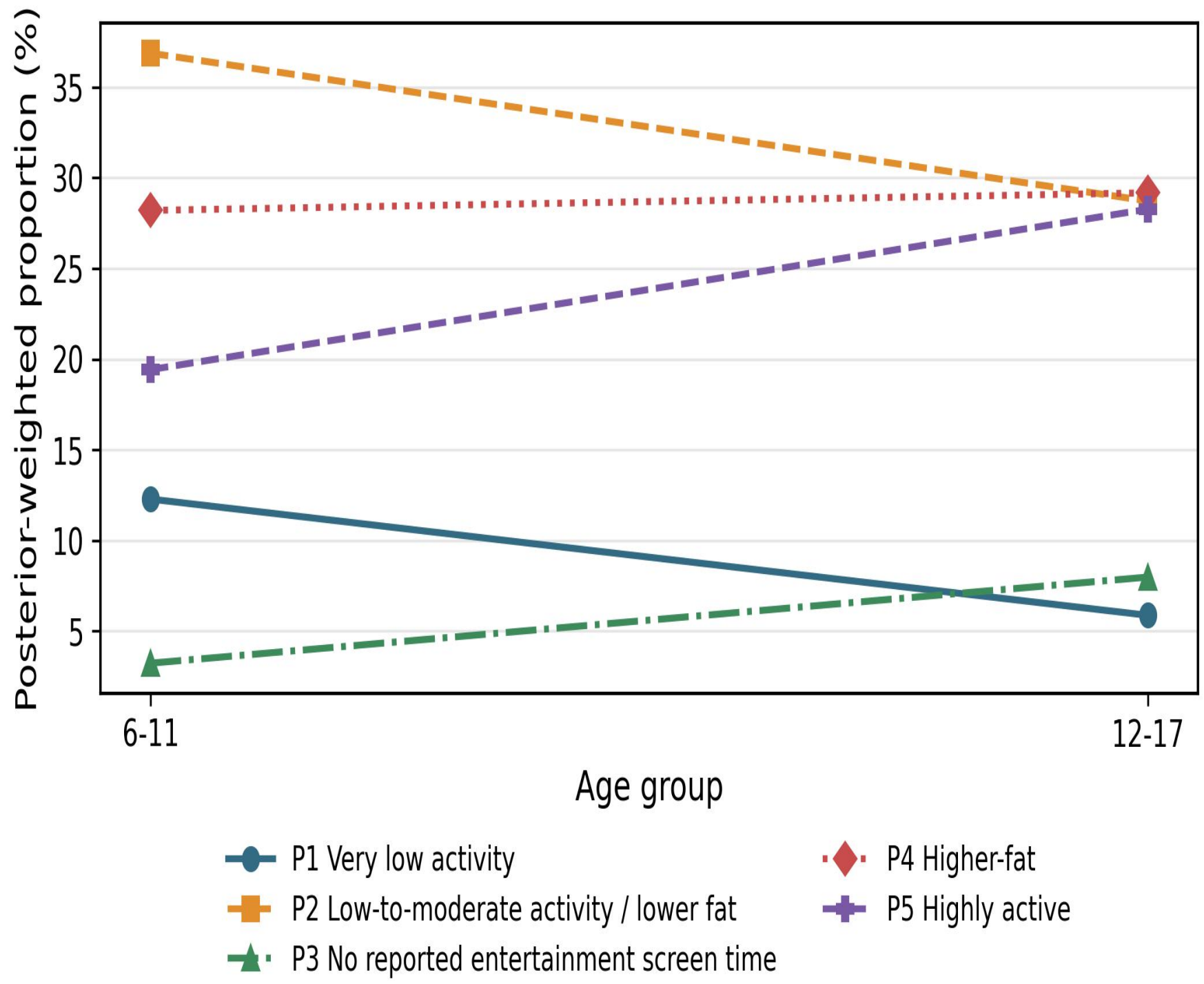

Supplementary Figure S7. BIC-preferred profile count under alternative model specifications

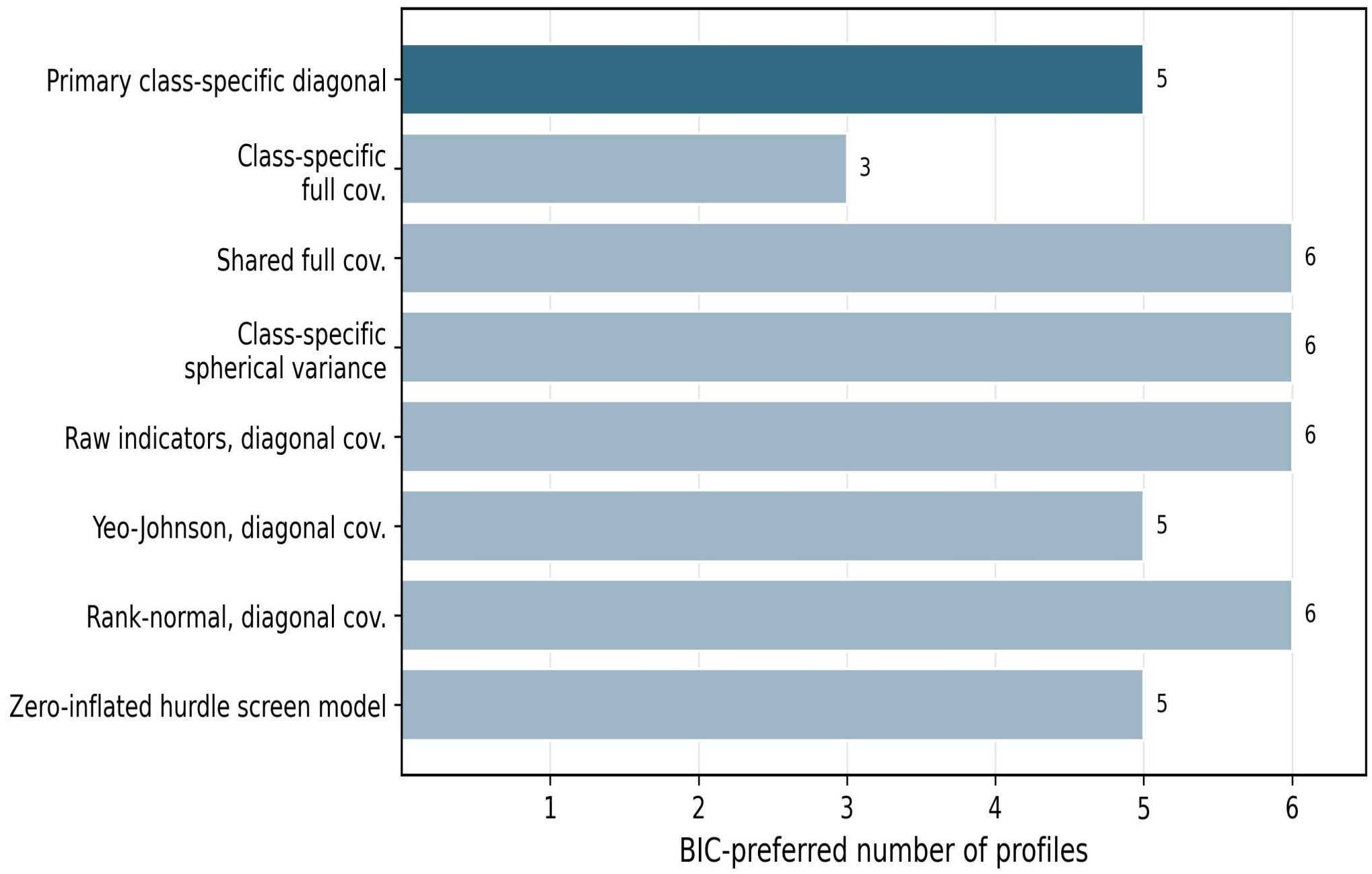

Supplementary Figure S8. Profile-specific classification quality in the primary five-profile solution

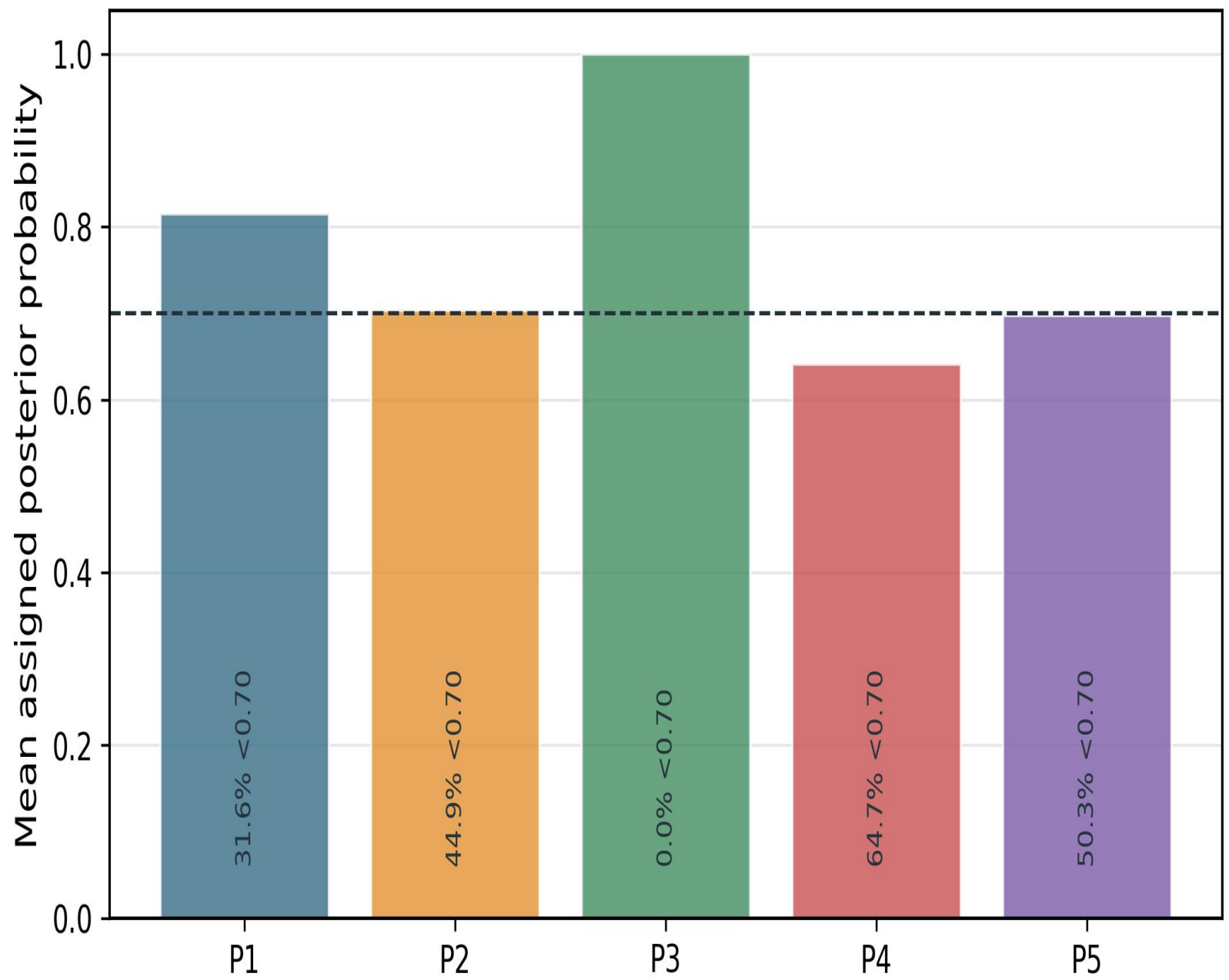

Supplementary Figure S9. Missingness of key variables by survey wave

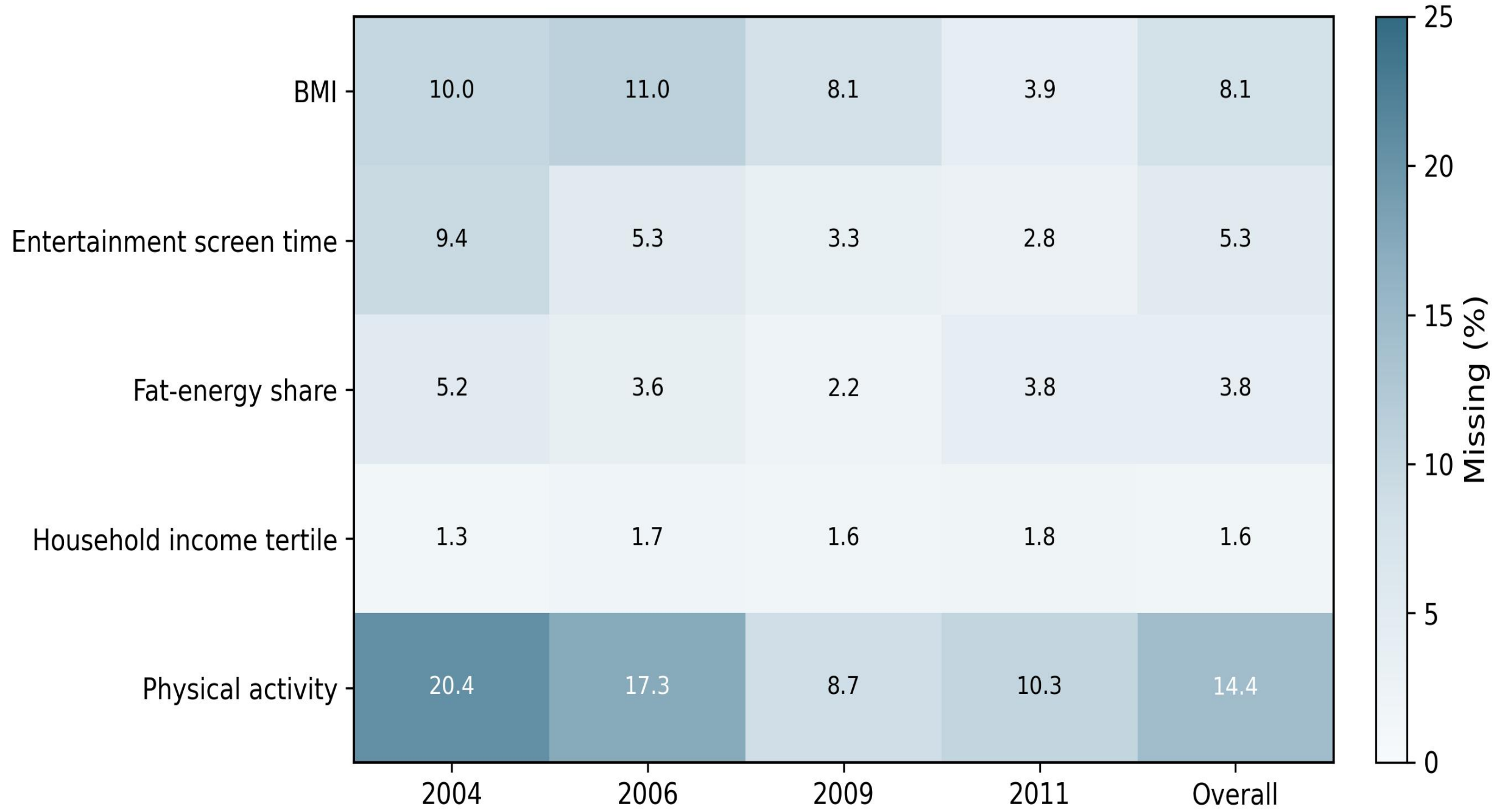

Supplementary Figure S10. Covariate balance before and after stabilized inverse-probability weighting

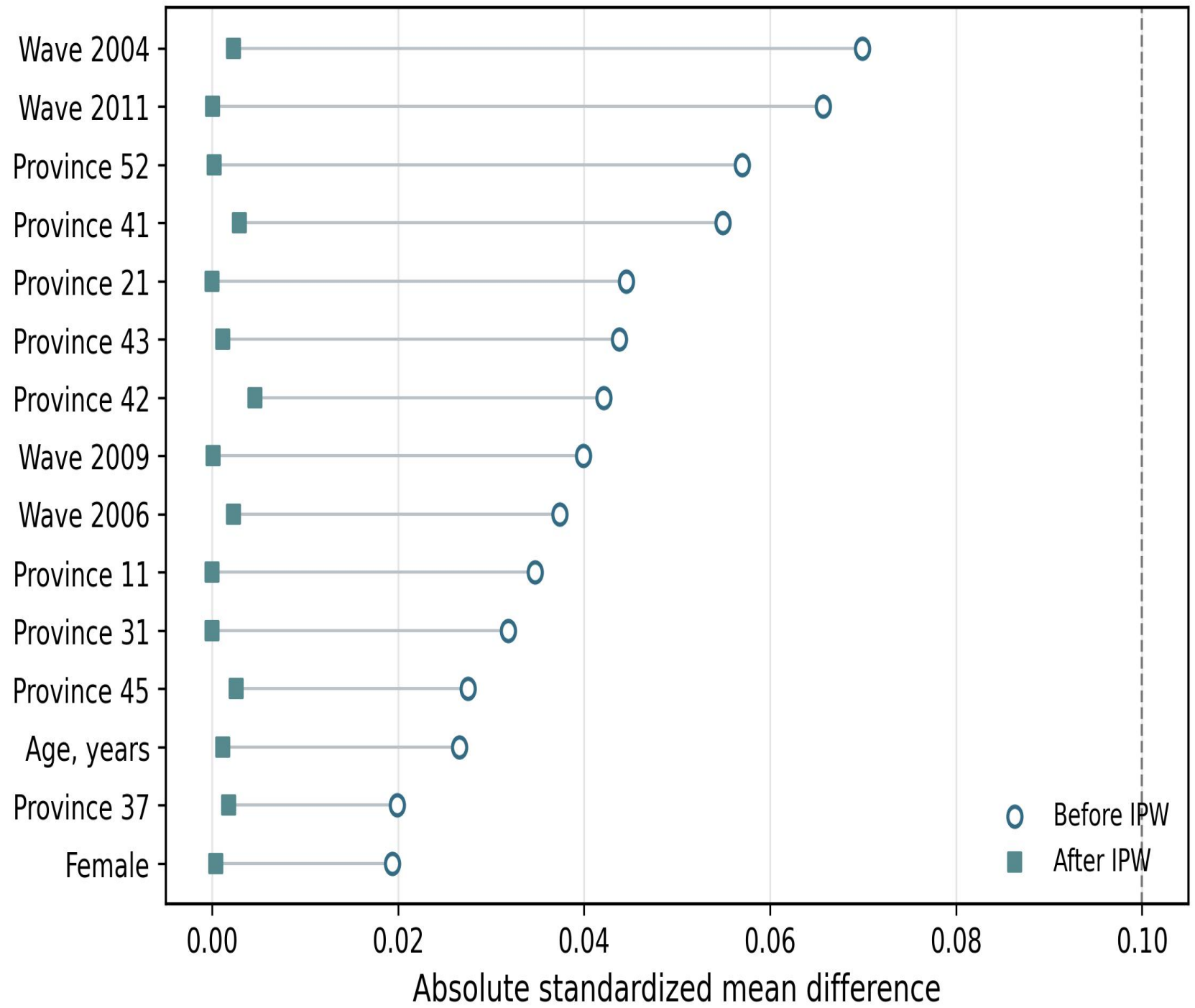

Supplementary Figure S11. Posterior-weighted weight-status composition by profile

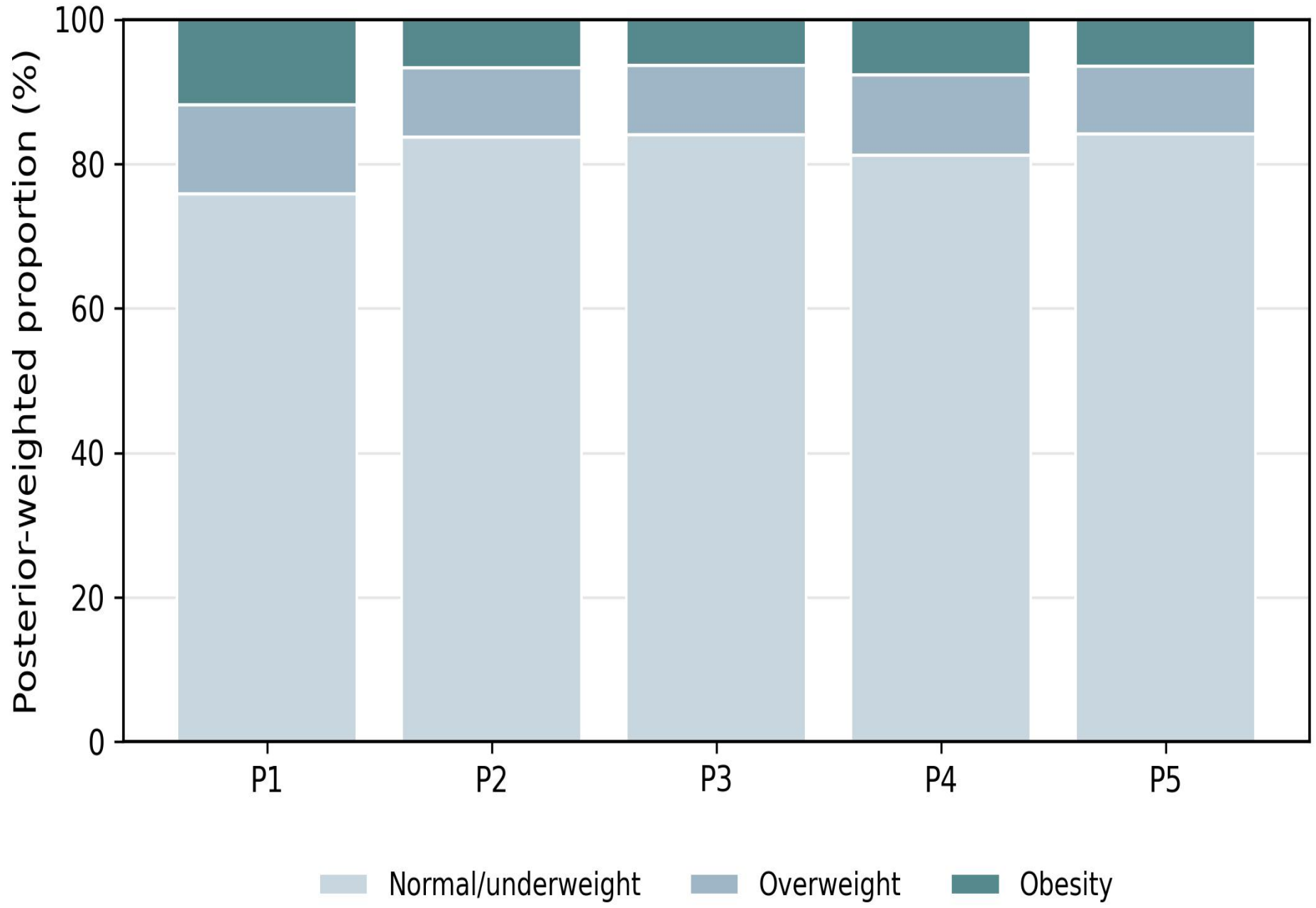

Supplementary Figure S12. Fully adjusted multinomial relative risk ratios for overweight and obesity

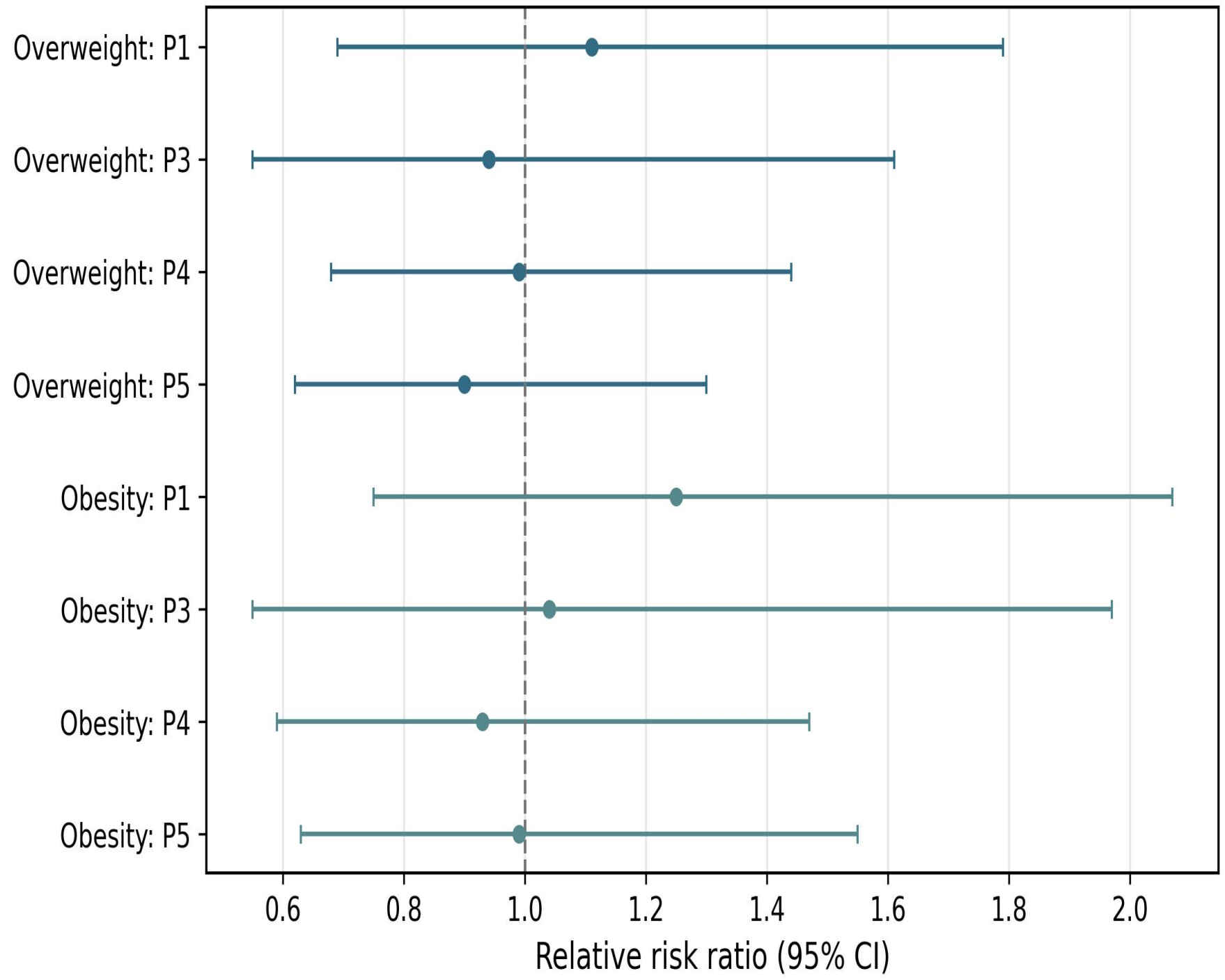

Supplementary Figure S13. Fully adjusted mean differences for continuous BMI and approximate WHO BMI-for-age z-score outcomes

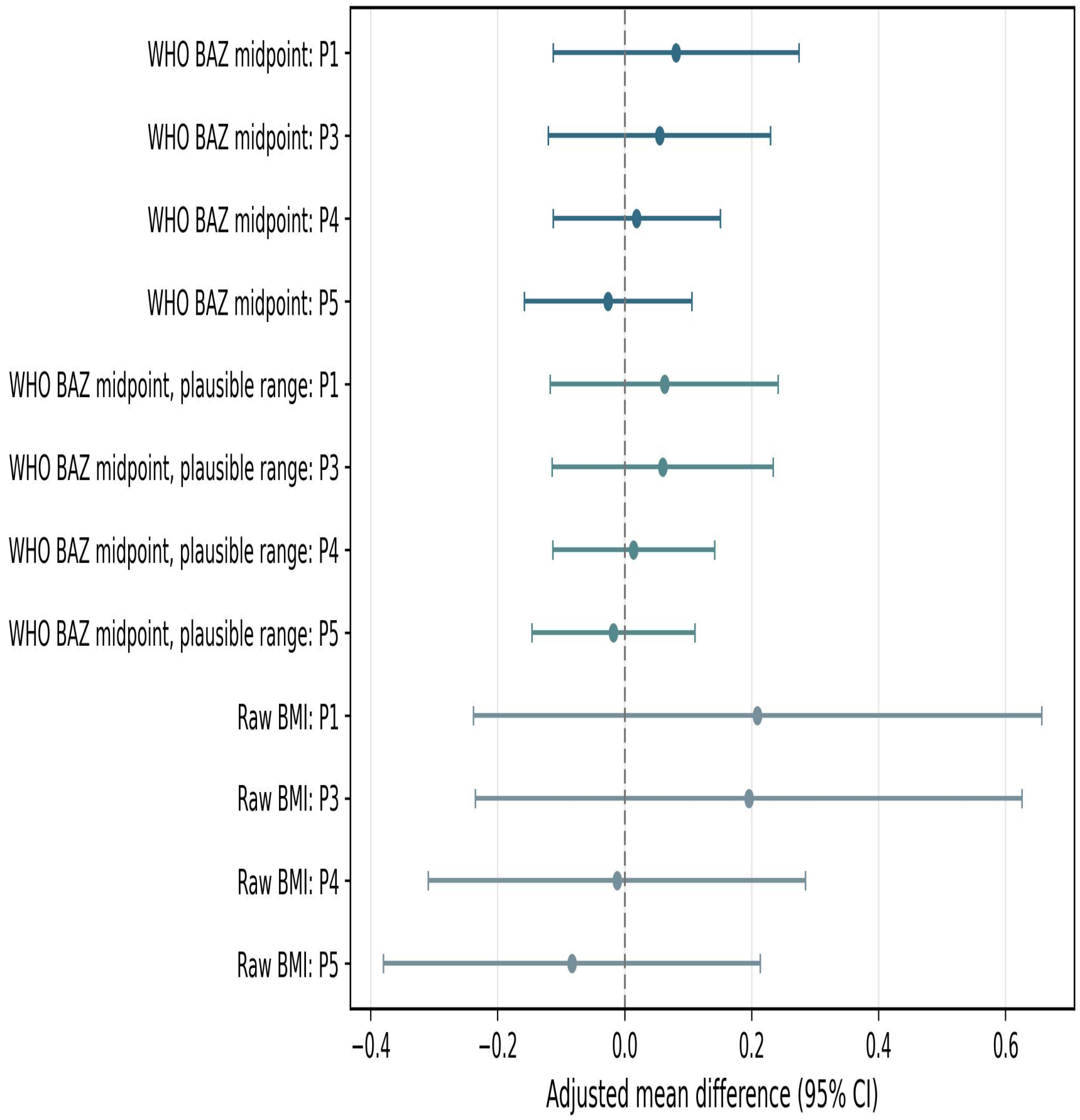

Supplement: Supplementary file 1 [file Data_Sheet_4.PDF]
