# Supplementary material for: Physical activity, entertainment screen time, and dietary fat profiles and concurrent weight status among school-attending Chinese children and adolescents: CHNS 2004–2011
Source: Front Public Health. 2026 Sep 28;14:1945546. doi: 10.3389/fpubh.2026.1945546 (PMC13654989; doi:10.3389/fpubh.2026.1945546)
Supplement: Supplementary file 2 [file Data_Sheet_1.ZIP › c12diet.pdf]

DATA SET: c12diet LABEL: CHNS Constructed Dietary Value(c12diet) 1989-2011 DATE CREATED: 09OCT14:16:13:17
Number of Observations: 102575
Number of Variables: 14
Organization of file: 1 Observation Individual/Year (IDind/WAVE)

Variable Name	Variable Label (VAR)	VAR Type	VAR Format	VAR Length	Mean	Range of Values	Frequency Category	Frequency	Percent
IDIND	INDIVIDUAL ID	Num		8	3.85895E11	1.11101E11 to 5.52304E11		102575	100.00
HHID	CURRENT HOUSEHOLD ID	Num		8	385894626	111101001 to 552304020		102575	100.00
LINE	CURRENT INDIVIDUAL LINE NUMBER	Num		8	8.41436022	1-146		102575	100.00
COMMID	COMMUNITY ID: T1-T4	Num		8	385894.603	111101-552304		102575	100.00
WAVE	Survey Year	Num	YR	8	2001.31856	1991	1991	13579	13.24
						1993	1993	12733	12.41
						1997	1997	12846	12.52
						2000	2000	13586	13.24
						2004	2004	11846	11.55
						2006	2006	11374	11.09
						2009	2009	11609	11.32
						2011	2011	15002	14.63
D3CARBO	3-Day Ave: Carbohydrate (g)	Num		8	318.792219	1.94-1993.80		102575	100.00
D3FAT	3-Day Ave: Fat (g)	Num		8	66.0048128	0.10-12615.66		102575	100.00
D3KCAL	3-Day Ave: Energy (kcal)	Num		8	2146.31344	9.51-115187.96		102575	100.00
D3PROTN	3-Day Ave: Protein (g)	Num		8	65.2254729	0.22-1396.15		102575	100.00
T1	PROVINCE	Num	PROV	8	38.4006239	11	Beijing, Added 2011	1299	1.27
						21	Liaoning, Missed 1997	8878	8.66
						23	Heilongjiang, Added 1997	7467	7.28

DATA SET: c12diet LABEL: CHNS Constructed Dietary Value(c12diet) 1989-2011 DATE CREATED: 09OCT14:16:13:17
Number of Observations: 102575
Number of Variables: 14
Organization of file: 1 Observation Individual/Year (IDind/WAVE)

Variable Name	Variable Label (VAR)	VAR Type	VAR Format	VAR Length	Mean	Range of Values	Frequency Category	Frequency	Percent
						31	Shanghai, Added 2011	1471	1.43
						32	Jiangsu	11102	10.82
						37	Shandong	10658	10.39
						41	Henan	12093	11.79
						42	Hubei	11273	10.99
						43	Hunan	11224	10.94
						45	Guangxi	13464	13.13
						52	Guizhou	12352	12.04
						55	Chongqing, Added 2011	1294	1.26
T2	1=URBAN SITE(U) 2=RURAL SITE(R)	Num		8	1.66737509	1-2		102575	100.00
T3	U:1-2=CITY NUM/R:1-4=COUNTY NUM	Num		8	2.18181818	1-4		102575	100.00
T4	U:1-2,5-6,9-10=URB 3-4,7-8,11-12=SUB/R:1,5,9=TWN 2-4,6-8,10-12=VIL	Num		8	2.80660005	1-9		102575	100.00
T5	HOUSEHOLD NUMBER	Num		8	23.1513819	1-152		102575	100.00

Obs	warning	Variable Name	VAR Format	Frequency Category
1	NO OCCURANCES FOR FORMAT CATEGORY:		YR	1989
2	NO OCCURANCES FOR FORMAT CATEGORY:		YR	Missing
