# Supplementary material for: Physical activity, entertainment screen time, and dietary fat profiles and concurrent weight status among school-attending Chinese children and adolescents: CHNS 2004–2011
Source: Front Public Health. 2026 Sep 28;14:1945546. doi: 10.3389/fpubh.2026.1945546 (PMC13654989; doi:10.3389/fpubh.2026.1945546)
Supplement: Supplementary file 3 [file Data_Sheet_2.ZIP › URBAN_11.pdf]

DATA SET: URBAN_11 LABEL: DATE CREATED: 02MAY18:09:51:50
Number of Observations: 2207
Number of Variables: 20
Organization of file: 1 Obs per Community/Year (COMMID/WAVE)

Variable Name	Variable Label (VAR)	VAR Type	VAR Format	VAR Length	Mean	Range of Values	Frequency Category	Frequency	Percent
COMMID	COMMUNITY ID	Num		8	378229.862	111101-552304		2207	100.00
STRATUM	1-city 2-suburban 3-town or county capital city 4-rural village	Num	STRAT	8	2.94154961	1	Urban Neighborhood	415	18.80
						2	Suburban Village	359	16.27
						3	County Town Neighborhood	373	16.90
						4	Rural Village	1060	48.03
WAVE	Survey Year	Num	YR	8	2002.69687	1989	1989	190	8.61
						1991	1991	190	8.61
						1993	1993	188	8.52
						1997	1997	192	8.70
						2000	2000	217	9.83
						2004	2004	216	9.79
						2006	2006	218	9.88
						2009	2009	218	9.88
						2011	2011	290	13.14
						2015	2015	288	13.05
INDEX	Urbanization Index	Num		8	60.5704683	14.26-106.50		2207	100.00
COMM	Communications Component Score	Num		8	4.97033149	0-9.90		1629	73.81
DENC	Community Population Density Category	Num		8	5.96148618	0-10		2207	100.00
DIV	Diversity Score	Num		8	4.81989126	1-9.50		2207	100.00
ECON	Economic Component Score	Num		8	5.41853194	0-10		2207	100.00

DATA SET: URBAN_11 LABEL: DATE CREATED: 02MAY18:09:51:50
Number of Observations: 2207
Number of Variables: 20
Organization of file: 1 Obs per Community/Year (COMMID/WAVE)

Variable Name	Variable Label (VAR)	VAR Type	VAR Format	VAR Length	Mean	Range of Values	Frequency Category	Frequency	Percent
HEALTH	Quality of Health Score	Num		8	5.62614409	0-10		2207	100.00
HOUSE	Housing Component Score	Num		8	6.21353881	0-10		2207	100.00
MARKET	Market Component Score	Num		8	4.98470775	0-10		2207	100.00
SOC	Social Services Score	Num		8	2.3436792	0-10		2207	100.00
TRANS	Transportation Component Score	Num		8	5.40930373	0-10		2207	100.00
EDC	Community Education Category	Num		8	3.46437698	0.48-9.52		2207	100.00
MART	Modern Markets Component Score	Num		8	4.46782963	0-10		2207	100.00
SANI	Sanitation Score	Num		8	6.31554146	0-10		2207	100.00
T1	PROVINCE	Num	PROV	8	37.6361577	11	Beijing, Added 2011	48	2.17
						21	Liaoning, Missed 1997	221	10.01
						23	Heilongjiang, Added 1997	168	7.61
						31	Shanghai, Added 2011	48	2.17
						32	Jiangsu	234	10.60
						37	Shandong	238	10.78
						41	Henan	239	10.83
						42	Hubei	240	10.87
						43	Hunan	243	11.01
						45	Guangxi	240	10.87
						52	Guizhou	240	10.87
						55	Chongqing, Added 2011	48	2.17
T2	1=URBAN SITE(U) 2=RURAL SITE(R)	Num	HH_REG	8	1.64929769	1	Urban	774	35.07

DATA SET: URBAN_11 LABEL: DATE CREATED: 02MAY18:09:51:50
Number of Observations: 2207
Number of Variables: 20
Organization of file: 1 Obs per Community/Year (COMMID/WAVE)

Variable Name	Variable Label (VAR)	VAR Type	VAR Format	VAR Length	Mean	Range of Values	Frequency Category	Frequency	Percent
						2	Rural	1433	64.93
T3	U:1-2=CITY NUM/R:1-4=COUNTY NUM	Num		8	2.16221115	1-4		2207	100.00
T4	U:12569=URB 3478=SUB/R:15=TWN 2-4,6-8=VIL	Num		8	2.76619846	1-9		2207	100.00

Obs	warning	Variable Name	VAR Format	Frequency Category
1	NO OCCURANCES FOR FORMAT CATEGORY:		HH_REG	Missing
2	NO OCCURANCES FOR FORMAT CATEGORY:		PROV	Shanxi, Added 2015
3	NO OCCURANCES FOR FORMAT CATEGORY:		PROV	Yunnan, Added 2015
4	NO OCCURANCES FOR FORMAT CATEGORY:		PROV	Zhejiang, Added 2015
5	NO OCCURANCES FOR FORMAT CATEGORY:		YR	Missing
