# Supplementary material for: Physical activity, entertainment screen time, and dietary fat profiles and concurrent weight status among school-attending Chinese children and adolescents: CHNS 2004–2011
Source: Front Public Health. 2026 Sep 28;14:1945546. doi: 10.3389/fpubh.2026.1945546 (PMC13654989; doi:10.3389/fpubh.2026.1945546)
Supplement: Supplementary file 4 [file Data_Sheet_3.ZIP › farmh_12.pdf]

DATA SET: farmh_12 LABEL: CHNS Household Farming File 1991-2015 DATE CREATED: 01MAY18:15:33:37
Number of Observations: 16529
Number of Variables: 23
Organization of file: 1 Observation Household/Year (HHID/WAVE)

Variable Name	Variable Label (VAR)	VAR Type	VAR Format	VAR Length	Mean	Range of Values	Frequency Category	Frequency	Percent
HHID	HOUSEHOLD ID	Num		8	388082730	111101169 to 552304161		16529	100.00
COMMID	COMMUNITY ID: T1-T4	Num		8	388082.71	111101-552304		16529	100.00
WAVE	SURVEY YEAR	Num	YR	8	2002.44189	1991	1991	2031	12.29
						1993	1993	1894	11.46
						1997	1997	1853	11.21
						2000	2000	1823	11.03
						2004	2004	1945	11.77
						2006	2006	1857	11.23
						2009	2009	1777	10.75
						2011	2011	1923	11.63
						2015	2015	1426	8.63
E1	ANY MEMBER FARMS FOR REGULAR WAGES?	Num	YESNOUNK	8	0.03122519	.	Missing	8971	54.27
						0	No	7322	44.30
						1	Yes	236	1.43
E2	ANY MEM WORKS ON COLLCTV/STATE/HH FARM?	Num	YESNOUNK	8	0.98928571	.	Missing	8969	54.26
						0	No	81	0.49
						1	Yes	7479	45.25
E2B	DID YOUR HOUSEHOLD ENGAGE IN FARMING LYR	Num	FLAG	8	1.02867384	.	Missing	7601	45.99
						1	Yes	8896	53.82
						1-9	9	32	0.19
E11A	HH FARM OPERATED > ONE YEAR?	Num	YESNOUNK	8	1.02163917	.	Missing	12832	77.63
						0	No	64	0.39

DATA SET: farmh_12 LABEL: CHNS Household Farming File 1991-2015 DATE CREATED: 01MAY18:15:33:37
Number of Observations: 16529
Number of Variables: 23
Organization of file: 1 Observation Household/Year (HHID/WAVE)

Variable Name	Variable Label (VAR)	VAR Type	VAR Format	VAR Length	Mean	Range of Values	Frequency Category	Frequency	Percent
						1	Yes	3615	21.87
						9	Unknown (9)	18	0.11
E11B	MONTHS FARM OPERATED < ONE YEAR?	Num		8	0.83018868	-9-12		53	0.32
E11C	IS HH SPECIALIZED FARMING HOUSEHOLD?	Num	YESNOUNK	8	0.10453264	.	Missing	7307	44.21
						0	No	8258	49.96
						1	Yes	964	5.83
E11D	# OF MU OF LAND CULTIVATED LAST YEAR	Num		8	5.28125301	-99-999		14285	86.42
E11E	YEAR LAND LAST REDISTRIBUTED (YYYY)	Num		8	-323.91262	-9999-1998		3605	21.81
E12	YUAN SPENT RAISING CROPS LAST YEAR	Num		8	1047.55781	-999-99999		16190	97.95
E12_CLN	E12 OUTLYING VALUE - REVISED	Num	FLAG	8	1	.	Missing	16522	99.96
						1	Yes	7	0.04
E14A	TOTAL INCOME FROM CROPS LAST YEAR	Num		8	4608.45558	-9999-999999		10479	63.40
E14A_CLN	E14A OUTLYING VALUE - REVISED	Num	FLAG	8	1	.	Missing	16522	99.96
						1	Yes	7	0.04
E16A	VALUE OF CROPS CONSUMED LAST YEAR	Num		8	475.842054	-9999-99999		10320	62.44
E16A_CLN	E16A OUTLYING VALUE - REVISED	Num	FLAG	8	1	.	Missing	16527	99.99
						1	Yes	2	0.01
E16A1	HH CONSUME SOME OF THE CROPS GROWN	Num	YESNOUNK	8	0.84474241	.	Missing	15112	91.43
						0	No	244	1.48
						1	Yes	1170	7.08
						9	Unknown (9)	3	0.02

DATA SET: farmh_12 LABEL: CHNS Household Farming File 1991-2015 DATE CREATED: 01MAY18:15:33:37
Number of Observations: 16529
Number of Variables: 23
Organization of file: 1 Observation Household/Year (HHID/WAVE)

Variable Name	Variable Label (VAR)	VAR Type	VAR Format	VAR Length	Mean	Range of Values	Frequency Category	Frequency	Percent
T1	PROVINCE	Num	PROV	8	38.5957408	11	Beijing, Added 2011	66	0.40
						21	Liaoning, Missed 1997	1376	8.32
						23	Heilongjiang, Added 1997	1622	9.81
						31	Shanghai, Added 2011	8	0.05
						32	Jiangsu	1929	11.67
						37	Shandong	1482	8.97
						41	Henan	2358	14.27
						42	Hubei	1839	11.13
						43	Hunan	1162	7.03
						45	Guangxi	2040	12.34
						52	Guizhou	2352	14.23
						55	Chongqing, Added 2011	295	1.78
T2	1=URBAN SITE(U) 2=RURAL SITE(R)	Num		8	1.87924254	1-2		16529	100.00
T3	U:1-2=CITY NUM/R:1-4=COUNTY NUM	Num		8	2.42954807	1-4		16529	100.00
T4	U:1-2,5-6,9-10=URB 3-4,7-8,11-12=SUB/R:1,5,9=TWN 2-4,6-8,10-12=VIL	Num		8	3.10424103	1-8		16529	100.00
T5	HOUSEHOLD NUMBER	Num		8	20.7056688	1-176		16529	100.00

Obs	warning	Variable Name	VAR Format	Frequency Category
1	NO OCCURANCES FOR FORMAT CATEGORY:		PROV	Shanxi, Added 2015
2	NO OCCURANCES FOR FORMAT CATEGORY:		PROV	Yunnan, Added 2015
3	NO OCCURANCES FOR FORMAT CATEGORY:		PROV	Zhejiang, Added 2015
4	NO OCCURANCES FOR FORMAT CATEGORY:		YESNOUNK	Unknown (-9)
5	NO OCCURANCES FOR FORMAT CATEGORY:		YR	1989
6	NO OCCURANCES FOR FORMAT CATEGORY:		YR	Missing
