# Supplementary material for: Physical activity, entertainment screen time, and dietary fat profiles and concurrent weight status among school-attending Chinese children and adolescents: CHNS 2004–2011
Source: Front Public Health. 2026 Sep 28;14:1945546. doi: 10.3389/fpubh.2026.1945546 (PMC13654989; doi:10.3389/fpubh.2026.1945546)
Supplement: Supplementary file 4 [file Data_Sheet_3.ZIP › asset_12.pdf]

Number of Observations: 44521
Number of Variables: 274
Organization of file: 1 Observation Household/Year (HHID/WAVE)

Variable Name	Variable Label (VAR)	VAR Type	VAR Format	VAR Length	Mean	Range of Values	Frequency Category	Frequency	Percent
HHID	HOUSEHOLD ID	Num		8	379694342	111101001 to 552304161		44521	100.00
COMMID	COMMUNITY ID: T1-T4	Num		8	379694.315	111101-552304		44521	100.00
WAVE	SURVEY YEAR	Num	YR	8	2002.94946	1989	1989	3795	8.52
						1991	1991	3619	8.13
						1993	1993	3456	7.76
						1997	1997	3875	8.70
						2000	2000	4396	9.87
						2004	2004	4387	9.85
						2006	2006	4467	10.03
						2009	2009	4517	10.15
						2011	2011	5923	13.30
						2015	2015	6086	13.67
FM	0 FROM HOUSEHOLD INTERVIEW, 1 FROM PHONE INTERVIEW	Num		8	0.03384818	0-1		6086	13.67
L1	HOW HH OBTAINS DRINKING WATER	Num	WATER	8	1.82356284	.	Missing	528	1.19
						1	In-House Tap Water	25392	57.03
						2	In-Yard Tap Water	6949	15.61
						3	In-Yard Well	8605	19.33
						4	Bottle water	116	0.26
						5	Other Place	2931	6.58
L1A	FILTER WATER BEFORE DRINKING	Num	YESNOUNK	8	0.14103437	.	Missing	38643	86.80
						0	No	5049	11.34
						1	Yes	829	1.86
L1B	OTHER SOURCE OF WATER TO COOK OR WASH FOOD	Num	YESNOUNK	8	0.09118748	.	Missing	38643	86.80

Variable Name	Variable Label (VAR)	VAR Type	VAR Format	VAR Length	Mean	Range of Values	Frequency Category	Frequency	Percent
						0	No	5342	12.00
						1	Yes	536	1.20
L1C	HOW HH OBTAIN WATER TO COOK OR WASH FOOD	Num	WATER	8	1.48603352	.	Missing	43984	98.79
						1	In-House Tap Water	391	0.88
						2	In-Yard Tap Water	69	0.15
						3	In-Yard Well	52	0.12
						4	Bottle water	12	0.03
						5	Other Place	13	0.03
L2	# MINUTES WALK TO H2O OUTLET FROM HOUSE	Num		8	11.9413399	-99-999		3239	7.28
L2A	HOW LONG TO WALK TO ANOTHER PLACE TO GET WATER	Num		8	25.8461538	1-90		13	0.03
L3	MAJOR SOURCE OF DRINKING WATER	Num	H20_SRC	8	3.32126243	.	Missing	669	1.50
						1	Ground Water (> 5 meters)	15324	34.42
						2	Open Well (> 5 meters)	2674	6.01
						3	Creek, Spring, River, Lake	2732	6.14
						4	Ice/Snow	46	0.10
						5	Water Plant	22600	50.76
						6	Bottle Water	120	0.27
						7	Other	166	0.37
						9	Unknown	190	0.43
L3A	WHAT SOURCE OF THIS WATER	Num	H20_SRC	8	3.61080074	.	Missing	43984	98.79
						1	Ground Water (> 5 meters)	159	0.36
						2	Open Well (> 5 meters)	21	0.05
						3	Creek, Spring, River, Lake	50	0.11

Variable Name	Variable Label (VAR)	VAR Type	VAR Format	VAR Length	Mean	Range of Values	Frequency Category	Frequency	Percent
						4	Ice/Snow	1	0.00
						5	Water Plant	280	0.63
						6	Bottle Water	14	0.03
						7	Other	4	0.01
						9	Unknown	8	0.02
L4	DOES HH PAY FOR DRINKING WATER?	Num	YESNOUNK	8	0.63675409	.	Missing	749	1.68
						0	No	15900	35.71
						1	Yes	27872	62.60
L5	TYPE OF TOILET FACILITIES IN HH	Num	TOLIET	8	3.2389292	.	Missing	554	1.24
						0	No Bathroom	727	1.63
						1	Flush, In-House	17747	39.86
						2	No Flush, In-House	1539	3.46
						3	Flush, Outside House, Public Restroom	1453	3.26
						4	No Flush, Outside House, Public Restroom	3836	8.62
						5	Cement Openpit	11228	25.22
						6	Earth Openpit	6879	15.45
						8	Other	558	1.25
L6	GENERAL COND. NEAR HOUSE-EXCRETA REMOVAL	Num	MESS	8	1.41648229	.	Missing	680	1.53
						1	No Excreta	30990	69.61
						2	Very Little Excreta	7768	17.45
						3	Some Excreta	4758	10.69
						4	Much Excreta	325	0.73
L7	TYPE OF LIGHTING GENERALLY USED BY HH	Num	LIGHT	8	1.02102915	.	Missing	582	1.31

Variable Name	Variable Label (VAR)	VAR Type	VAR Format	VAR Length	Mean	Range of Values	Frequency Category	Frequency	Percent
						1	Electric	43288	97.23
						2	Kerosene	510	1.15
						3	Oil	50	0.11
						4	Candle	50	0.11
						5	Other	41	0.09
L8_1	TYPE OF FUEL NORMALLY USED FOR COOKING	Num	COOK	8	3.50018184	.	Missing	527	1.18
						1	Coal	12156	27.30
						2	Electricity	6667	14.97
						3	Kerosene	144	0.32
						4	Liquified Petroleum Gas	10258	23.04
						5	Natural Gas	3902	8.76
						6	Wood, Sticks/Straw, Etc.	9668	21.72
						7	Charcoal	77	0.17
						8	Other	1122	2.52
L8_2	TYPE OF FUEAL ALSO USED FOR COOKING	Num	COOK	8	2.96369598	.	Missing	18904	42.46
						0-8	0	1474	3.31
						1	Coal	3811	8.56
						2	Electricity	9016	20.25
						3	Kerosene	161	0.36
						4	Liquified Petroleum Gas	6563	14.74
						5	Natural Gas	710	1.59
						6	Wood, Sticks/Straw, Etc.	3592	8.07
						7	Charcoal	79	0.18
						8	Other	211	0.47
L9	RENT, OWN OR LIVE FOR FREE IN DWELLING?	Num	RENT	8	3.73427963	.	Missing	16834	37.81
						0-9	0	7	0.02

Variable Name	Variable Label (VAR)	VAR Type	VAR Format	VAR Length	Mean	Range of Values	Frequency Category	Frequency	Percent
						0-9	9	3	0.01
						1	Rent from the state	1629	3.66
						2	Rent from the work unit	1646	3.70
						3	Rent from an individual	333	0.75
						4	Own	23363	52.48
						5	Stay Free	244	0.55
						6	Part Ownership/Partial property right	462	1.04
L10	IF RENT, AMT YUAN PAID MONTHLY IN RENT	Num		8	5.47352609	-999-8008		3868	8.69
L11	IF STAY FOR FREE, ESTIMATED MONTHLY RENT	Num		8	5.06368767	-999-8881		26754	60.09
L11A	DID PERSON MOVE INTO NEW HOUSE TWO YEARS	Num	YESNOUNK	8	0.13066023	.	Missing	25043	56.25
						0	No	16933	38.03
						1	Yes	2545	5.72
L12	AGE OF HOUSE/APARTMENT BUILDING	Num		8	5.85131834	-9-99		11871	26.66
L12A	MONEY SPENT ON INTERIOR DECORATIONS	Num		8	15954.3329	-9999-100000		718	1.61
L13	MATERIALS USED HOUSE/APARTMENT ROOF	Num	ROOF	8	2.25578321	.	Missing	32417	72.81
						1	Concrete	4853	10.90
						2	Straw or tree branches	404	0.91
						3	Tile	6181	13.88
						4	Huijiao	230	0.52
						5	Other	436	0.98
L14	MATERIALS USED HOUSE/APARTMENT FLOOR	Num	FLOOR	8	1.88259109	.	Missing	32418	72.82
						1	Concrete	7362	16.54

Variable Name	Variable Label (VAR)	VAR Type	VAR Format	VAR Length	Mean	Range of Values	Frequency Category	Frequency	Percent
						2	Brick	1811	4.07
						3	Earth	2102	4.72
						4	Wood	192	0.43
						5	Floor Board, wood	76	0.17
						6	Floor Board, wood, man-made	61	0.14
						7	Floor Board, plastic	11	0.02
						8	Other	488	1.10
L15	MATERIALS USED HOUSE/APARTMENT WALLS	Num	WALLS	8	2.09990076	.	Missing	32429	72.84
						1	Concrete	2005	4.50
						2	Brick	8218	18.46
						3	Earth	1003	2.25
						4	Wood	392	0.88
						5	Other	470	1.06
						6	Branches (1989 Only)	4	0.01
L16	TOTAL SIZE OF HH DWELLING UNIT (SQ. M)	Num		8	111.753399	-99-999		28362	63.70
L17	# ROOMS HH OCCUPIES IN HOUSE/APT-NO BATH	Num		8	4.49748334	-9-70		28212	63.37
L18	COST OF REPLACING HOUSE/APARTMENT	Num		8	118999.07	-999999-9999999		26690	59.95
L18A	HAVE OTHER HOUSES/APARTMENTS	Num	YESNOUNK	8	0.09101735	.	Missing	38643	86.80
						0	No	5343	12.00
						1	Yes	535	1.20
L18B	TOTAL USABLE AREA OF ALL OTHER HOUSES/APARTMENTS	Num		8	129.973832	7-2000		535	1.20
L18C	COST OF ALL OTHER HOUSES/APARTMENTS	Num		8	273.748466	1-9999		489	1.10

Variable Name	Variable Label (VAR)	VAR Type	VAR Format	VAR Length	Mean	Range of Values	Frequency Category	Frequency	Percent
L18P	DWELLING WORTH	Num		8	239.255639	1-9999		133	0.30
L19	ANY HH MEMBER OWN TRICYCLE?	Num	YESNOUNK	8	0.1019339	.	Missing	620	1.39
						0	No	39642	89.04
						1	Yes	4232	9.51
						9	Unknown (9)	27	0.06
L20	# TRICYCLES OWNED	Num		8	1.06793349	0-4		4210	9.46
L21	# TRICYCLES PURCHASED IN LAST 12 MONTHS	Num		8	0.27272727	0-1		110	0.25
L22	TOTAL VALUE OF TRICYCLES BOUGHT LAST 12-MON	Num		8	9116.19495	-99999-9999999		4078	9.16
L23	ANY HH MEMBER OWN BICYCLE?	Num	YESNOUNK	8	0.62029631	.	Missing	513	1.15
						0	No	16926	38.02
						1	Yes	27055	60.77
						9	Unknown (9)	27	0.06
L24	# BICYCLES OWNED	Num		8	1.67888852	0-9		26919	60.46
L25	# BICYCLES PURCHASED IN LAST 12 MONTHS	Num		8	0.2628087	0-5		2713	6.09
L26	TOTAL VALUE OF BIKES BOUGHT LAST 12 MONTHS	Num		8	3256.21253	-99999-9999999		24476	54.98
L27	ANY HH MEMBER OWN MOTORCYCLE?	Num	YESNOUNK	8	0.19813757	.	Missing	385	0.86
						0	No	35639	80.05
						1	Yes	8466	19.02
						9	Unknown (9)	31	0.07
L28	# MOTORCYCLES OWNED	Num		8	1.13137632	0-9		8312	18.67
L29	# MOTORCYCLES PURCHASED IN LAST 12 MON	Num		8	0.30434783	0-2		69	0.15
L30	TOTAL VALUE OF MOTORCYC. BOUGHT LAST 12 MON	Num		8	6414.30155	-99999-9999999		8310	18.67

Variable Name	Variable Label (VAR)	VAR Type	VAR Format	VAR Length	Mean	Range of Values	Frequency Category	Frequency	Percent
L31	ANY HH MEMBER OWN CAR?	Num	YESNOUNK	8	0.06829313	.	Missing	417	0.94
						0	No	41340	92.86
						1	Yes	2733	6.14
						9	Unknown (9)	31	0.07
L32	# CARS OWNED	Num		8	1.10217881	0-9		2662	5.98
L33	# CARS PURCHASED IN LAST 12 MONTHS	Num		8	0.3	0-1		20	0.04
L34	TOTAL VALUE OF CARS BOUGHT LAST 12 MONTHS	Num		8	125543.548	-99999-9999999		2585	5.81
L35	DOES ANY HH MEMBER FARM?	Num	YESNOUNK	8	0.6003861	.	Missing	37269	83.71
						0	No	2898	6.51
						1	Yes	4354	9.78
L36	TOTAL AMT LAND FARMED BY HH 1989 (MU)	Num		8	2.9869322	-99-63		5900	13.25
L37	ANY HH MEMBER OWN TRACTOR?	Num	YESNOUNK	8	0.04142525	.	Missing	683	1.53
						0	No	42262	94.93
						1	Yes	1546	3.47
						9	Unknown (9)	30	0.07
L38	# TRACTORS OWNED	Num		8	1.04619388	0-4		1537	3.45
L39	# TRACTORS PURCHASED LAST 12 MONTHS	Num		8	0.32142857	0-1		28	0.06
L40	VALUE OF TRACTORS BOUGHT LAST 12 MONTHS	Num		8	9329.48904	-9999-250000		1505	3.38
L41	ANY HH MEMBER OWN WALKING TRACTORS?	Num	YESNOUNK	8	0.03672948	.	Missing	687	1.54
						0	No	42464	95.38
						1	Yes	1340	3.01
						9	Unknown (9)	30	0.07

Variable Name	Variable Label (VAR)	VAR Type	VAR Format	VAR Length	Mean	Range of Values	Frequency Category	Frequency	Percent
L42	# WALKING TRACTORS OWNED	Num		8	1.02947846	0-9		1323	2.97
L43	# WALKING TRACTORS BOUGHT LAST 12 MON.	Num		8	0.28421053	0-1		95	0.21
L44	VALUE OF WALKING TRACTORS BOUGHT LAST 12 MON	Num		8	3416.083	-9999-70000		1265	2.84
L45	ANY HH MEMBER OWN ANIMAL CART?	Num	YESNOUNK	8	0.12094368	.	Missing	33797	75.91
						0	No	9427	21.17
						1	Yes	1297	2.91
L45A	DOES HH OWN DRAFT ANIMALS?	Num	YESNOUNK	8	0.15844119	.	Missing	37490	84.21
						0	No	5917	13.29
						1	Yes	1114	2.50
L46	# ANIMAL CARTS OWNED	Num		8	1.01945525	0-4		1285	2.89
L46A	NUMBER OF DRAFT ANIMALS OWNED	Num		8	1.19034355	0-5		1077	2.42
L47	# ANIMAL CARTS PURCHASED LAST 12 MONTHS	Num		8	0.10599078	0-2		434	0.97
L48	COST ANIMAL CARTS BOUGHT LAST 12 MONTHS	Num		8	-541.95977	-9999-6000		870	1.95
L48A	VALUE OF DRAFT ANIMALS OWNED	Num		8	198.500451	-9999-15000		1109	2.49
L49	ANY HH MEMBER OWN IRRIGATION EQUIPMENT?	Num	YESNOUNK	8	0.03528982	.	Missing	684	1.54
						0	No	42522	95.51
						1	Yes	1286	2.89
						9	Unknown (9)	29	0.07
L50	# IRRIGATION EQUIPMENT OWNED	Num		8	1.05246423	0-9		1258	2.83
L51	# IRRIG. EQUIP. BOUGHT LAST 12 MONTHS	Num		8	0.1	0-1		70	0.16
L52	VALUE OF IRRIG. EQUIP. BOUGHT LAST 12 MON	Num		8	524.953566	-9999-40000		1206	2.71

Variable Name	Variable Label (VAR)	VAR Type	VAR Format	VAR Length	Mean	Range of Values	Frequency Category	Frequency	Percent
L53	ANY HH MEMBER OWN POWER THRESHER?	Num	YESNOUNK	8	0.02352109	.	Missing	688	1.55
						0	No	43026	96.64
						1	Yes	779	1.75
						9	Unknown (9)	28	0.06
L54	# POWER THRESHERS OWNED	Num		8	1.02649007	0-4		755	1.70
L55	# POWER THRESHERS BOUGHT LAST 12 MON	Num		8	0.07692308	0-1		39	0.09
L56	VALUE OF POWER THRESH. BOUGHT LAST 12 MON	Num		8	1296.93122	-9999-138000		727	1.63
L57	ANY HH MEMBER OWN WATER PUMP?	Num	YESNOUNK	8	0.08226086	.	Missing	697	1.57
						0	No	40451	90.86
						1	Yes	3344	7.51
						9	Unknown (9)	29	0.07
L57B	OTHER FARM MACHINERY OWNED? SPECIFY	Num	YESNOUNK	8	0.02563316	.	Missing	28214	63.37
						0	No	16193	36.37
						1	Yes	76	0.17
						9	Unknown (9)	38	0.09
L58	# WATER PUMPS OWNED	Num		8	1.0513054	0-9		3294	7.40
L58B	OTHER FARM MACHINERY: # OWNED	Num		8	1.09333333	1-3		75	0.17
L59	# WATER PUMPS BOUGHT LAST 12 MONTHS	Num		8	0.24096386	0-1		83	0.19
L60	VALUE OF WATER PUMPS BOUGHT LAST 12 MONTHS	Num		8	130.228492	-9999-47000		3243	7.28
L60B	OTHER FARM MACHINERY: TOTAL VALUE	Num		8	7599.44	-9999-99999		75	0.17
L61	ANY HH MEMBER OWN PIGS?	Num	YESNOUNK	8	0.81591412	.	Missing	12009	26.97
						0	No	5985	13.44

Variable Name	Variable Label (VAR)	VAR Type	VAR Format	VAR Length	Mean	Range of Values	Frequency Category	Frequency	Percent
						1	Yes	26527	59.58
L62	ANY HH MEMBER OWN SHEEP/GOATS?	Num	YESNOUNK	8	0.68814592	.	Missing	12009	26.97
						0	No	10139	22.77
						1	Yes	22373	50.25
L63	ANY HH MEMBER OWN POULTRY?	Num	YESNOUNK	8	0.82686393	.	Missing	12009	26.97
						0	No	5629	12.64
						1	Yes	26883	60.38
L64	ANY HH MEMBER OWN BUFFALOES/OXEN?	Num	YESNOUNK	8	0.14583898	.	Missing	40832	91.71
						0	No	3151	7.08
						1	Yes	538	1.21
L65	ANY HH MEMBER OWN OTHER DRAFT ANIMALS?	Num	YESNOUNK	8	0.04035753	.	Missing	40829	91.71
						0	No	3543	7.96
						1	Yes	149	0.33
L66	ANY HH MEMBER OWN OTHER LIVESTOCK?	Num	YESNOUNK	8	0.68608514	.	Missing	12009	26.97
						0	No	10206	22.92
						1	Yes	22306	50.10
L67	TOTAL VALUE OF ALL HH LIVESTOCK, IF SOLD	Num		8	-281.22865	-9999-99999		6018	13.52
L68	ANY HH MEMBER OWN FISHING NETS?	Num	YESNOUNK	8	0.01144465	.	Missing	33861	76.06
						0	No	10538	23.67
						1	Yes	122	0.27
L69	ANY HH MEMBER OWN FISHING BOATS?	Num	YESNOUNK	8	0.0048808	.	Missing	33867	76.07
						0	No	10604	23.82
						0-3	3	1	0.00
						1	Yes	49	0.11

Variable Name	Variable Label (VAR)	VAR Type	VAR Format	VAR Length	Mean	Range of Values	Frequency Category	Frequency	Percent
L70	ANY HH MEMBER OWN MARINE ENGINES?	Num	YESNOUNK	8	0.00197183	.	Missing	33871	76.08
						0	No	10629	23.87
						1	Yes	21	0.05
L71	ANY HH MEMBER OWN PRESSURE LAMPS?	Num	YESNOUNK	8	0.00112851	.	Missing	37432	84.08
						0	No	7081	15.90
						1	Yes	8	0.02
L72	ANY HH MEMBER OWN OTHER FISHING EQUIP?	Num	YESNOUNK	8	0.00086625	.	Missing	38749	87.04
						0	No	5767	12.95
						1	Yes	5	0.01
L73	TOTAL VALUE ALL HH FISH EQUIP, IF SOLD	Num		8	-49.220588	-9999-15000		204	0.46
L74	ANY HH MEMBER OWN PROF. COOKING EQUIP?	Num	YESNOUNK	8	0.06870403	.	Missing	739	1.66
						0	No	41110	92.34
						1	Yes	2630	5.91
						9	Unknown (9)	42	0.09
L74A	COOKING EQUIP USED IN HH BUSINESS?	Num		8	0.15853659	0-9		2624	5.89
L75	ANY HH MEMB OWN PROF. CARPENTRY EQUIP?	Num	YESNOUNK	8	0.0216835	.	Missing	755	1.70
						0	No	43105	96.82
						1	Yes	625	1.40
						9	Unknown (9)	36	0.08
L75A	CARPENTRY EQUIP?	Num	YESNOUNK	8	0.4376947	.	Missing	43879	98.56
						0	No	369	0.83
						1	Yes	272	0.61
						9	Unknown (9)	1	0.00

Variable Name	Variable Label (VAR)	VAR Type	VAR Format	VAR Length	Mean	Range of Values	Frequency Category	Frequency	Percent
L76	ANY HH MEMB OWN PROF. BEAUTICIAN EQUIP?	Num	YESNOUNK	8	0.01178997	.	Missing	755	1.70
						0	No	43538	97.79
						1	Yes	192	0.43
						9	Unknown (9)	36	0.08
L76A	BEAUTICIAN'S EQUIP USED IN HH BUSINESS?	Num		8	0.28703704	0-1		324	0.73
L77	ANY HH MEMB OWN PROF. SEWING EQUIP?	Num	YESNOUNK	8	0.02848853	.	Missing	749	1.68
						0	No	42829	96.20
						1	Yes	905	2.03
						9	Unknown (9)	38	0.09
L77A	SEWING EQUIP USED IN HH BUSINESS?	Num	YESNOUNK	8	0.28211587	.	Missing	43727	98.22
						0	No	602	1.35
						1	Yes	188	0.42
						9	Unknown (9)	4	0.01
L78	ANY HH MEMB OWN PROF. WELDING EQUIP?	Num	YESNOUNK	8	0.02090998	.	Missing	762	1.71
						0	No	43140	96.90
						1	Yes	582	1.31
						9	Unknown (9)	37	0.08
L78A	SHOP TOOLS USED IN HH BUSINESS?	Num	YESNOUNK	8	0.37553957	.	Missing	43826	98.44
						0	No	450	1.01
						1	Yes	243	0.55
						9	Unknown (9)	2	0.00
L79	ANY HH MEMB OWN PROF MACHINE SHOP TOOLS?	Num	YESNOUNK	8	0.00460081	.	Missing	40826	91.70
						0	No	3678	8.26
						1	Yes	17	0.04

Variable Name	Variable Label (VAR)	VAR Type	VAR Format	VAR Length	Mean	Range of Values	Frequency Category	Frequency	Percent
L80	ANY HH MEMB OWN OTHER BUS/PROF EQUIP?	Num	YESNOUNK	8	0.03047955	.	Missing	918	2.06
						0	No	42722	95.96
						1	Yes	825	1.85
						9	Unknown (9)	56	0.13
L80A	OTHER PROF EQUIP USED IN HH BUSINESS?	Num		8	0.79115479	0-9		814	1.83
L81	TOTAL VALUE OF ALL HH BUS/PROF EQUIP, IF SOLD	Num		8	3600.08965	-9999-999999		5198	11.68
L82	ANY HH MEMB OWN LIVING ROOM FURNITURE?	Num	YESNOUNK	8	0.97093381	.	Missing	12009	26.97
						0	No	945	2.12
						1	Yes	31567	70.90
L83	# PIECES OF LIVING ROOM FURNITURE OWNED	Num		8	6.57746768	0-70		9746	21.89
L84	# PIECES LIV. RM FURN BOUGHT LAST 12 MON	Num		8	0.39213115	0-25		3050	6.85
L85	TOTL COST LIV RM FURN BOUGHT LAST 12 MON	Num		8	-2195.1437	-9999-10030		6534	14.68
L86	ANY HH MEMBER OWN BEDROOM FURNITURE?	Num	YESNOUNK	8	0.9913878	.	Missing	12009	26.97
						0	No	280	0.63
						1	Yes	32232	72.40
L87	# BEDS/DRESSERS OWNED?	Num		8	5.46739987	0-60		10411	23.38
L88	# BEDS/DRESSERS BOUGHT LAST 12 MONTHS	Num		8	0.2512836	0-15		3311	7.44
L89	TOTL COST BEDRM FURN BOUGHT LAST 12 MON	Num		8	-2141.3902	-9999-21000		6837	15.36
L90	ANY HH MEMBER OWN STEREO/TAPE?	Num	YESNOUNK	8	0.38545428	.	Missing	16829	37.80
						0	No	17026	38.24
						1	Yes	10665	23.95
						9	Unknown (9)	1	0.00

Variable Name	Variable Label (VAR)	VAR Type	VAR Format	VAR Length	Mean	Range of Values	Frequency Category	Frequency	Percent
L91	# STEREO/TAPES OWNED	Num		8	1.11113209	0-7		10591	23.79
L92	# STEREO/TAPES BOUGHT LAST 12 MONTHS	Num		8	0.11338673	0-6		8740	19.63
L93	TOT COST ALL STER/TAPE BOUGHT LAST 12 MO	Num		8	161.066058	-9999-32001		9416	21.15
L94	WHOSE DECISION TO BUY STEREO/TAPE?	Num	DECIDE	8	2.47261761	.	Missing	40376	90.69
						1	Husband	1182	2.65
						2	Wife	266	0.60
						3	Husband and Wife	2253	5.06
						4	Other	444	1.00
L94A	# OF RADIOS REC'D AS GIFTS LAST YEAR	Num		8	0.03246466	0-2		4528	10.17
L95	ANY HH MEMBER OWN VCR?	Num	YESNOUNK	8	0.04691851	.	Missing	6455	14.50
						0	No	36280	81.49
						1	Yes	1786	4.01
L96	# VCRS OWNED	Num		8	1.03225806	0-9		1767	3.97
L97	# VCRS BOUGHT LAST 12 MONTHS	Num		8	0.14211213	0-4		1534	3.45
L98	TOTAL VALUE OF ALL VCRS BOUGHT LAST 12 MO	Num		8	1119.24898	-9999-24800		1723	3.87
L99	WHOSE DECISION TO BUY VCR?	Num	DECIDE	8	2.60542169	.	Missing	44189	99.25
						1	Husband	71	0.16
						2	Wife	17	0.04
						3	Husband and Wife	216	0.49
						4	Other	28	0.06
L99A	# OF VCRs REC'D AS GIFTS LAST YEAR	Num		8	0.03846154	0-1		494	1.11
L100	ANY HH MEMBER OWN A BL/WH TV?	Num	YESNOUNK	8	0.3647701	.	Missing	16857	37.86

Variable Name	Variable Label (VAR)	VAR Type	VAR Format	VAR Length	Mean	Range of Values	Frequency Category	Frequency	Percent
						0	No	17573	39.47
						1	Yes	10091	22.67
L101	# BL/WH TVS OWNED	Num		8	1.01701702	0-3		9990	22.44
L102	# BL/WH TVS BOUGHT LAST 12 MONTHS	Num		8	0.09081322	0-3		8534	19.17
L103	TOTAL COST ALL BL/WH TVS BOUGHT 12 MON	Num		8	256.415286	-9999-11400		8387	18.84
L104	WHOSE DECISION TO BUY BL/WH TV?	Num	DECIDE	8	2.47361535	.	Missing	39935	89.70
						1	Husband	1181	2.65
						2	Wife	355	0.80
						3	Husband and Wife	2747	6.17
						4	Other	303	0.68
L104A	# OF B/W TVs REC'D AS GIFTS LAST YEAR	Num		8	0.02746092	0-3		4734	10.63
L105	ANY HH MEMBER OWN A COLOR TV?	Num	YESNOUNK	8	0.7065294	.	Missing	520	1.17
						0	No	12937	29.06
						1	Yes	31061	69.77
						9	Unknown (9)	3	0.01
L106	# COLOR TVS OWNED	Num		8	1.28272184	0-20		30935	69.48
L107	# COLOR TVS BOUGHT LAST 12 MONTHS	Num		8	0.12944503	0-11		28290	63.54
L108	TOTAL VALUE OF ALL COLOR TVS BOUGHT 12 MON	Num		8	1799.77937	-9999-99999		24593	55.24
L109	WHOSE DECISION TO BUY COLOR TV?	Num	DECIDE	8	2.62380038	.	Missing	41916	94.15
						1	Husband	505	1.13
						2	Wife	169	0.38
						3	Husband and Wife	1732	3.89
						4	Other	199	0.45

Variable Name	Variable Label (VAR)	VAR Type	VAR Format	VAR Length	Mean	Range of Values	Frequency Category	Frequency	Percent
L109A	# OF COLOR TVs REC'D AS GIFTS LAST YEAR	Num		8	0.04714715	0-9		3330	7.48
L110	ANY HH MEMBER OWN WASHING MACHINE?	Num	YESNOUNK	8	0.6009478	.	Missing	419	0.94
						0	No	17647	39.64
						1	Yes	26449	59.41
						9	Unknown (9)	6	0.01
L111	# WASHING MACHINES OWNED	Num		8	1.04428479	0-10		26307	59.09
L112	# WASHING MACHINES PURCHASED LAST 12 MON	Num		8	0.08454676	0-5		23762	53.37
L113	TOTAL VALUE OF WASH MACH BOUGHT LAST 12 MON	Num		8	524.555355	-9999-99999		19944	44.80
L114	WHOSE DECISION TO BUY WASHING MACHINE?	Num	DECIDE	8	2.62534267	.	Missing	41238	92.63
						1	Husband	454	1.02
						2	Wife	508	1.14
						3	Husband and Wife	2135	4.80
						4	Other	186	0.42
L114A	WASHING MACHINES REC'D AS GIFTS LAST YR	Num		8	0.04198473	0-1		3668	8.24
L115	ANY HH MEMBER OWN REFRIGERATOR?	Num	YESNOUNK	8	0.48980425	.	Missing	434	0.97
						0	No	22549	50.65
						1	Yes	21531	48.36
						9	Unknown (9)	7	0.02
L116	# REFRIGERATORS OWNED	Num		8	0.91414816	0-13		24717	55.52
L117	# REFRIG PURCHASED LAST 12 MONTHS	Num		8	0.08256442	0-9		22976	51.61
L118	TOTAL VALUE OF REFRIG BOUGHT LAST 12 MON	Num		8	1304.89299	-9999-99999		15700	35.26
L119	WHOSE DECISON TO BUY REFRIGERATOR?	Num	DECIDE	8	2.69864699	.	Missing	42895	96.35

Variable Name	Variable Label (VAR)	VAR Type	VAR Format	VAR Length	Mean	Range of Values	Frequency Category	Frequency	Percent
						1	Husband	228	0.51
						2	Wife	124	0.28
						3	Husband and Wife	1184	2.66
						4	Other	90	0.20
L119A	REFRIGERATORS REC'D AS GIFTS LAST YEAR	Num		8	0.03019971	0-1		2053	4.61
L120	ANY HH MEMBER OWN AIR CONDITIONER?	Num	YESNOUNK	8	0.22017349	.	Missing	483	1.08
						0	No	34390	77.24
						1	Yes	9642	21.66
						9	Unknown (9)	6	0.01
L121	# AIR CONDITIONERS OWNED	Num		8	1.18863976	0-20		13380	30.05
L122	# AIR COND PURCHASED LAST 12 MONTHS	Num		8	0.11944229	0-9		12910	29.00
L123	TOTAL VALUE OF AIR COND BOUGHT LAST 12 MON	Num		8	2872.68053	-9999-99990		6179	13.88
L124	WHOSE DECISION TO BUY AIR CONDITIONER?	Num	DECIDE	8	2.48453608	.	Missing	44424	99.78
						1	Husband	24	0.05
						2	Wife	9	0.02
						3	Husband and Wife	57	0.13
						4	Other	7	0.02
L124A	AIR CONDITIONERS REC'D AS GIFTS LAST YR	Num		8	0.05392157	0-5		204	0.46
L125	ANY HH MEMBER OWN SEWING MACHINE?	Num	YESNOUNK	8	0.36950359	.	Missing	424	0.95
						0	No	27867	62.59
						1	Yes	16222	36.44
						9	Unknown (9)	8	0.02
L126	# SEWING MACHINES OWNED	Num		8	0.93345656	0-10		17853	40.10

Variable Name	Variable Label (VAR)	VAR Type	VAR Format	VAR Length	Mean	Range of Values	Frequency Category	Frequency	Percent
L127	# SEWING MACHINES BOUGHT LAST 12 MON	Num		8	0.03943804	0-9		15873	35.65
L128	TOTAL VALUE OF SEWING MACH BOUGHT LAST 12 MON	Num		8	14.7513694	-9999-18000		13144	29.52
L129	WHOSE DECISION TO BUY SEWING MACHINE?	Num	DECIDE	8	2.51092837	.	Missing	39900	89.62
						1	Husband	768	1.73
						2	Wife	964	2.17
						3	Husband and Wife	2649	5.95
						4	Other	240	0.54
L129A	SEWING MACHINES REC'D AS GIFTS LAST YEAR	Num		8	0.03229256	0-3		4676	10.50
L130	ANY HH MEMBER OWN ELECTRIC FAN?	Num	YESNOUNK	8	0.74309411	.	Missing	392	0.88
						0	No	11377	25.55
						1	Yes	32747	73.55
						9	Unknown (9)	5	0.01
L131	# ELECTRIC FANS OWNED	Num		8	1.96118575	0-20		34240	76.91
L132	# ELECTRIC FANS BOUGHT LAST 12 MONTHS	Num		8	0.13787884	0-9		31049	69.74
L133	TOTAL VALUE OF ELECTRIC FANS BOUGHT 12 MON	Num		8	29.4460423	-9999-99999		25937	58.26
L134	WHOSE DECISION TO BUY ELECTRIC FAN?	Num	DECIDE	8	2.47875595	.	Missing	38637	86.78
						1	Husband	1469	3.30
						2	Wife	468	1.05
						3	Husband and Wife	3608	8.10
						4	Other	339	0.76
L134A	ELECTRIC FANS REC'D AS GIFTS LAST YEAR	Num		8	0.03526291	0-9		6409	14.40
L135	ANY HH MEMBER OWN BIG WALL CLOCK?	Num		8	0.37826368	0-1		10839	24.35

Variable Name	Variable Label (VAR)	VAR Type	VAR Format	VAR Length	Mean	Range of Values	Frequency Category	Frequency	Percent
L136	# BIG WALL CLOCKS OWNED	Num		8	0.66823422	0-5		6592	14.81
L137	# BIG WALL CLOCKS BOUGHT LAST 12 MON	Num		8	0.06371058	0-4		6247	14.03
L138	TOTAL COST BIG WALL CLOCKS LAST 12 MON	Num		8	18.1372822	-999-6000		2870	6.45
L139	WHOSE DECISION TO BUY BIG WALL CLOCK?	Num	DECIDE	8	2.48738218	.	Missing	41232	92.61
						1	Husband	792	1.78
						2	Wife	292	0.66
						3	Husband and Wife	2015	4.53
						4	Other	190	0.43
L139A	# OF WALL CLOCKS REC'D AS GIFTS LAST YR	Num		8	0.05731225	0-3		2530	5.68
L140	ANY HH MEMBER OWN CAMERA?	Num	YESNOUNK	8	0.14356087	.	Missing	470	1.06
						0	No	37839	84.99
						1	Yes	6198	13.92
						9	Unknown (9)	14	0.03
L140A	DOES HH OWN MICROWAVE?	Num	YESNOUNK	8	0.1897841	.	Missing	4270	9.59
						0	No	32708	73.47
						1	Yes	7531	16.92
						9	Unknown (9)	12	0.03
L140B	DOES HH OWN ELECTRIC COOKING POT?	Num	YESNOUNK	8	0.62532582	.	Missing	4238	9.52
						0	No	15125	33.97
						1	Yes	25154	56.50
						9	Unknown (9)	4	0.01
L140C	DOES HH OWN PRESSURE COOKER?	Num	YESNOUNK	8	0.43917275	.	Missing	4243	9.53
						0	No	22645	50.86
						1	Yes	17626	39.59

Variable Name	Variable Label (VAR)	VAR Type	VAR Format	VAR Length	Mean	Range of Values	Frequency Category	Frequency	Percent
						9	Unknown (9)	7	0.02
L140D	DOES HH OWN METAL COOKING TOOLS?	Num	YESNOUNK	8	0.17331245	.	Missing	37499	84.23
						0	No	5805	13.04
						1	Yes	1217	2.73
L140E	COMPUTER: OWNED?	Num	YESNOUNK	8	0.21842921	.	Missing	11302	25.39
						0	No	26011	58.42
						1	Yes	7202	16.18
						9	Unknown (9)	6	0.01
L140F	TELEPHONE: OWNED?	Num	YESNOUNK	8	0.51723827	.	Missing	11281	25.34
						0	No	16103	36.17
						1	Yes	17130	38.48
						9	Unknown (9)	7	0.02
L140G	VCD/DVD: OWNED?	Num	YESNOUNK	8	0.31857085	.	Missing	15077	33.86
						0	No	20112	45.17
						1	Yes	9326	20.95
						9	Unknown (9)	6	0.01
L140H	CELLPHONE: OWNED?	Num	YESNOUNK	8	0.65657209	.	Missing	19392	43.56
						0	No	8686	19.51
						1	Yes	16436	36.92
						9	Unknown (9)	7	0.02
L140I	SATELLITE DISH: OWNED?	Num	YESNOUNK	8	0.11816561	.	Missing	23762	53.37
						0	No	18370	41.26
						1	Yes	2381	5.35
						9	Unknown (9)	8	0.02

Variable Name	Variable Label (VAR)	VAR Type	VAR Format	VAR Length	Mean	Range of Values	Frequency Category	Frequency	Percent
L140J	TABLET COMPUTER: OWNED?	Num	YESNOUNK	8	0.20636271	.	Missing	38643	86.80
						0	No	4761	10.69
						1	Yes	1105	2.48
						9	Unknown (9)	12	0.03
L140K	SMARTPHONE: OWNED?	Num	YESNOUNK	8	0.64392651	.	Missing	38643	86.80
						0	No	2205	4.95
						1	Yes	3659	8.22
						9	Unknown (9)	14	0.03
L140L	EXERCISE BIKES/TREADMILLS: OWNED?	Num	YESNOUNK	8	0.02619939	.	Missing	38643	86.80
						0	No	5772	12.96
						1	Yes	100	0.22
						9	Unknown (9)	6	0.01
L140M	MASSAGE CHAIR: OWNED?	Num	YESNOUNK	8	0.02960191	.	Missing	38643	86.80
						0	No	5752	12.92
						1	Yes	120	0.27
						9	Unknown (9)	6	0.01
L140N	AIR PURIFIER: OWNED?	Num	YESNOUNK	8	0.04253147	.	Missing	38643	86.80
						0	No	5676	12.75
						1	Yes	196	0.44
						9	Unknown (9)	6	0.01
L141	# CAMERAS OWNED	Num		8	0.69830334	0-10		9725	21.84
L141A	NUMBER OF MICROWAVES OWNED	Num		8	1.02480331	0-10		7499	16.84
L141B	NUMBER OF ELECTIC COOKING POTS OWNED	Num		8	1.12116246	0-20		25016	56.19
L141C	NUMBER OF PRESSURE COOKERS OWNED	Num		8	1.16622917	0-10		17524	39.36

Variable Name	Variable Label (VAR)	VAR Type	VAR Format	VAR Length	Mean	Range of Values	Frequency Category	Frequency	Percent
L141D	NUMBER OF METAL COOKING TOOLS OWNED	Num		8	1.8663325	0-9		1197	2.69
L141E	COMPUTER: # OWNED	Num		8	1.18685603	1-20		7182	16.13
L141F	TELEPHONE: # OWNED	Num		8	1.08425512	0-10		16996	38.18
L141G	VCD/DVD: # OWNED	Num		8	1.06653705	1-10		9258	20.79
L141H	CELLPHONE: # OWNED	Num		8	1.77071891	1-20		16386	36.81
L141I	SATELLITE DISH: # OWNED?	Num		8	1.05267594	1-10		2373	5.33
L141J	TABLET COMPUTER: # OWNED	Num		8	1.15294118	1-10		1105	2.48
L141K	SMARTPHONE: # OWNED	Num		8	2.10062893	1-20		3657	8.21
L141L	EXERCISE BIKES/TREADMILLS: # OWNED	Num		8	1.06060606	1-4		99	0.22
L141M	MASSAGE CHAIR: # OWNED	Num		8	1.11666667	1-10		120	0.27
L141N	AIR PURIFIER: # OWNED	Num		8	1.10714286	1-10		196	0.44
L142	# CAMERAS PURCHASED LAST 12 MONTHS	Num		8	0.05498805	0-5		9202	20.67
L142A	NUMBER OF MICROWAVES BOUGHT LAST YEAR	Num		8	0.07181704	0-7		7171	16.11
L142B	# OF ELECTRIC COOK POTS BOUGHT LAST YEAR	Num		8	0.08493115	0-7		22948	51.54
L142C	# OF PRESSURE COOKERS BOUGHT LAST YEAR	Num		8	0.07166953	0-6		15711	35.29
L142D	NUMBER OF COOK TOOLS BOUGHT LAST YEAR	Num		8	0.15718419	0-6		1037	2.33
L142E	COMPUTER: # BOUGHT LAST YEAR	Num		8	0.12950369	0-6		6911	15.52
L142F	TELEPHONE: # BOUGHT LAST YEAR	Num		8	0.06701132	0-9		15281	34.32

Variable Name	Variable Label (VAR)	VAR Type	VAR Format	VAR Length	Mean	Range of Values	Frequency Category	Frequency	Percent
L142G	VCD/DVD: # BOUGHT LAST YEAR	Num		8	0.10204802	0-6		8496	19.08
L142H	CELLPHONE: # BOUGHT LAST YEAR	Num		8	0.19890835	0-6		15756	35.39
L142I	SATELLITE DISH: # BOUGHT LAST YEAR?	Num		8	0.08746736	0-3		2298	5.16
L142J	TABLET COMPUTER: # BOUGHT LAST YEAR	Num		8	0.11887477	0-2		1102	2.48
L142K	SMARTPHONE: # BOUGHT LAST YEAR	Num		8	0.22836802	0-6		3652	8.20
L142L	EXERCISE BIKES/TREADMILLS: # BOUGHT LAST YEAR	Num		8	0.15151515	0-2		99	0.22
L142M	MASSAGE CHAIR: # BOUGHT LAST YEAR	Num		8	0.11764706	0-1		119	0.27
L142N	AIR PURIFIER: # BOUGHT LAST YEAR	Num		8	0.16326531	0-1		196	0.44
L143	TOTAL VALUE OF CAMERAS BOUGHT LAST 12 MON	Num		8	713.261252	-9999-98000		4777	10.73
L143A	VALUE OF MICROWAVES BOUGHT LAST YEAR	Num		8	111.633346	-9999-33000		5242	11.77
L143B	VALUE OF ELEC COOK POTS BOUGHT LAST YEAR	Num		8	-60.352717	-9999-22280		19639	44.11
L143C	VALUE OF PRESSURE COOKERS BOUGHT LAST YR	Num		8	-148.41415	-9999-80110		14217	31.93
L143D	VALUE OF COOK TOOLS BOUGHT LAST YEAR	Num		8	153.301255	-999-4000		1195	2.68
L143E	COMPUTER: VALUE OF ITEMS OWNED	Num		8	2907.35021	-9999-70001		4563	10.25
L143F	TELEPHONE: VALUE OF ITEMS OWNED	Num		8	45.2992239	-9999-99999		14688	32.99
L143G	VCD/DVD: VALUE OF ITEMS OWNED	Num		8	219.128214	-9999-98000		8283	18.60
L143H	CELLPHONE: VALUE OF ITEMS OWNED	Num		8	1216.02239	-9999-99999		13041	29.29
L143I	SATELLITE DISH: VALUE OF ITEMS OWNED?	Num		8	-87.016982	-9999-30000		1531	3.44

Variable Name	Variable Label (VAR)	VAR Type	VAR Format	VAR Length	Mean	Range of Values	Frequency Category	Frequency	Percent
L144	WHOSE DECISION TO BUY CAMERA?	Num	DECIDE	8	2.59061489	.	Missing	43903	98.61
						1	Husband	140	0.31
						2	Wife	39	0.09
						3	Husband and Wife	373	0.84
						4	Other	66	0.15
L144A	PERSON WHO DECIDED TO BUY MICROWAVE OVEN	Num	DECIDE	8	2.625	.	Missing	44481	99.91
						1	Husband	8	0.02
						2	Wife	4	0.01
						3	Husband and Wife	23	0.05
						4	Other	5	0.01
L144B	PERSON WHO DECIDED TO BUY ELEC COOK POT	Num	DECIDE	8	2.50409836	.	Missing	43057	96.71
						1	Husband	260	0.58
						2	Wife	274	0.62
						3	Husband and Wife	862	1.94
						4	Other	68	0.15
L144C	PERSON WHO DECIDED TO BUY PRESSR COOKER	Num	DECIDE	8	2.54756381	.	Missing	42797	96.13
						1	Husband	273	0.61
						2	Wife	314	0.71
						3	Husband and Wife	1057	2.37
						4	Other	80	0.18
L144D	PERSON WHO DECIDED TO BUY COOK TOOLS	Num	DECIDE	8	2.59278351	.	Missing	43551	97.82
						1	Husband	161	0.36
						2	Wife	146	0.33
						3	Husband and Wife	590	1.33
						4	Other	73	0.16

Variable Name	Variable Label (VAR)	VAR Type	VAR Format	VAR Length	Mean	Range of Values	Frequency Category	Frequency	Percent
L144E	COMPUTER: WHO DECIDED TO BUY	Num	DECIDE	8	2.16666667	.	Missing	44503	99.96
						1	Husband	6	0.01
						2	Wife	4	0.01
						3	Husband and Wife	7	0.02
						4	Other	1	0.00
L144F	TELEPHONE: WHO DECIDED TO BUY	Num	DECIDE	8	2.47280335	.	Missing	44282	99.46
						1	Husband	62	0.14
						2	Wife	16	0.04
						3	Husband and Wife	147	0.33
						4	Other	14	0.03
L145	ANY HH MEMBER PAY FOR WEDDING 12 MON?	Num	YESNOUNK	8	0.7124002	.	Missing	16715	37.54
						0	No	8429	18.93
						1	Yes	19323	43.40
						9	Unknown (9)	54	0.12
L145A	# CAMERA'S REC'D AS GIFTS LAST YEAR	Num		8	0.02156334	0-2		742	1.67
L145B	# OF MICROWAVE AS GIFTS LAST YEAR	Num		8	0.08860759	0-1		79	0.18
L145C	# OF ELECTRIC COOKING POT AS GIFTS LAST	Num		8	0.03332099	0-9		2701	6.07
L145D	# OF PRESSURE COOKING AS GIFTS LAST YEAR	Num		8	0.01682243	0-2		2675	6.01
L145E	# OF METAL COOKING TOOLS AS GIFTS LAST Y	Num		8	0.02695167	0-6		1076	2.42
L145F	COMPUTER: # REC'D AS GIFTS LAST YR	Num		8	0.03571429	0-1		56	0.13
L145G	TELEPHONE: # REC'D AS GIFTS LST YR	Num		8	0.01543943	0-2		842	1.89
L146	AMT PAID FOR WEDDING-INCL. GIFTS-12 MON	Num		8	330.196019	-9999-80000		20748	46.60

Variable Name	Variable Label (VAR)	VAR Type	VAR Format	VAR Length	Mean	Range of Values	Frequency Category	Frequency	Percent
L147	ANY HH MEMB PD DOWRY/BRIDE PRICE-12 MON?	Num	YESNOUNK	8	0.09203118	.	Missing	16813	37.76
						0	No	25406	57.07
						1	Yes	2271	5.10
						9	Unknown (9)	31	0.07
L148	AMT PAID FOR DOWRY/BRIDE PRICE--12 MON	Num		8	1713.03379	-9999-99999		5919	13.29
L149	ANY HH MEMB PAID FUNERAL LAST 12 MONTHS?	Num	YESNOUNK	8	0.30493151	.	Missing	29921	67.21
						0	No	10244	23.01
						1	Yes	4344	9.76
						9	Unknown (9)	12	0.03
L150	AMT PAID FOR FUNERAL IN LAST 12 MONTHS	Num		8	-234.68607	-9999-30000		7438	16.71
L151	IN THE PAST 30 DAYS HAS YOUR SENT GIFTS	Num		8	0.28970793	0-9		7190	16.15
L152	WHAT IS THE TOTAL VALUE OF THE M. OR HH	Num		8	-252.34214	-9999-41000		2081	4.67
L152A	YUAN GIVEN AWAY AS GIFTS/MONEY TO CHILD	Num		8	176.94999	-9999-120000		5159	11.59
L152B	YUAN GIVEN AWAY AS GIFTS/MONEY TO PARS	Num		8	-5.1151375	-9999-94000		6071	13.64
L152C	GAVE AWAY GIFTS/MONEY TO CHILD	Num	YESNOUNK	8	0.10661223	.	Missing	35704	80.20
						0	No	7981	17.93
						1	Yes	823	1.85
						9	Unknown (9)	13	0.03
L152D	GAVE AWAY GIFTS/MONEY TO PARENTS	Num	YESNOUNK	8	0.21225184	.	Missing	35706	80.20
						0	No	7064	15.87
						1	Yes	1736	3.90
						9	Unknown (9)	15	0.03
L152E	EDUCATION EXPENSES - YES/NO?	Num	YESNOUNK	8	0.41531896	.	Missing	40069	90.00

Variable Name	Variable Label (VAR)	VAR Type	VAR Format	VAR Length	Mean	Range of Values	Frequency Category	Frequency	Percent
						0	No	2699	6.06
						1	Yes	1741	3.91
						9	Unknown (9)	12	0.03
L152F	EDUCATION EXPENSES - AMOUNT?	Num		8	3995.87694	-9999-80011		1739	3.91
L152G	OTHER GIFT EXPENSE - YES/NO?	Num	YESNOUNK	8	0.37825696	.	Missing	40069	90.00
						0	No	2984	6.70
						1	Yes	1441	3.24
						9	Unknown (9)	27	0.06
L152H	OTHER GIFT EXPENSE - AMOUNT?	Num		8	647.889583	-9999-60500		1440	3.23
L153	# OF WORKING TVS	Num		8	1.21653204	0-10		24389	54.78
L200	DO OWM YOUR HOUSE/APARTMENT?	Num		8	0.90982414	0-1		16490	37.04
L201	DID YOU BUY YOUR HOUSE/APARTMENT?	Num		8	0.71156187	0-1		14790	33.22
L202	DO YOU HAVE A MORTGAGE	Num		8	0.05038314	0-1		10440	23.45
L203	WHAT IS YOUR MONTHLY MORTGAGE PAYMENT?	Num		8	49.2825203	-9999-20000		492	1.11
L204	WHAT WAS YOUR DOWN PAYMENT(YAUN)?	Num		8	21688.9491	-99999-999999		9039	20.30
L205	DID YOU RENT YOUR HOUSE/APARTMENT?	Num		8	0.30160692	0-1		1618	3.63
T1	PROVINCE	Num	PROV	8	37.7817659	11	Beijing, Added 2011	963	2.16
						21	Liaoning, Missed 1997	4256	9.56
						23	Heilongjiang, Added 1997	3401	7.64
						31	Shanghai, Added 2011	963	2.16
						32	Jiangsu	4735	10.64

Variable Name	Variable Label (VAR)	VAR Type	VAR Format	VAR Length	Mean	Range of Values	Frequency Category	Frequency	Percent
						37	Shandong	4713	10.59
						41	Henan	4815	10.82
						42	Hubei	4871	10.94
						43	Hunan	4833	10.86
						45	Guangxi	5013	11.26
						52	Guizhou	4985	11.20
						55	Chongqing, Added 2011	973	2.19
T2	1=URBAN SITE(U) 2=RURAL SITE(R)	Num		8	1.65672379	1-2		44521	100.00
T3	U:1-2=CITY NUM/R:1-4=COUNTY NUM	Num		8	2.17151457	1-4		44521	100.00
T4	U:1-2,5-6,9-10=URB 3-4,7-8,11-12=SUB/R:1,5,9=TWN 2-4,6-8,10-12=VIL	Num		8	2.78097976	1-9		44521	100.00
T5	HOUSEHOLD NUMBER	Num		8	26.7184475	1-180		44521	100.00

Obs	warning	Variable Name	VAR Format	Frequency Category
1	NO OCCURANCES FOR FORMAT CATEGORY:		PROV	Shanxi, Added 2015
2	NO OCCURANCES FOR FORMAT CATEGORY:		PROV	Yunnan, Added 2015
3	NO OCCURANCES FOR FORMAT CATEGORY:		PROV	Zhejiang, Added 2015
4	NO OCCURANCES FOR FORMAT CATEGORY:		TOLIET	None (1989-1997)
5	NO OCCURANCES FOR FORMAT CATEGORY:		YESNOUNK	Unknown (-9)
6	NO OCCURANCES FOR FORMAT CATEGORY:		YR	Missing
