# Supplementary material for: Physical activity, entertainment screen time, and dietary fat profiles and concurrent weight status among school-attending Chinese children and adolescents: CHNS 2004–2011
Source: Front Public Health. 2026 Sep 28;14:1945546. doi: 10.3389/fpubh.2026.1945546 (PMC13654989; doi:10.3389/fpubh.2026.1945546)
Supplement: Supplementary file 4 [file Data_Sheet_3.ZIP › careh_00.pdf]

DATA SET: careh_00 LABEL: CHNS Household Child Care 1991-2006 DATE CREATED: 27FEB14:09:34:29
Number of Observations: 3172
Number of Variables: 43
Organization of file: 1 Observation Household/Year (HHID/WAVE)

Variable Name	Variable Label (VAR)	VAR Type	VAR Format	VAR Length	Mean	Range of Values	Frequency Category	Frequency	Percent
HHID	HOUSEHOLD ID	Num		8	381121633	211101003 to 522404041		3172	100.00
COMMID	COMMUNITY ID: T1-T4	Num		8	381121.616	211101-522404		3172	100.00
WAVE	SURVEY YEAR	Num	YR	8	1995.66992	1989	1989	993	31.31
						1991	1991	322	10.15
						1993	1993	326	10.28
						1997	1997	354	11.16
						2000	2000	501	15.79
						2004	2004	313	9.87
						2006	2006	363	11.44
K14_HH	0-1.5: KM NEAREST STATE CC CENTER	Num		8	2.0125	0-60.20		56	1.77
K15_HH	1.5-3.5: KM NEAREST STATE CC CENTER	Num		8	1.159375	0-30		96	3.03
K16_HH	3.5-6.5: KM NEAREST STATE CC CENTER	Num		8	0.87112971	0-30		239	7.53
K17_HH	0-1.5: KM NEAREST PRIV. CC CENTER	Num		8	3.4	0-31		12	0.38
K18_HH	1.5-3.5: KM NEAREST PRIV. CC CENTER	Num		8	2.90769231	0-30		13	0.41
K19_HH	3.5-6.5: KM NEAREST PRIV. CC CENTER	Num		8	1.82727273	0-30		22	0.69
K20_HH	0-1.5: KM NEAREST WORK-U CC CENTER	Num		8	0.96818182	0-3		22	0.69
K21_HH	1.5-3.5: KM NEAREST WORK-U CC CENTER	Num		8	1.46666667	0-6		45	1.42
K22_HH	3.5-6.5: KM NEAREST WORK-U CC CENTER	Num		8	0.7630137	0-5		73	2.30
K23_HH	0-1.5: COST/MONTH STATE CC CENTER	Num		8	22.1967742	0-68		31	0.98

DATA SET: careh_00 LABEL: CHNS Household Child Care 1991-2006 DATE CREATED: 27FEB14:09:34:29
Number of Observations: 3172
Number of Variables: 43
Organization of file: 1 Observation Household/Year (HHID/WAVE)

Variable Name	Variable Label (VAR)	VAR Type	VAR Format	VAR Length	Mean	Range of Values	Frequency Category	Frequency	Percent
K24_HH	1.5-3.5: COST/MONTH STATE CC CENTER	Num		8	18.2461538	0-60		78	2.46
K25_HH	3.5-6.5: COST/MONTH STATE CC CENTER	Num		8	14.475814	0-66		215	6.78
K26_HH	0-1.5: COST/MONTH PRIV. CC CENTER	Num		5	39.7777778	8-60		9	0.28
K27_HH	1.5-3.5: COST/MONTH PRIV. CC CENTER	Num		5	25.125	0-50		8	0.25
K28_HH	3.5-6.5: COST/MONTH PRIV. CC CENTER	Num		5	11.9333333	0-39		15	0.47
K29_HH	0-1.5: COST/MONTH WORK-U CC CENTER	Num		5	21.6666667	0-60		15	0.47
K30_HH	1.5-3.5: COST/MONTH WORK-U CC CENTER	Num		8	19.759375	0-80		32	1.01
K31_HH	3.5-6.5: COST/MONTH WORK-U CC CENTER	Num		8	18.628169	0-125		71	2.24
K32_HH	KIDS CARED FOR NON-HH M PAST WEEK?	Num	YESNOUNK	5	0.38626609	.	Missing	2240	70.62
						0	No	572	18.03
						1	Yes	360	11.35
K33_HH	# KIDS CARED FOR NON-HH M PAST WEEK	Num		5	1.11747851	0-3		349	11.00
K34_HH	NON-HH CHILD CARE: HH ITSELF?	Num	HH_CARE	5	1	.	Missing	2738	86.32
						1-7	Yes	434	13.68
K35_HH	NON-HH CHILD CARE: RELATIVE'S HOME?	Num	HH_CARE	5	2	.	Missing	3079	97.07
						1-7	Yes	93	2.93
K36_HH	NON-HH CHILD CARE: NEIGHBOR'S HOME?	Num	HH_CARE	5	3	.	Missing	3155	99.46
						1-7	Yes	17	0.54
K37_HH	NON-HH CHILD CARE: INFANT CARE CTR?	Num	HH_CARE	5			Missing	3172	100.00

DATA SET: careh_00 LABEL: CHNS Household Child Care 1991-2006 DATE CREATED: 27FEB14:09:34:29
Number of Observations: 3172
Number of Variables: 43
Organization of file: 1 Observation Household/Year (HHID/WAVE)

Variable Name	Variable Label (VAR)	VAR Type	VAR Format	VAR Length	Mean	Range of Values	Frequency Category	Frequency	Percent
K38_HH	NON-HH CHILD CARE: NURSERY SCHOOL?	Num	HH_CARE	5	5	.	Missing	3117	98.27
						1-7	Yes	55	1.73
K39_HH	NON-HH CHILD CARE: DAY CARE?	Num	HH_CARE	5	6	.	Missing	3055	96.31
						1-7	Yes	117	3.69
K40_HH	NON-HH CHILD CARE: OTHER?	Num	HH_CARE	5	7	.	Missing	3163	99.72
						1-7	Yes	9	0.28
K41_HH	HRS/DAY CHILD CARE NON-HH TYPICALLY	Num		5	8.04427083	0-24		384	12.11
K42_HH	DAYS/WK CHILD CARE NON-HH TYPICALLY	Num		5	3.99408284	0-7		507	15.98
K43	HOW MUCH HH PAYS/MTH FOR CHILD CARE	Num		5	53.7466384	-99-1600		2826	89.09
K44	# KIDS RECEIVING CARE K43 PAYMENT	Num		5	0.63286845	0-9		2653	83.64
K44A	DOES HH PAY FOR CHILD CARE?	Num	YESNOUNK	8	0.93491124	.	Missing	2496	78.69
						0	No	44	1.39
						1	Yes	632	19.92
K45	CHILD CARE/NURSERY SUBSIDY PROV. HH?	Num	YESNOUNK	5	0.23743207	.	Missing	228	7.19
						0	No	2581	81.37
						1	Yes	321	10.12
						9	Unknown (9)	42	1.32
K46	HOW MUCH SUBSIDY/MONTH (YUAN)	Num		8	9.5586	-9-99		500	15.76
K47	# CHILDREN SUBSIDY PROVIDED	Num		5	0.53895072	0-5		629	19.83
T1	PROVINCE	Num	PROV	8	37.9249685	21	Liaoning, Missed 1997	366	11.54

DATA SET: careh_00 LABEL: CHNS Household Child Care 1991-2006 DATE CREATED: 27FEB14:09:34:29
Number of Observations: 3172
Number of Variables: 43
Organization of file: 1 Observation Household/Year (HHID/WAVE)

Variable Name	Variable Label (VAR)	VAR Type	VAR Format	VAR Length	Mean	Range of Values	Frequency Category	Frequency	Percent
						23	Heilongjiang, Added 1997	213	6.72
						32	Jiangsu	394	12.42
						37	Shandong	363	11.44
						41	Henan	390	12.30
						42	Hubei	406	12.80
						43	Hunan	295	9.30
						45	Guangxi	399	12.58
						52	Guizhou	346	10.91
T2	1=URBAN SITE(U) 2=RURAL SITE(R)	Num		8	1.64911728	1-2		3172	100.00
T3	U:1-2=CITY NUM/R:1-4=COUNTY NUM	Num		8	2.20208071	1-4		3172	100.00
T4	U:1-2,5-6,9-10=URB 3-4,7-8,11-12=SUB/R:1,5,9=TWN 2-4,6-8,10-12=VIL	Num		8	2.60592686	1-9		3172	100.00
T5	HOUSEHOLD NUMBER	Num		8	17.2840479	1-113		3172	100.00

Obs	warning	Variable Name	VAR Format	Frequency Category
1	NO OCCURANCES FOR FORMAT CATEGORY:		PROV	Beijing, Added 2011
2	NO OCCURANCES FOR FORMAT CATEGORY:		PROV	Chongqing, Added 2011
3	NO OCCURANCES FOR FORMAT CATEGORY:		PROV	Shanghai, Added 2011
4	NO OCCURANCES FOR FORMAT CATEGORY:		YESNOUNK	Unknown (-9)
5	NO OCCURANCES FOR FORMAT CATEGORY:		YR	2009
6	NO OCCURANCES FOR FORMAT CATEGORY:		YR	2011
7	NO OCCURANCES FOR FORMAT CATEGORY:		YR	Missing
8	All Non-Missing values are the same for:	K34_HH	HH_CARE	Missing
9	All Non-Missing values are the same for:	K34_HH	HH_CARE	Yes
10	All Non-Missing values are the same for:	K35_HH	HH_CARE	Missing
11	All Non-Missing values are the same for:	K35_HH	HH_CARE	Yes
12	All Non-Missing values are the same for:	K36_HH	HH_CARE	Missing
13	All Non-Missing values are the same for:	K36_HH	HH_CARE	Yes
14	All values are missing for:	K37_HH	HH_CARE	Missing
15	All Non-Missing values are the same for:	K38_HH	HH_CARE	Missing
16	All Non-Missing values are the same for:	K38_HH	HH_CARE	Yes
17	All Non-Missing values are the same for:	K39_HH	HH_CARE	Missing
18	All Non-Missing values are the same for:	K39_HH	HH_CARE	Yes
19	All Non-Missing values are the same for:	K40_HH	HH_CARE	Missing
20	All Non-Missing values are the same for:	K40_HH	HH_CARE	Yes
