# Supplementary material for: Physical activity, entertainment screen time, and dietary fat profiles and concurrent weight status among school-attending Chinese children and adolescents: CHNS 2004–2011
Source: Front Public Health. 2026 Sep 28;14:1945546. doi: 10.3389/fpubh.2026.1945546 (PMC13654989; doi:10.3389/fpubh.2026.1945546)
Supplement: Supplementary file 4 [file Data_Sheet_3.ZIP › cropt_00.pdf]

DATA SET: cropt_00 LABEL: CHNS Household Crop File 1989-1997 DATE CREATED: 27FEB14:09:51:34
Number of Observations: 21542
Number of Variables: 30
Organization of file: 1 Observation Household/Year/CROP Type (HHID/WAVE/E11)

Variable Name	Variable Label (VAR)	VAR Type	VAR Format	VAR Length	Mean	Range of Values	Frequency Category	Frequency	Percent
HHID	HOUSEHOLD ID	Num		8	403842050	211103016 to 522404043		21542	100.00
COMMID	COMMUNITY ID: T1-T4	Num		8	403842.039	211103-522404		21542	100.00
WAVE	SURVEY YEAR	Num	YR	8	1992.17473	1989	1989	6191	28.74
						1991	1991	5825	27.04
						1993	1993	4867	22.59
						1997	1997	4659	21.63
E11	CROP TYPE NUMBER	Num		8	2.07144183	1-6		21542	100.00
E13	# OF KG OF CROP GROWN LAST YEAR	Num		8	992.718235	0-11700		15147	70.31
E13B	12-MO: AMT SPENT ON RAISING THIS CROP	Num		6	135.132553	0-1862		5978	27.75
E13B_CLN	E13B OUTLYING VALUE - REVISED	Num	FLAG	8	1	.	Missing	21532	99.95
						1	Yes	10	0.05
E13_CLN	E13 OUTLYING VALUE - REVISED	Num	FLAG	8	1	.	Missing	21531	99.95
						1	Yes	11	0.05
E14	# OF KG OF CROP SOLD TO GOVT LAST YEAR	Num		8	159.640735	0-9998		14631	67.92
E14B	12-MO: PRODUCE FROM HH FARM CROP SOLD?	Num	YESNO	6	0.38603896	.	Missing	15382	71.40
						0	No	3782	17.56
						1	Yes	2378	11.04
E14_CLN	E14 OUTLYING VALUE - REVISED	Num	FLAG	8	1	.	Missing	21532	99.95
						1	Yes	10	0.05
E15	GOVT PRICE (YUAN/KG) FOR CROP LAST YR	Num		8	1.00735553	0-9		8168	37.92

DATA SET: cropt_00 LABEL: CHNS Household Crop File 1989-1997 DATE CREATED: 27FEB14:09:51:34
Number of Observations: 21542
Number of Variables: 30
Organization of file: 1 Observation Household/Year/CROP Type (HHID/WAVE/E11)

Variable Name	Variable Label (VAR)	VAR Type	VAR Format	VAR Length	Mean	Range of Values	Frequency Category	Frequency	Percent
E15B	12-MO: AMT RECD--SALE OF HH FARM CROP	Num		6	409.933105	1-4000		2332	10.83
E15B_CLN	E15B OUTLYING VALUE - REVISED	Num	FLAG	8	1	.	Missing	21533	99.96
						1	Yes	9	0.04
E15_CLN	E15 OUTLYING VALUE - REVISED	Num	FLAG	8	1	.	Missing	21538	99.98
						1	Yes	4	0.02
E16	# OF KG OF CROP SOLD TO FREE MKT LST YR	Num		8	212.533555	0-9990		14272	66.25
E16B	12-MO: PRODUCE FROM HH FARM CROP KEPT?	Num	YESNO	6	0.89990266	.	Missing	15378	71.39
						0	No	617	2.86
						1	Yes	5547	25.75
E16_CLN	E16 OUTLYING VALUE - REVISED	Num	FLAG	8	1	.	Missing	21522	99.91
						1	Yes	20	0.09
E17	FREE MKT PRICE (YUAN/KG) FOR CROP LST YR	Num		8	1.1214084	0-9.80		8144	37.81
E17B	12-MO: AMT RECD IF KEPT-FARM-CROP SOLD	Num		6	395.057887	0-3500		5414	25.13
E17B_CLN	E17B OUTLYING VALUE - REVISED	Num	FLAG	8	1	.	Missing	21460	99.62
						1	Yes	82	0.38
E17_CLN	E17 OUTLYING VALUE - REVISED	Num	FLAG	8	1	.	Missing	21526	99.93
						1	Yes	16	0.07
E18B	12-MO: PRODUCE FROM HH FARM CROP GIVEN?	Num	YESNO	6	0.06024683	.	Missing	15384	71.41
						0	No	5787	26.86
						1	Yes	371	1.72
E19B	12-MO: AMT RECD IF GIVEN-FARM-CROP SOLD	Num		6	78.4929972	2-500		357	1.66

DATA SET: cropt_00 LABEL: CHNS Household Crop File 1989-1997 DATE CREATED: 27FEB14:09:51:34
Number of Observations: 21542
Number of Variables: 30
Organization of file: 1 Observation Household/Year/CROP Type (HHID/WAVE/E11)

Variable Name	Variable Label (VAR)	VAR Type	VAR Format	VAR Length	Mean	Range of Values	Frequency Category	Frequency	Percent
E19B_CLN	E19B OUTLYING VALUE - REVISED	Num	FLAG	8	1	.	Missing	21540	99.99
						1	Yes	2	0.01
T1	PROVINCE	Num	PROV	8	40.171154	21	Liaoning, Missed 1997	1490	6.92
						23	Heilongjiang, Added 1997	480	2.23
						32	Jiangsu	3156	14.65
						37	Shandong	2195	10.19
						41	Henan	3610	16.76
						42	Hubei	2643	12.27
						43	Hunan	1575	7.31
						45	Guangxi	2621	12.17
						52	Guizhou	3772	17.51
T2	1=URBAN SITE(U) 2=RURAL SITE(R)	Num		8	1.88167301	1-2		21542	100.00
T3	U:1-2=CITY NUM/R:1-4=COUNTY NUM	Num		8	2.45798904	1-4		21542	100.00
T4	U:1-2,5-6,9-10=URB 3-4,7-8,11-12=SUB/R:1,5,9=TWN 2-4,6-8,10-12=VIL	Num		8	3.02710983	1-8		21542	100.00
T5	HOUSEHOLD NUMBER	Num		8	11.1214836	1-48		21542	100.00

Obs	warning	Variable Name	VAR Format	Frequency Category
1	NO OCCURANCES FOR FORMAT CATEGORY:		PROV	Beijing, Added 2011
2	NO OCCURANCES FOR FORMAT CATEGORY:		PROV	Chongqing, Added 2011
3	NO OCCURANCES FOR FORMAT CATEGORY:		PROV	Shanghai, Added 2011
4	NO OCCURANCES FOR FORMAT CATEGORY:		YESNO	Unknown or Invalid Response
5	NO OCCURANCES FOR FORMAT CATEGORY:		YR	2000
6	NO OCCURANCES FOR FORMAT CATEGORY:		YR	2004
7	NO OCCURANCES FOR FORMAT CATEGORY:		YR	2006
8	NO OCCURANCES FOR FORMAT CATEGORY:		YR	2009
9	NO OCCURANCES FOR FORMAT CATEGORY:		YR	2011
10	NO OCCURANCES FOR FORMAT CATEGORY:		YR	Missing
