# Supplementary material for: Physical activity, entertainment screen time, and dietary fat profiles and concurrent weight status among school-attending Chinese children and adolescents: CHNS 2004–2011
Source: Front Public Health. 2026 Sep 28;14:1945546. doi: 10.3389/fpubh.2026.1945546 (PMC13654989; doi:10.3389/fpubh.2026.1945546)
Supplement: Supplementary file 4 [file Data_Sheet_3.ZIP › busn_12.pdf]

DATA SET: busn_12 LABEL: CHNS Household Small busnness Income 1989-2011 DATE CREATED: 01MAY18:15:27:25
Number of Observations: 7754
Number of Variables: 14
Organization of file: 1 Observation per Household/Year/busnness (HHID/WAVE/HHBUSNUM)

Variable Name	Variable Label (VAR)	VAR Type	VAR Format	VAR Length	Mean	Range of Values	Frequency Category	Frequency	Percent
HHID	HOUSEHOLD ID	Num		8	402882967	111301020 to 552304010		7754	100.00
COMMID	COMMUNITY ID: T1-T4	Num		8	402882.943	111301-552304		7754	100.00
WAVE	SURVEY YEAR	Num	YR	8	2001.56796	1989	1989	582	7.51
						1991	1991	706	9.10
						1993	1993	667	8.60
						1997	1997	904	11.66
						2000	2000	982	12.66
						2004	2004	954	12.30
						2006	2006	876	11.30
						2009	2009	819	10.56
						2011	2011	777	10.02
						2015	2015	487	6.28
H2	TYPE OF SMALL BUSINESS	Num	BUS_TYPE	8	2.78511327	.	Missing	29	0.37
						1	Commerce	2421	31.22
						2	Service	2156	27.81
						3	Manufacturing	609	7.85
						4	Peddler	705	9.09
						5	Construction	869	11.21
						6	Other	965	12.45
H3	AVERAGE MONTHLY BUSINESS REVENUES	Num		8	1524.01134	-9999-99999		7706	99.38
H3_CLN	H3 OUTLYING VALUE - REVISED	Num	FLAG	8	1	.	Missing	7641	98.54
						1	Yes	113	1.46
H4	AVERAGE MONTHLY BUSINESS EXPENSES	Num		8	-356.79128	-9999-90000		7342	94.69

DATA SET: busn_12 LABEL: CHNS Household Small busnness Income 1989-2011 DATE CREATED: 01MAY18:15:27:25
Number of Observations: 7754
Number of Variables: 14
Organization of file: 1 Observation per Household/Year/busnness (HHID/WAVE/HHBUSNUM)

Variable Name	Variable Label (VAR)	VAR Type	VAR Format	VAR Length	Mean	Range of Values	Frequency Category	Frequency	Percent
H4_CLN	H4 OUTLYING VALUE - REVISED	Num	FLAG	8	1	.	Missing	7600	98.01
						1	Yes	154	1.99
METHOD	METHOD USED TO ROLL UP REV/EXP TO BUSINESS - SEE DOCUMENTATION	Num		8	0.34020619	0-4		582	7.51
T1	PROVINCE	Num	PROV	8	40.0842146	11	Beijing, Added 2011	15	0.19
						21	Liaoning, Missed 1997	765	9.87
						23	Heilongjiang, Added 1997	245	3.16
						31	Shanghai, Added 2011	8	0.10
						32	Jiangsu	809	10.43
						37	Shandong	612	7.89
						41	Henan	821	10.59
						42	Hubei	854	11.01
						43	Hunan	918	11.84
						45	Guangxi	1415	18.25
						52	Guizhou	1190	15.35
						55	Chongqing, Added 2011	102	1.32
T2	1=URBAN SITE(U) 2=RURAL SITE(R)	Num		8	1.80719629	1-2		7754	100.00
T3	U:1-2=CITY NUM/R:1-4=COUNTY NUM	Num		8	2.30900181	1-4		7754	100.00
T4	U:1-2,5-6,9-10=URB 3-4,7-8,11-12=SUB/R:1,5,9=TWN 2-4,6-8,10-12=VIL	Num		8	2.70028372	1-9		7754	100.00

DATA SET: busn_12 LABEL: CHNS Household Small busnness Income 1989-2011 DATE CREATED: 01MAY18:15:27:25
Number of Observations: 7754
Number of Variables: 14
Organization of file: 1 Observation per Household/Year/busnness (HHID/WAVE/HHBUSNUM)

Variable Name	Variable Label (VAR)	VAR Type	VAR Format	VAR Length	Mean	Range of Values	Frequency Category	Frequency	Percent
T5	HOUSEHOLD NUMBER	Num		8	24.2768893	1-176		7754	100.00

Obs	warning	Variable Name	VAR Format	Frequency Category
1	NO OCCURANCES FOR FORMAT CATEGORY:		PROV	Shanxi, Added 2015
2	NO OCCURANCES FOR FORMAT CATEGORY:		PROV	Yunnan, Added 2015
3	NO OCCURANCES FOR FORMAT CATEGORY:		PROV	Zhejiang, Added 2015
4	NO OCCURANCES FOR FORMAT CATEGORY:		YR	Missing
